# Steering the multiexciton generation in slip-stacked perylene dye array via exciton coupling

**DOI:** 10.1038/s41467-022-31958-1

**Published:** 2022-08-02

**Authors:** Yongseok Hong, Maximilian Rudolf, Munnyon Kim, Juno Kim, Tim Schembri, Ana-Maria Krause, Kazutaka Shoyama, David Bialas, Merle I. S. Röhr, Taiha Joo, Hyungjun Kim, Dongho Kim, Frank Würthner

**Affiliations:** 1 Spectroscopy Laboratory for Functional π-Electronic Systems and Department of Chemistry, Yonsei University, Seoul, 03722 Republic of Korea; 2 Universitat Würzburg, Institut für Organische Chemie, Am Hubland, 97074 Würzburg, Germany; 3 Department of Chemistry, Pohang University of Science and Technology (POSTECH), Pohang, 37673 Republic of Korea; 4 Universität Würzburg, Center for Nanosystems Chemistry, Theodor-Boveri Weg, 97074 Würzburg, Germany; 5 Department of Chemistry and Research Institute of Basic Sciences, Incheon National University, Incheon, 22012 Republic of Korea; 6 Division of Energy Materials, Pohang University of Science and Technology (POSTECH), Pohang, 37673 Republic of Korea

**Keywords:** Excited states, Self-assembly

## Abstract

Dye arrays from dimers up to larger oligomers constitute the functional units of natural light harvesting systems as well as organic photonic and photovoltaic materials. Whilst in the past decades many photophysical studies were devoted to molecular dimers for deriving structure-property relationship to unravel the design principles for ideal optoelectronic materials, they fail to accomplish the subsequent processes of charge carrier generation or the detachment of two triplet species in singlet fission (SF). Here, we present a slip-stacked perylene bisimide trimer, which constitutes a bridge between hitherto studied dimer and solid-state materials, to investigate SF mechanisms. This work showcases multiple pathways towards the multiexciton state through direct or excimer-mediated mechanisms by depending upon interchromophoric interaction. These results suggest the comprehensive role of the exciton coupling, exciton delocalization, and excimer state to facilitate the SF process. In this regard, our observations expand the fundamental understanding the structure-property relationship in dye arrays.

## Abstract (web summary)

Understanding structure-property relationship of dye arrays is of great importance for designing organic photonic and photovoltaic materials. Here, authors present a slip-stacked perylene bisimide array as a model system to investigate singlet fission mechanisms by depending upon interchromophoric interaction.

## Introduction

Intermolecular interaction in organic semiconductors (OSCs), described by Coulomb coupling and charge-transfer (CT) coupling contributions, is of relevance for the fate of the exciton^1,2^. One of the most interesting light-induced phenomena in OSCs is singlet fission (SF), enabling the conversion from a singlet exciton into two triplet excitons via a multiexciton (ME) state^3–8^. Such exciton multiplication process provides the breaking route to mitigate the thermalization loss (~33%) in solar energy technologies^9–13^. In practical aspects, in particular endothermic SF systems as observed for perylene bisimide (PBI) dyes are of great relevance for the application in photovoltaics, as the triplet energy level of these materials is comparable to the bandgap of silicon (1.1 eV). Among the most well-studied endothermic SF systems^14–17^, perylene bisimide (PBI) shows the advantages of high molar extinction (<10^5^ M^−1^cm^−1^), photostability, and lightfastness, suggesting that the PBI is an ideal SF material^18,19^. In this regard, there have been extensive theoretical works to investigate the SF mechanism in PBIs and to realize a molecular packing geometry for the ultrafast/efficient SF^20–22^. However, in slip-stacked PBIs, the intermolecular interaction, especially the CT coupling, is hyper-sensitive to molecular packing geometry^23,24^. Furthermore, the energetic proximity between Frenkel exciton (FE) and CT states in slip-stacked PBI scaffold generally gives rise to the excimer formation^25,26^. Due to such complexities, understanding the underlying mechanism in PBIs has remained challenging.

**Fig. 1:**
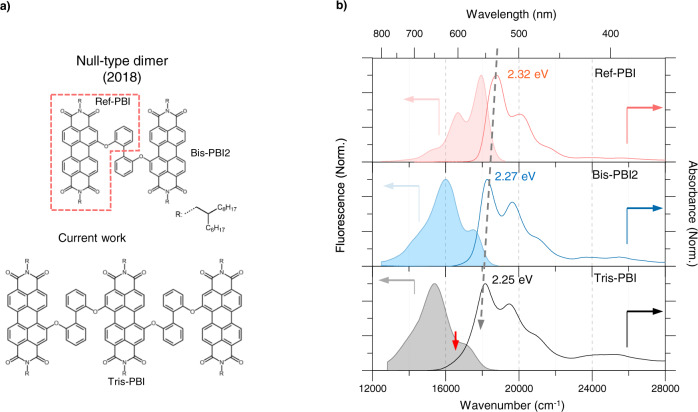
The molecular structure and basic optical properties of Tris-PBI. **a** Schematic molecular structure of **Ref-PBI**, **Bis-PBI2**, and **Tris-PBI** with the branched 2-hexyldecyl substituents originating from racemic amine precursor. **b** The normalized steady-state absorption (solid) and fluorescence (filled area) spectra of **Ref-PBI** (top), **Bis-PBI2** (middle) and **Tris-PBI** (bottom) in TOL upon photoexcitation at 500, 510, and 550 nm, respectively. The red arrow indicates the red-edge band of **Tris-PBI**.

Recently, scrupulous studies by Roberts and co-workers^27,28^ revealed that the CT coupling, i.e., CT-mediated mechanism, is of high relevance for the SF mechanism in PBI films. Nevertheless, the fastest SF in PBI films occurs on the timescale (*k*_MEG_ = (270 ps)^−1^), which is 1000 times slower than theoretically expected value (*k*_MEG_ = (50 fs)^−1^ to (few ps)^−1^) due to excessive stabilization of the S_1_ energy. Furthermore, there has been a dispute over the role of excimer state, while some consensus has been reached over its working mechanisms such as the direct mechanism and charge-transfer mediation^29–36^. In this regard, the following questions have remained: (1) an appropriate packing structure for ultrafast SF process, (2) the detailed SF mechanism, (3) the role of the excimer. One way to understand such underlying mechanisms is to investigate the molecular model compound^37–44^. Indeed, extensive works by our team and Wasielewski group have investigated the excited-state dynamics such as symmetry-breaking charge separation (SBCS), excimer formation, or SF using PBI dimers^13,33^. Especially, we recently demonstrated in the null-type PBI dimer tethered by a flexible linker (**Bis-PBI2**, Fig. 1a) that SF process occurs on picosecond timescale (*k*_MEG_ = (370 ps)^−1^ to (20 ps)^−1^) via a direct CT-mediated mechanism^45^. However, the underlying SF mechanism has not been clearly illustrated as the hitherto dimers not only do not represent complex situations, i.e., competition between SF and the excimer formation, but also can not describe delocalized exciton beyond the dimer limit. From theoretical side, trimer stacks have been employed in order to study the role of exciton delocalization, as well as the impact of the crystal packing on the energetics and coupling terms^46–52^.

Here, we describe a null-type PBI trimer (**Tris-PBI**, Fig. 1a) that bridges the understanding of SF dynamics in hitherto studied dimer and solid materials. Our nullification strategy, i.e. designing a packing arrangement with counteracting Coulomb and CT coupling contributions, provides two important features: First, the distribution of CT configurations over the ensembles’ electronic manifold via strong CT coupling and second, the compensation of CT and Coulomb couplings to prevent the relaxation of the excited state to a lower energy band edge state, thereby supporting the energetic requirement for SF (E_b_ = E(S_1_)−2xE(T_1_)). Indeed, the X-ray crystal structure of **Tris-PBI** indicates that the packing geometry of **Tris-PBI** corresponds to the ideal geometry for SF calculated by Roberts^28^. Combining the comprehensive electronic and vibrational spectroscopies, we observe that the SF (which will be called MEG throughout the remaining context) can occur as an ultrafast MEG process (*k*_SF_ > (500 fs)^−1^) in delocalized **Tris-PBI** upon photoexcitation at the red-edge CT band. Quantum simulations suggest that this ultrafast SF process is attributable to the large charge resonance (CR) contribution in the S_1_ state that leads to the direct coupling between S_1_ and ME states. In contrast, photoexcitation at the energetically higher exciton states proceeds into a localized excimer state that still can mediate the MEG process on a slower time-scale in **Tris-PBI**. Since we showcase the comprehensive role of intermolecular interaction, exciton delocalization, and excimer state, our observations provide important insights into the MEG mechanism in slip-stacked PBI trimer, which are of relevance for the development of PBI-based SF devices.

## Results and discussion

### Molecular structure, exciton coupling, and basic optical properties

The synthesis of **Tris-PBI** was accomplished from bay-mono- and bay-dibrominated PBI precursors following our previously reported coupling method for various Bis-PBIs^53^ that relies on the nucleophilic aromatic substitution of bromine substituents by 2,2’-biphenol (for details, see Supplementary Information). To ensure a high solubility even in solvents of low polarity, we introduced branched 2-hexyldecyl substituents at the imide nitrogens from the respective racemic amine. Like for the previously investigated **Bis-PBI2** also **Tris-PBI** shows a monomer-like absorption profile in the steady-state absorption spectrum in toluene (TOL), indicating the null-type exciton coupling (Fig. 1b). However, **Tris -PBI** shows a prominent red-edge tail compared to the monomer (**Ref-PBI**) and the previously investigated null-type dimer **Bis-PBI2**^45^. In contrast to the weakly changed monomer-like absorption spectrum of **Tris-PBI**, the fluorescence spectrum of **Tris-PBI** shows in addition to the FE emission at 580 nm the multiexcitonic emission at 640 nm, quite similar to that observed for the null-type PBI dimer **Bis-PBI2** (Fig. 1b and Supplementary Fig. 11)^30,45,54^. Solvent-polarity-dependent experiments rationalize the strong CT coupling of **Tris-PBI** in various solvents (tetrahydrofuran, THF; dichloromethane, DCM; benzonitrile, BCN, Figs. S10, S11). The absorption spectrum of **Tris-PBI** shows a distinct CT band irrespective of the solvent polarity, which indicates the sufficient CT coupling without the help of solvation. In contrast, the increase in the dielectric constant leads to a gradual red-shift in the fluorescence spectrum and the decrease of the fluorescence quantum yield (0.18, TOL; 0.05 in THF; 0.02 in DCM; 0.02 in BCN). This feature infers the further energetic stabilization as well as the opening of the additional excited-state relaxation pathways by dipolar solvation processes. Furthermore, the increased fluorescence quantum yield (~0.4) in viscous paraffin indicates that the structural dynamics is strongly coupled to the excited-state relaxation pathways. Further, because the overall trend of excitation-energy dependent fluorescence spectra in viscous paraffin is in good agreement with that in toluene, structural heterogeneity can be ruled out. Accordingly, we provide an alternative explanation on the excitation energy-dependent excited state dynamics in terms of electronic manifolds within the trimer stack. In this respect, our further experiments are performed using the pump sources at 550 and 600 nm, which are labeled as “HP” (the higher energy pumping) and “LP” (the lower energy pumping), respectively for clarity.

**Fig. 2:**
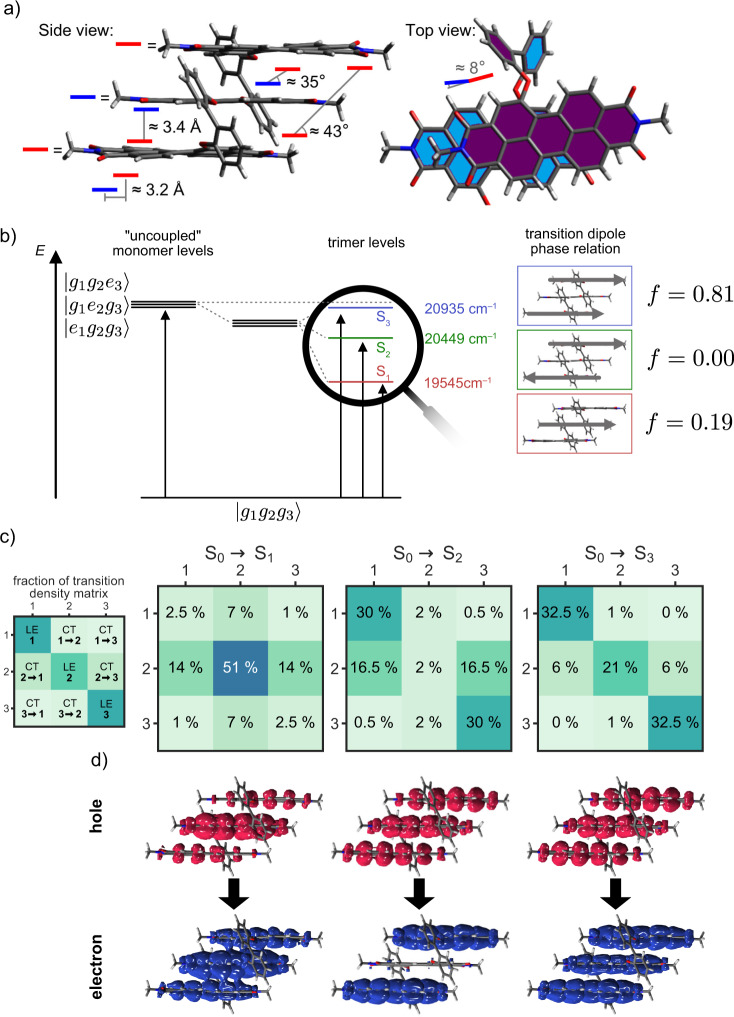
X-ray crystal structure and exciton coupling of Tris-PBI. **a** Molecular structure of **Tris-PBI** in single crystals with 2 -hexyldecyl groups replaced by methyl groups for clarity. **b** calculated optical transitions with transition dipole phase relation (exciton coupling) for the crystal structure. The *g* and *e* indicate the ground and excited states, respectively. Energies and oscillator strengths are obtained from TD-DFT gas phase calculation. **c**, **d** Normalized one particle transition density matrices (TDMs, (**c**) and corresponding electron and hole densities (**d**) for the excited state transitions S_0_ → S_1_, S_2_ and S_3_ as determined by TD-DFT calculations based on optimized DFT structure. The isosurface values in (**c**) are set to 0.015 a.u.

Before reporting on the outcome of these spectroscopic studies, we like to clarify the structural features of **Tris-PBI**. Due to the difficulties encountered for the crystallization of such dye oligomers our previous research on various Bis-PBIs^45,55^ as well as related work from other groups on multichromophoric dye ensembles^33,56–58^ relied on calculated structures. Such calculations are, however, prone to errors because of the large size of these molecular systems and the concomitant limitations for the applicable level of theoretical treatment. In this regard, **Tris-PBI** constitutes a particular lucky case as we accomplished to grow pyramidal red crystals upon diffusion of methanol (bad solvent) into a solution of **Tris-PBI** in toluene (good solvent) that were suitable for the evaluation of the π−π-stacking arrangement of the PBIs (Fig. 2a and Supplementary Fig. 10). The successful crystallization of **Tris-PBI** is indeed quite surprising if we consider the presence of extended branched alkyl chains at the imide nitrogens and that the utilized 2-hexyldecylamine precursor was racemic, thereby leading to the presence of a multitude of diastereomers of **Tris-PBI**. The most likely explanation for the nevertheless high quality crystals is that the two branches of alkyl chains (hexyl, octyl) are not very different and thereby exchangeable in the packing arrangement^59^ and that the structure of the triple PBI stack is well-defined both in the solid state and in solution. This view is supported by two-dimensional H,H-COSY and -ROESY as well as DOSY NMR studies in 1,1,2,2-tetrachloroethane (see supplementary information, Supplementary Table 1) that show the expected chemical shifts and intermolecular ROE effects for a slip-stacked **Tris-PBI** architecture. According to our crystallographic analysis for **Tris-PBI** the 2,2’-biphenol spacer units enforce a longitudinal slip of 3.2 Å between perfectly π-stacked neighboring PBIs at ideal van-der-Waals distances of 3.4 Å (Fig. 2a, for details see Supplementary Table 5). The almost perfect co-planarity of the PBIs and only modest rotational offset of ~8° discloses a quite perfect molecular design for SF. Thus, referring to the previous calculations by Roberts for a variety of PBI solid state materials^28^, these structural parameters manifest that the packing structure of **Tris-PBI** is located close to the ideal geometry for the MEG process observed in PBI thin films (i.e., 3.2 Å in longitudinal shift and ~0 Å in transversal shift). Furthermore, the tight packing structure of **Tris-PBI** hinders any significant change of molecular structure, which is important for understanding the structure-property relationship.

To gain further insight into the electronic coupling between the three PBI dyes in the given slip-stacked arrangement for **Tris-PBI**, as a first step, we performed time-dependent density functional theory (TDDFT) calculations based on the crystal structure in order to determine the long-range Coulomb (*J*_coul_) and short-range CT coupling (*J*_CT_). These calculations confirm an almost perfect counteraction of Coulomb (470 cm^−1^) and charge transfer couplings (−499 cm^−1^) between the respective neighboring dyes with an additional weak Coulomb coupling (−78 cm^−1^) between the two distant PBI dyes (see supplementary information, Supplementary Table 5). As shown in Fig. 2b, this additional coupling leads to a situation that distinguishes trimers considerably from the previously investigated dimers. Thus, the three lowest exciton states are of slightly different energy and only the weakly allowed S_0_→S_1_ transition corresponds to a perfect null-coupling situation where primarily the central chromophore is excited into its LE state. The two “outer” molecules form two H-type exciton states that are only slightly split in energy due to the weak Coulomb coupling. Optimization of the crystal structure employing DFT leads to small geometric changes that introduce additional electronic coupling between the individual monomers: As shown in Fig. 2c, for the S_1_ state, the calculated transition density matrix indicates significant CT from the central molecule to the outer ones, confirmed by the electron and hole pair densities provided in Fig. 2d. However, the high oscillator strength bearing dominant transition in the absorption spectra (Fig. 1b) corresponds to the S_0_→S_3_ transition that originates according to our TDM analysis from excitation of a delocalized exciton state. As we will show in the following, the excited state dynamics of **Tris-PBI** strongly depend on the respective excitations of these two states with their rather different CT contributions as already suggested by the steady-state fluorescence spectra in Fig. 1b.

### Multiexciton generation and relaxation processes upon photoexcitation at higher and lower energy states

**Fig. 3:**
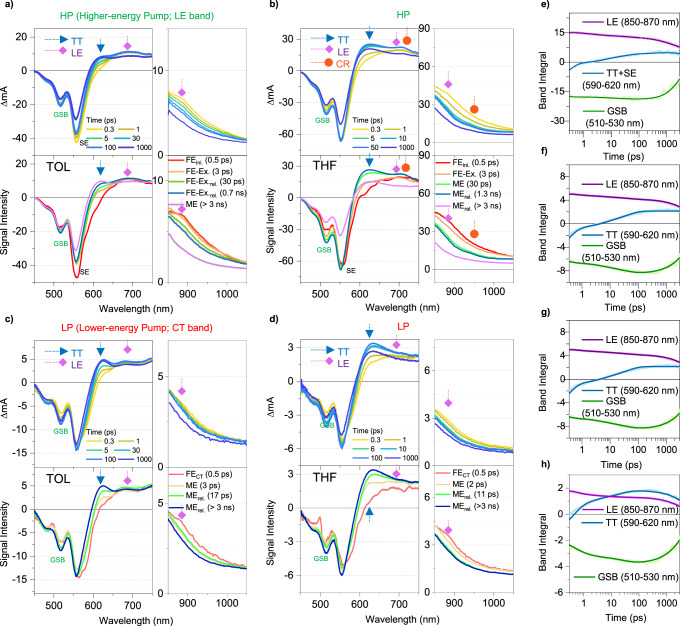
The fs-transient absorption (TA) measurements of Tris-PBI upon photoexcitation at higher energy (HP, 550 nm) and lower energy (LP, 600 nm). **a**, **b** The representative spectra (top panel) and EAS (bottom panel) of **Tris-PBI** in TOL (**a**) and THF (**b**) by HP. The respective arrows indicate the ground state bleach (GSB), locally excited (LE; diamond), charge-resonance (CR; circle), and triplet pair (TT; triangle). **c**, **d** The representative spectra (top panel) and EAS (bottom panel) of **Tris-PBI** in TOL (**c**) and THF (**d**) by LP. The respective arrows indicate the ground state bleach (GSB), locally excited (LE) and triplet pair (TT). **e**, **f** The TA kinetics of **Tris-PBI** in TOL (**e**) and THF (**f**) by HP. **g**, **h** The TA kinetics of **Tris-PBI** in TOL (**g**) and THF (**h**) by LP. The solid lines correspond to the best-fitted curve convoluted with IRF and multiexponential functions. The detailed assignments of distinct species are described in Supplementary Note 2.

**Fig. 4:**
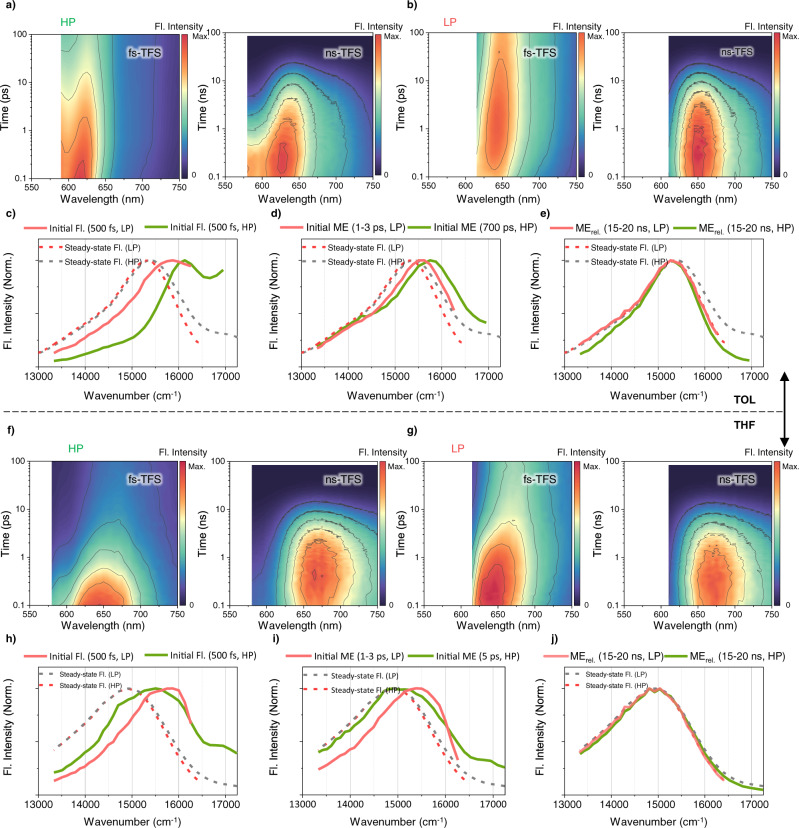
The fs-to-ns transient fluorescence spectra (TFS) of Tris-PBI by HP (550 nm, higher energy pump) and LP (600 nm, lower energy pump). **a**,**b** the *fs*-to-*ns* TFS in TOL. The 2D contour map of TFS in the range of 0.1–100 ps (left) and 0.1–90 ns (right) by HP (**a**) and LP (**b**). The color bar indicates the relative fluorescence intensity. **c**-**e** the spectral comparison between the FE and FE_CT_ states (**c**), the initial ME states (**d**), and the relaxed ME states (**e**) in TOL by HP (green line) and LP (yellow line). The steady-state fluorescence spectra by the HP (grey dashed line) and LP (red dashed line) are overlayed for a comparison. **f**, **g** the *fs*-to-*ns* TFS in THF. The 2D contour map of TFS in the range of 0.1–100 ps (left) and 0.1–90 ns (right) by HP (**f**) and LP (**g**). The color bar indicates the relative fluorescence intensity. **h**–**j** the spectral comparison between the FE and FE_CT_ states (**h**), the initial ME states (**i**), and the relaxed ME states (**j**) in THF by HP (green line) and LP (yellow line). The steady-state fluorescence spectra by HP (grey dashed line) and LP (red dashed line) are overlayed for a comparison.

To scrutinize the excited-state dynamics of **Tris-PBI** by the HP and LP, we carried out *fs*-transient absorption (*fs*-TA) measurements in TOL. Here, we note that the similar TA spectra were observed upon photoexcitation at 490, 530, and 550 nm and there is no pump-fluence dependence (Supplementary Figs. 16, 17). To analyze the spectral evolution, the evolution associated spectra (EAS) are extracted from *glotaran* program^60^. The initial TA spectrum by HP shows the broad excited-state absorption (ESA) bands at 680 and 870 nm, corresponding to the FE state, and distinct stimulated emission (SE) spanning from 550 to 650 nm (Fig. 3a and Supplementary Fig. 17). The TA spectra of locally excited (LE)-dominant species (FE_ini._) not only become broad but also show the distinct decrease in SE with the time constants of 0.5, 3, and 30 ps, which could be assigned to excimer-like state formation (FE-Ex.) and subsequent structural relaxation processes (FE-Ex._rel._). Afterwards, the FE-Ex._rel._ proceeds to the ME state with the time constant of 700 ps, which shows the distinct appearance of the ESA band at 620 nm corresponding to triplet pair (TT) configuration (Fig. 3a, e, and Supplementary Fig. 22)^32,45^. Furthermore, *fs*-to-*ns*-transient fluorescence spectra (TFS) in TOL by HP support our assignments by *fs*-TA (Fig. 4a; green solid lines in Fig. 4c, d). The initial TFS shows a distinct vibronic band (A_0-1_), indicating the FE character (green solid line in Fig. 4c). Next, the TFS show a broadening of the vibronic band in FE_int._ within 1 ps, followed by the subsequent red-shift with the time constants of 3 and 30 ps (Fig. 4a). Subsequently, the evolution with 700 ps could be assigned to the MEG process, which is attributed to the prominent evolution of ME fluorescence (green solid line in Fig. 4d). In this respect, the initial FE state by HP proceeds into the excimer-like manifolds. Albeit such energetic relaxation hinders the ultrafast MEG, still the excimer-like state mediates the MEG. Afterwards, the high-lying ME state relaxes to the lower-lying ME state with the time constant of 3 ns, followed by the relaxation to the ground state with 12 ns (green solid line in Fig. 4e).

Compared to the inefficient MEG process by HP, the direct excitation of the CT band results in the efficient MEG process. As shown in Fig. 3c, the initial state (FE_CT_) populated by LP indicates diminished SE band compared to the FE state by the HP due to the strong interaction between the FE and CT states. Surprisingly, the TA spectra of the FE_CT_ state evolve with a prominent rise of TT band and GSB with the time constants of 0.5, 3, 17 ps, indicating the direct population of the ME state with the time constant of 500 fs and subsequent ME relaxation processes (Fig. 3c, g). In addition to *fs*-TA, the evolution of TFS manifests an efficient MEG process with the time constant of 500 fs, indicating the appearance of ME fluorescence (red solid lines in Fig. 4c, d). Subsequent processes are accompanied by structural relaxation, showing the gradual red-shift of the ME fluorescence. In addition, the red-shifted and broad FE_CT_ fluorescence compared to FE fluorescence support a strong CR configuration (green and red solid lines in Fig. 4c). Therefore, the accelerated MEG process by LP suggests that the FE_CT_ state proceeds to the ME manifolds through the direct coupling rather than the excimer-mediated MEG process. The ME state relaxes towards the ground state with the time constant of 12 ns (red solid line in Fig. 4e).

To investigate the CT-state dependent MEG processes, we carried out fs-TA measurements in polar media (THF and BCN, Fig. 3b, d, f, h, and Supplementary Fig. 19). In polar media, while the MEG process by HP is accelerated to a few picoseconds, the MEG process by LP shows a minor effect on the solvent polarity. The diminished SE in the TA spectrum and broad TFS by HP are attributed to the mixing of the FE state with the stabilized CT state (Fig. 3b and green solid line in Fig. 4h). The initial species evolve into the FE-Ex. state with the time constant of 500 fs, which is in a good agreement with that in TOL. Subsequently, the ME state is generated with accelerated rate depending upon the solvent polarity (3 and 30 ps in THF; 2 and 20 ps in BCN, Fig. 3b and Supplementary Figs. 26–29). Furthermore, the ME state not only manifests the co-existence of broad LE, TT, and CR bands (PBI anion at 720 and 960 nm and PBI cation at 600 nm) in the TA spectra^38^ but also shows very broad ME fluorescence (Fig. 3b, i). These results suggest that the stabilization of CT state leads to accelerated MEG process like a real CT-assisted mechanism^7,61^. Considering the excimer state is defined by an admixture of FE and CT diabats, it is reasonable that the excimer intermediate by HP proceeds to the ME state through the excimer-mediated mechanism. It is remarkable, however, that the evolution of **Tris-PBI** spectra by the LP is in stark contrast to that by the HP: (1) The MEG process is less sensitive to the solvent-polarity (Fig. 3h and Supplementary Fig. 19). (2) The minor CR configuration is observed in only highly polar solvent BCN even though the solvation processes stabilize the ME state. (3) The MEG process is efficient in all solvents. In this regard, it is evident that the direct coupling between FE_CT_ and ME states contributes to the MEG by the LP (CT-mediated mechanism) due to large CT contribution of the red-edge band Finally, the ME state lifetime by both HP and LP decreases with the solvent polarity, showing the multiconfiguration nature of the ME state.

### Insights into structural dynamics for the MEG process through time-domain Raman spectroscopy

Since the structural dynamics is associated with the excited-state relaxation pathways (see Supplementary Note 1), understanding the structural dynamics is important for the MEG process^62,63^. In this respect, we have utilized time-resolved impulsive stimulated Raman spectroscopy (TR-ISRS) to investigate how the molecular structure evolves during the MEG process.

**Fig. 5:**
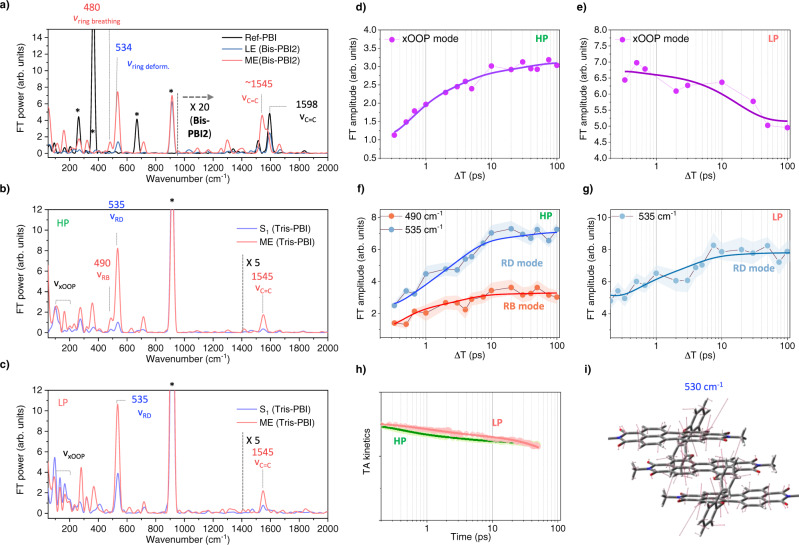
Excited-state vibrational spectra of Tris-PBI in THF by the HP (550 nm, higher energy pump) and LP (600 nm, lower energy pump). **a** The excited state Raman spectra of **Ref-PBI** and **Bis-PBI2** in CHCl_3_ and THF, respectively, corresponding to the monomer-counter unit (black line), Frenkel exciton (pale blue line), ME (yellow line) states (The details are described in the Supplementary Note 4). The key vibrational modes are marked. The relative FT power of **Bis-PBI2** is rescaled for clarity. The raw excited-state Raman spectra of **Bis-PBI2** are shown in Supplementary Fig. 33. **b**, **c** The excited-state Raman spectra of **Tris-PBI** for the initial S_1_ (∆T = 300 fs) and ME (∆T = 30 ps) states in THF by the HP (**b**) and LP (**c**). The FT power of peaks above 1400 cm^−1^ is multiplied by 5 to make the high-frequency modes more clearly visible. Asterisks indicate solvent Raman bands. **d**–**g** FT amplitude kinetics of the xOOP modes (purple dot), the RD mode (blue dot), and the RB mode (red dot) by the HP (**d**, **f**) and LP (**e**, **g**). The RD and RB modes are fitted by the Gaussian function. The FT amplitude of xOOP mode is calculated by the band integral. The solid lines correspond to the best-fitted curve convolved with IRF and multiexponential functions. The shade line indicates the error bar that is defined by standard deviation. **h** The TA kinetics by the HP (green line) and LP (red line). The solid lines correspond to the best-fitted curve convolved with IRF and multiexponential functions. **i** Displacement vectors of the RD mode (530 cm^−1^, a scaling factor 0.953 is applied).

Figure 5a indicates the representative vibrational modes of **Ref-PBI** and **Bis-PBI2** showing the FE (1595 cm^−1^) and ME_LE+CR+TT_ (480 and 530 cm^−1^) states (see Supplementary Note 4). Based on these results, we discuss the excited-state Raman spectra of **Tris-PBI** in THF (Fig. 5). By HP, the initial Raman spectrum shows prominent modes at 130, 180, 490, and 535 cm^−1^ in the low-frequency region (Fig. 5b). The modes in the frequency region below 200 cm^−1^ perturb the intermolecular distance, which could be assigned to the interchromophoric out-of-plane (xOOP) mode. And the other modes are assigned to ring breathing (RB, 490 cm^−1^) and ring deformation (RD, 535 cm^−1^) modes based on the DFT calculations (Supplementary Figs. 39, 40). During the MEG, the xOOP, RB, and RD modes show a significant increase in the FT amplitude. Surprisingly, the FT amplitude of these vibrational modes rises with the evolution of ME state against the decrease in TA kinetics (Fig. 5d, f, h), presumably suggesting that these vibrational modes are associated with the nuclear motion along the reaction coordinates. In high-frequency region, while the mode at 1595 cm^−1^ (C=C stretch mode for the LE state) is not observed, the mode at 1545 cm^−1^ (C=C stretch mode for the CR state) becomes prominent with the MEG process, which supports a CR configuration in ME state. In contrast, in weak polar TOL, the xOOP mode shows a rise of FT amplitude against the TA kinetics, but there is no signal at 480 and 535 cm^−1^ (Supplementary Fig. 35). These results manifest the following hypotheses: (1) Since the xOOP mode plays a decisive role in the excimer formation^64,65^, the rise of xOOP mode shows the excimer-mediated MEG process by HP. (2) Considering the rate constants of MEG process depending upon the solvent-polarity, an absence of RB and RD modes in TOL suggests that these vibrational modes facilitate a mixing between the CT and LE/ME states. In addition to results by HP, the excited-state Raman spectra by LP shows a significant role of the RD mode during the MEG process (Fig. 5c and Supplementary Fig. 36). The prominent appearance of RD mode at the initial state are distinct, which may be attributed to a strong CR character (Fig. 5g, h). Furthermore, an increase in the FT amplitude of the RD mode against the TA kinetics in both TOL and THF supports that the RD mode promotes the efficient MEG process. Finally, the xOOP modes by LP simply follows the TA kinetics, suggesting that xOOP mode is a spectator mode (Fig. 5e).

### RAS-3SF simulation

**Fig. 6:**
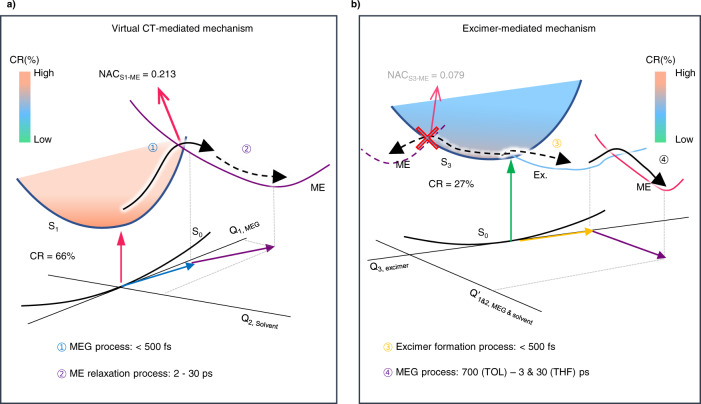
Schematic illustrations of multiexciton generation pathways by the HP and LP. The (virtual) CT-mediated mechanism by LP (**a**) and the excimer-mediated (or real CT-mediated) mechanism by HP (**b**). The NAC_Sn-ME_ and CR configurations in the S_n_ states are estimated by RAS-3SF simulations (see Supplementary Table 7). The color bar indicates the relative CR configuration in the respective states. Here, we suggest that the respective reaction coordinates (Q_1_, Q_2_, and Q_3_) presumably correspond to the vibrational modes based on the excited-state Raman in Fig. 5. (Q_1_: high-frequency mode, Q_2_: RD and RB modes, and Q_3_: xOOP mode).

To attain further insight into the MEG mechanism, it is crucial to understand the electronic characters of the excited states and coupling strengths between MEG-relevant states. Despite the success of TDDFT for the description of exciton coupling of trimeric array, it is impossible for TD-DFT to access the doubly (or higher) excited electronic configurations such as triplet pair state since the TDDFT with the adiabatic approximations is restricted to single excitations. In this regard, we performed restricted active space with spin-flip (RAS-SF) simulation, which has been widely used to interrogate the MEG process. RAS-3SF calculations for **Tris-PBI** suggest that the S_1_ state exhibits 66% of CR character, consistent with the experimental observation upon LP into the CT band (Table S7)^66,67^. This feature is indeed quite different from the null-type PBI dimers studied in our previous work^45^ that features the LE as the lowest transition. The pairwise CR contribution analysis shows that CT mainly occurs from the central PBI unit to the two outer moieties, which contributes to CR state stabilization. The second allowed excitation (S_3_) with the highest oscillator strength is energetically ~0.1 eV above S_1_, which well reproduces the small energy difference between the band edge used for LP and the absorption maximum used for HP. It is noted that S_3_ has much lower contribution from CR diabats compared to S_1_ (Fig. 6). In addition to adiabatic wavefunction decomposition, we estimated nonadiabatic coupling (NAC) to understand the physical origin that accelerates the MEG upon LP. The square of one-particle transition density matrix (|γ|) between S_1_ and ME state is 0.38, which is 1.8 times larger than that between S_3_ and ME state (0.21). With small energy difference between S_1_ and S_3_, this results in NAC_S1-ME_ (0.213) which is about 2.7 times of NAC_S3-ME_.(0.079) Such enhanced coupling can be obtained by the increased contribution of CR diabat in S_1_ (66%) compared to that in S_3_ (27%). To summarize the RAS-3SF result (Fig. 6), higher contribution of CR diabat in S_1_ can explain more efficient coupling between S_1_ and ME than that between S_3_ and ME^45^. Considering both the large CR contributions in the S_1_ state and ultrafast MEG process, we suggest that the structural feature of **Tris-PBI** is ideal for the MEG process, and thereby the MEG mechanism is a (virtual) CT-mediated mechanism. In contrast, the weak coupling between S_3_ and ME is the rationale why the photoexcitation at higher energy leads to the excimer-mediated mechanism, which has been a dispute in endothermic SF materials^29,30,67^. Recently, Bae et al. has suggested that there is the lower limit of CR configuration in S_1_ state to proceed to the excimer state^68^. In this regard, as for **Tris-PBI**, the only moderate CR configuration in the S_3_ state leads to the energetic downhill relaxation into the excimer (dimeric) state that requires some activation energy to proceed into the ME state, thereby reducing the rate for MEG. However, by tuning the solvent polarity, the CT-enhanced excimer state can accelerate the MEG process as a real CT-mediated mechanism (Fig. 6).

In conclusion, we have discovered an excitation-wavelength dependent MEG mechanism at the molecular level, which originates from strong interchromophoric interactions within the triple-chromophore ensemble of **Tris-PBI**. Thus, with this model compound we could elucidate the comprehensive effects of delocalization, CT coupling, and excimer trap states on the MEG process that have been previously discussed for solid state materials^11,15,51,55^. By variation of the excitation wavelengths, we could directly influence the excited state dynamics. Excitation of **Tris-PBI** into its main absorption band afforded an excimer-like state within ultrafast timescale that slowed down and reduced the efficiency of the MEG process in solvents of low polarity whilst tuning of the CT energy via more polar solvents mediated the more efficient MEG process via the excimer-mediated (real CT-mediated) mechanism. In contrast, upon excitation into the red band edge, a state with significant CT interaction between the central and the outer chromophores was populated that gave rise to the ultrafast MEG even in low polarity solvents. With these results, we revealed structural prerequisites by the **Tris-PBI** model compound that are ideal for MEG processes in PBI thin films. More generally, our findings suggest that null-type aggregates with strong CT couplings should afford the highest SF efficiencies. Thus, our study provides a guideline for the molecular design of efficient SF devices.

## Methods

### Sample preparation and Single crystal X-ray diffraction

The synthesis and analysis of single-crystal X-ray diffraction of **Tris-PBI** were described in the Supplementary Synthesis and Compound Characterization and Supplementary Methods.

### Steady- state measurements

Steady-state absorption spectra were measured on a UV/Vis/NIR spectrometer (Varian, Cary5000) and fluorescence spectra were measured on a fluorescence spectrophotometer (Hitachi, F-7000). Fluorescence spectra are spectrally corrected by using correction factor of the fluorescence spectrophotometer. HPLC-grade solvents were purchased from Sigma-Aldrich and used without further purification.

### Transient absorption spectroscopy

The transient absorption (TA) spectroscopy setup has been described in Supplementary Methods. In brief, a Ti:sapphire regenerative amplifier (Integra-C, Quantronix, 800 nm, 1 mJ, 1 kHz, 100 fs,) was used as a fundamental laser source of femtosecond transient absorption spectrometer. White light continuum (WLC) probe pulses were generated using a sapphire window (4 mm thick, c-axis cut, Eksma optics) by focusing a small portion of the transmitted fundamental pulses. Pump pulses (550 and 600 nm) were generated through a commercial collinear optical parametric amplifier (Palitra, Quantronix). The pulse energy of the pump was attenuated to 300 nJ and its polarization was set at the magic angle to the vertically polarized probe by using a half-wave plate (Thorlabs) and a Glan-laser polarizer (Thorlabs). A 2 mm path length quartz cell (21/Q/2, Starna) was used and the optical density (OD) of the sample was about 0.5. The TA spectra were measured in a shot-to-shot fashion by modulating pump pulses at 500 Hz using an optical chopper (MC1F10, Thorlabs). With the optical Kerr signal measurements by *n*-hexane, cross-correlation FWHM (full-width at half-maximum) in the TA experiments was estimated to be about 200–300 fs depending on the probe wavelength and the chirp of WLC probe pulses was measured to be 1.2 ps in the 450–1300 nm region.

### Time-resolved fluorescence upconversion spectroscopy

The details of time-resolved fluorescence upcoversion spectroscopy (TF) setup have been described in Supplementary Methods. Briefly, pump pulses at 550 nm (HP) and 595 nm (LP) were generated by the second harmonic generation (SHG) in a 100 μm thick beta-barium borate (BBO) crystal, and the residual fundamental laser pulses were used as gate pulses. SFG of the fluorescence and the gate pulse was carried out by using a 100 μm thick BBO crystal. The instrument response functions (IRF) estimated by cross-correlation between the scattered pump pulse and the gate were ~110 and ~200 fs full width at half-maximum (FWHM) for HP and LP, respectively. All TF measurements were performed at the magic angle configuration. For TF spectra measurements, the phase matching angle of the BBO crystal for SFG and monochromator were controlled simultaneously.

### Time-resolved impulsive stimulated Raman spectroscopy

The details of time-resolved impulsive stimulated Raman spectroscopy (TR-ISRS) setup have been described in Supplementary Methods. Briefly, a Yb:KGW regenerative amplifier (PHAROS-SP-1.5mJ, Light Conversion, 1030 nm, 600 μJ, 10 kHz, 176 fs) was used as the main source for TR-ISRS. Actinic pump (P_1_, 550 and 595 nm, ~170 fs) is generated by a commercial collinear optical parametric amplifier (ORPHEUS, Light Conversion) combined with a second-harmonic generation stage (LYRA-SH, Light Conversion). A home-built noncollinear optical parametric amplifier generates broadband pulses covering the near-infrared region (700–900 nm, compressed to sub-10 fs by chirped mirrors and wedges) and they were used as Raman pump (P_2_) and probe (P_3_) pulses after dividing by a beam splitter (Venteon). At the sample position, the energies (and 1/e^2^ beam diameters) of the P_1_, P_2_, and P_3_ pulses were 200 nJ (140 μm), 90 nJ (110 μm), and 3 nJ (100 μm), respectively, and all pulses were horizontally polarized. A 500 μm optical path length flow cell with ultrathin wall apertures (48/UTWA2/Q/0.5, Starna) was used and the 2.5 ml sample solution (OD for a 500 μm cell = 0.8 at absorption maximum) was flowed by a micro annular gear pump (mzr-4622 M2.1, HNP Mikrosysteme). The P_3_ and the reference pulses were detected using the Si photodiodes (S2281-04, Hamamatsu) without any filters for open-band detection to minimize the contribution of vibrational coherences from the ground-state and solvent molecules. The P_2_ pulse is modulated at 5 kHz by a mechanical chopper (MC1F60, Thorlabs), which allows data processing in a shot-to-shot fashion. For TA experiments with P_1_ and P_3_ pulses (data in Fig. 5h), The P_2_ pulse is blocked by a beam block and the P_1_ pulse is modulated at 5 kHz by a mechenical chopper (MC1F60, Thorlabs) to get P_1_-induced TA signals.

## Supplementary information

Supplementary Information

## Data Availability

Crystallographic data for the structure reported in this Article have been deposited at the Cambridge Crystallographic Data Centre, under deposition number CCDC 2124115 (**Tris-PBI**). Copies of the data can be obtained free of charge via https://www.ccdc.cam.ac.uk/structures/. All the data that support the findings of this study are available in this article and its Supplementary Information. The raw data are available from the corresponding authors upon request.
