# Supplementary material for: Steering the multiexciton generation in slip-stacked perylene dye array via exciton coupling
Source: Nat Commun. 2022 Aug 2;13:4488. doi: 10.1038/s41467-022-31958-1 (PMC9345863; doi:10.1038/s41467-022-31958-1)
Supplement: Supplementary file 1 — Supplementary Information [file 41467_2022_31958_MOESM1_ESM.pdf]

Supplementary Information

Steering the Multiexciton Generation in Slip-Stacked Perylene Dye Array via Exciton Coupling

Yongseok Hong,[1] Maximilian Rudolf,[2] Munnyon Kim,[3] Juno Kim,[1] Tim Schembri,[4] Ana-Maria Krause,[4] Kazutaka Shoyama,[4] David Bialas,[2] Merle I. S. Röhr,[4*] Taiha Joo,[3,*] Hyungjun Kim,[5,*] Dongho Kim,[1,6,*] and Frank Würthner,[2,4,*]

[1]Spectroscopy Laboratory for Functional π-Electronic Systems and Department of Chemistry, Yonsei University, Seoul 03722, Republic of Korea

[2]Universitat Würzburg, Institut für Organische Chemie, Am Hubland, 97074 Würzburg, Germany

[3]Department of Chemistry, Pohang University of Science and Technology (POSTECH), Pohang, 37673, Republic of Korea

[4]Universität Würzburg, Center for Nanosystems Chemistry, Theodor-Boveri Weg, 97074 Würzburg, Germany

[5]Department of Chemistry and Research Institute of Basic Sciences, Incheon National University, Incheon 22012, Republic of Korea

[6]Division of Energy Materials, Pohang University of Science and Technology (POSTECH), Pohang, 37673, Republic of Korea

*Correspondence: merle.roehr@uni-wuerzburg.de (M.R.), thjoo@postech.ac.kr (T.J.), kim.hyungjun@inu.ac.kr (H.K.), dongho@yonsei.ac.kr (D.K.), and wuerthner@uni-wuerzburg.de (F.W.)

Table of the contents

1. Supplementary Methods
2. Synthesis and Compound Characterization
3. Single crystal X-ray Structure Analysis
4. Steady-State Absorption and Fluorescence Spectroscopy
5. Time-Resolved Electronic Spectroscopy
6. Time-Resolved vibrational Spectroscopy
7. Quantum Simulations (TD-DFT and RAS-3SF)
8. Supplementary References

Supplementary Methods

All commercial chemicals and reagents, unless otherwise stated, were used without further purification. Column chromatography was performed on silica-gel (particle size 0.040–0.063 mm) with freshly distilled solvents as eluents. Recycling gel permeation chromatography (GPC) of the target compound was performed on a Shimadzu syste, (LC-20AD pump, SPD-MA20A detector) with HPLC grade chloroform as solvent. Melting points were acquired with an Olympus BX41 polarization microscope and are uncorrected. 1H and 13C NMR spectroscopy was performed with a Bruker Avance III HD 400 MHz or Bruker Avance III HD 600 MHz spectrometer. 13C NMR spectra are broad band proton decoupled. The chemical shifts are reported in parts per million (ppm), reported relative to tetramethylsilane and referenced internally to the residual proton solvent resonances or natural abundance carbon resonances. The coupling constants (J) are listed in Hertz (Hz). MALDI-TOF mass spectrometry was performed with a Burker Daltonics ultrafleXtreme mass spectrometer. ESI-TOF mass spectra were recorded on a Bruker Daltonics microTOF focus spectrometer. UV–vis absorption and fluorescence measurements were conducted with spectroscopic grade solvents.

Single crystal X-ray diffraction

Single crystal X-ray diffraction data were collected at the P11 beamline at DESY. The diffraction data were collected by a single 360° ϕ scan at 100 K. The diffraction data were indexed, integrated, and scaled using the XDS program package.[S1] The structures were solved using SHELXT,[S2] expanded with Fourier techniques and refined using the SHELX software package.[S3] Hydrogen atoms were assigned at idealized positions and were included in the calculation of structure factors. All non-hydrogen atoms in the main residue were refined anisotropically. The diffraction data had a resolution of ca. 0.90 Å (defined by the resolution shell with intensity $I > 2\sigma$). For refinement diffraction data until 0.66 Å were used with relatively lower completeness between 0.78 and 0.66 Å. In the crystal structure all the alkyl substituents at the imide units had heavy disorder and accordingly had to be modelled with rather strong constraints and restraints using standard SHELX commands RIGU, DELU, ISOR, SADI, DFIX, DANG, and SIMU. The methanol molecules in the solvent accessible voids also had disorder and their bond length was fixed to 1.413 Å using the AFIX 6 instructions. The final refinement step was performed with a damping factor of 100 using the DAMP instruction of SHELX. This is due to weak outer-shell diffractions which caused instability in the refinement of heavily disordered alkyl side chains and solvent molecules. This is not surprising because the molecule contains six chirality centres. Thus, all combinations of diastereomers and enantiomers are given that were accounted for by disorder analysis.

The crystal structure had a tetragonal crystal system and was solved as the $I4_1$ space group. A pseudo-inversion centre was found in the middle of PBI trimer, which made the space group look like $I4_1/a$. However, disorder analysis indicated that the side-chains cannot be well modelled with an inversion centre at the middle of the molecule and that the crystal structure should be better described as $I4_1$ than $I4_1/a$. As an additional effort to substantiate the choice of space group, we have also solved the structure with all possible space groups ($I\bar{4}$, $I4_1$, and $I4_1/a$) by replacing all 2-hexyldecyl chains with methyl group and using the SQUEEZE routine (Table S3). The comparison of three models showed significantly elevated R_1 value for $I4_1/a$ (0.2118) than for the other two, and nearly equivalent R_1 values for $I4_1$ and $I\bar{4}$ with that of $I4_1$ slightly lower (R = 0.1376 for $I4_1$, 0.1446 for $I\bar{4}$,). Other statistics such as number of outliners or K values also show better fit for $I4_1$ or $I\bar{4}$ than for $I4_1/a$. These results indicate that the symmetry of the core structure of PBI trimer is deviated from the expected C_i for the $I4_1/a$ space group and is actually C_1. Therefore, we concluded that the space group of this crystal structure should be either $I4_1$ or $I\bar{4}$. The difference between $I4_1$ and $I\bar{4}$ is whether the neighboring stacks of trimers have equal or alternating chirality. Heavy disorder of the side chains prevents strong interaction between the neighboring stacks. Therefore, it is assumed that the real structure is rather a mixture of both cases, in some parts of the crystal the former (packing of $I4_1$) is present and in other parts the latter (packing of $I\bar{4}$) exists. The statistics indicates that the former prevails the latter and we decided to solve the structure using this $I4_1$ space group.

Verification of the crystallographic data by the checkcif routine caused an Alert B, which is due to the use of the DAMP instruction of SHELX. This command was used to stabilize the refinement of heavily disordered 2-hexyldecyl side chains and methanol molecules in the solvent accessible voids. In order to substantiate the use of damping for the crystal structure of **Tris-PBI** we have also performed analysis of the data by removing all methanol molecules and using SQUEEZE. Analysis in this way, however, did not lead to stable refinement, on the contrary, lead to necessity to use a higher damping factor (larger than 1000) than the one we used for the final CIF (100). We have also tested refinement by omitting side chains that are not stable or weakly seen in the difference maps, however, this treatment did not lead to stable refinement either. Furthermore, we have tried to analyze the data by omitting all side-chains except the methylene bridges just next to the imide nitrogen atom. This analysis caused oddities in the ellipsoids of the core structure which were evident from many errors of Hirschfeld analysis by the checkcif routine. Additionally, the statistic factors (e.g. *R*-factors) of this model were significantly worse than the model created without SQUEEZE (*R*-factor: 0.1376 with SQUEEZE, 0.0870 without SQUEEZE). We attribute these issues to model bias generated by SQUEEZE. Therefore, we concluded that SQUEEZE is not a suitable solution for avoiding the strong restraints and damping for our crystallographic data, and it rather deteriorates the diffraction data by adding model bias in our case.

The diffraction resolution of the crystallographic data of **Tris-PBI** was, according to the typical definition of the resolution shell with signal-to-noise ratio larger than 2, until 0.90 Å. Nevertheless, we have used all the diffraction data measured down to a minimum of 0.66 Å with rather low completeness between 0.84 and 0.66 Å due to following reasons. First, it has been proposed that inclusion of diffraction data lower than the resolution limit may make some improvements and does not deteriorate the model.[S4]. Second, we find that the refinement of such large crystal structure that includes heavy disorder both in the main residues and the solvent accessible voids are more stable with inclusion of high resolution diffraction data below typical resolution limit.[S5]

Steady-state UV/Vis/NIR and fluorescence measurements.

Steady-state absorption spectra were measured on a UV/Vis/NIR spectrometer (Varian, Cary5000) and fluorescence spectra were measured on a fluorescence spectrophotometer (Hitachi, F-7000). Fluorescence spectra are spectrally corrected by using correction factor of the fluorescence spectrophotometer. HPLC-grade solvents were purchased from Sigma-Aldrich and used without further purification. Fluorescence quantum yields were determined with the relative method (A < 0.05) and using *N*,*N*′-bis[2,6-di-isopropylphenyl]perylene-3,4:9,10-bis(dicarboximide) (Φ_{fl} = 100% in chloroform) as reference.[S6]

Time-resolved absorption spectroscopy (fs-TA)

The transient absorption (TA) spectroscopy setup has been described in detail elsewhere.[S7,S8] In brief, a Ti:sapphire regenerative amplifier (Integra-C, Quantronix, 800 nm, 1 mJ, 1 kHz, 100 fs,) was used as a fundamental laser source of femtosecond transient absorption spectrometer. White light continuum (WLC) probe pulses were generated using Sapphire window (3mm thick, for visible region) and YAG window (4 mm thick, for NIR region) by focusing a small portion of the transmitted fundamental pulses. Pump pulses (510 nm) were generated through a commercial collinear optical parametric amplifier (Palitra, Quantronix). The pulse energy of the pump was attenuated to 300 nJ and its polarization was set at the magic angle to the vertically polarized probe by using a half-wave plate (Thorlabs) and a Glan-laser polarizer (Thorlabs). A 2 mm path length quartz cell (21/Q/2, Starna) was used and the optical density (OD) of the sample was about 0.5. The TA spectra were measured in a shot-to-shot fashion by modulating pump pulses at 500 Hz using an optical chopper (MC1F10, Thorlabs). With the optical Kerr signal measurements by n-hexane, cross-correlation FWHM (full-width at half-maximum) in the TA experiments was estimated to be about 200-300 fs depending on the probe wavelength and the chirp of WLC probe pulses was measured to be 1.2 ps in the 450-1350 nm region.

Nanosecond transient absorption measurements

The nanosecond transient absorption spectra were obtained using nanosecond flash photolysis techniques. Specifically, a tunable excitation pulse was generated using an Optical Parametric Oscillator system (Continuum, Surelite OPO), which was pumped by 355 nm from the third-harmonic output of a Q-switched Nd:YAG laser (Continuum, Surelite II-10). The time duration of the excitation pulse was ca. 6 ns, and the 11 pulse energy was ca. 2 mJ/pulse. A CW Xe lamp (150 W) was used as the probe light source for the transient absorption measurement. The probe light was collimated on the sample cell and was spectrally resolved using a 15 cm monochromator (Acton Research, SP150) equipped with a 600 grooves/mm grating after passing the sample. The spectral resolution was approximately 3 nm for the transient absorption experiment. The light signal was detected using an avalanche photodiode (APD). The output signal from the APD was recorded using a 500 MHz digital storage oscilloscope (Lecroy, WaveRunner 6050A) for the temporal profile measurement. Since the triplet-state dynamics of molecules in solution are strongly dependent on the concentration of oxygen molecules dissolved in solution, we attempted to remove oxygen by degassing with Ar gas for 1 hour.

Time-resolved fluorescence upconversion spectroscopy (fs-TF)

The details of time-resolved fluorescence upcoversion spectroscopy (TF) setup has been described in elsewhere.[S9,S10,S11] Briefly, pump pulses at 550 nm (HP) and 595 nm (LP) were generated by the second harmonic generation (SHG) in a 100 mm thick beta-barium borate (BBO) crystal, and the residual fundamental laser pulses were used as gate pulses. SFG of the fluorescence and the gate pulse was carried out by using a 100 mm thick BBO crystal. The instrument response functions (IRF) estimated by cross-correlation between the scattered pump pulse and the gate were 110 and 180 fs full width at half-maximum (FWHM) for HP and LP, respectively. All TF measurements were performed at the magic angle configuration. For TF spectra measurements, the phase matching angle of the BBO crystal for SFG and monochromator were controlled simultaneously.

ns-Time-resolved fluorescence upconversion spectroscopy experiment (TCSPC)

A time-correlated single-photon-counting (TCSPC) system was used for measurements of spontaneous fluorescence decay. Pump pulses (500 kHz) at 550 nm (HP) and 595 nm (LP) were generated by the second harmonic generation (SHG) in a 100 mm thick beta-barium borate (BBO) crystal. Its polarization is set to the magic angle (54.7 degree). The fluorescence was collected by a microchannel plate photomultiplier (SP-300i, Acton Research) connected to a hybrid photo-detector (HPM100-07, Becker and Hickl GmbH). The instrument response functions (IRF) was estimated to 110 ps full width at half-maximum (FWHM).

Time-Resolved Impulsive Stimulated Raman Spectroscopy (TR-ISRS).

A schematic diagram of the TR-ISRS is shown in Supplementary Figure S28. We built and modified the setup by referring to papers from Tahara, Kukura, Brida, and Nelson groups.[S12,S13,S14,S15] The details were described elsewhere.[S16] Briefly, a Yb:KGW regenerative amplifier (PHAROS-SP-1.5mJ, Light Conversion, 1030 nm, 600 µJ, 10 kHz, 176 fs) was used as the main source for TR-ISRS. Actinic pump ($P_{1,\,HP}$ and $P_{2\,LP}$, 550 and 595 nm, ~ 170 fs) is generated by a commercial collinear optical parametric amplifier (ORPHEUS, Light Conversion) combined with a second-harmonic generation stage (LYRA-SH, Light Conversion). A home-built noncollinear optical parametric amplifier generates broadband pulses covering the near-infrared region (700-900 nm, compressed to sub-10 fs by chirped mirrors and wedges) and they were used as Raman pump (P_2) and probe (P_3) pulses after dividing by a beam splitter (Venteon). At the sample position, the energies (and $1/e^2$ beam diameters) of the P_1, P_2, and P_3 pulses were 250 nJ (180 µm), 120 nJ (120 µm), and 3 nJ (100 µm), respectively, and all pulses were horizontally polarized. A 500 µm optical path length flow cell with ultrathin wall apertures (48/UTWA2/Q/0.5, Starna) was used and the 2.5 ml sample solution (OD for a 500 µm cell = 0.8 at absorption maximum) was flowed by a micro annular gear pump (mzr-4622 M2.1, HNP Mikrosysteme). The P_3 and the reference pulses were detected using the Si photodiodes (S2281-04, Hamamatsu) without any filters for open-band detection to minimize the

contribution of vibrational coherences from the ground-state and solvent molecules. The P_2 pulse is modulated at 5 kHz by a mechanical chopper (MC1F60, Thorlabs), which allows data processing in a shot-to-shot fashion.

Quantum Chemical Calculations

The quantum chemical calculations for determining the different types of coupling were conducted analogously to those previously reported for **Bis-PBI2**.[S17, S18] We utilized the structure found in the single crystal, replaced the 2-hexyldecyl chains by methyl groups, and performed a structural optimization using the Gaussian 09 program package[S19] with the long-range corrected hybrid density functional ωB97X-D[S20] and a def2-SVP basis set.[S21]. The long-range Coulomb coupling J_{Coul} was calculated using time-dependent density functional theory (TD-DFT). The resulting transition density was projected onto atomic transition charges by a Mulliken style electron excitation analysis for the first excited state using the Multiwfn software package[S22] and J_{Coul} finally calculated using the transition charge method.[S23] The short-range charge-transfer coupling J_{CT} was calculated at the perturbative limit[S24] with the effective electron and hole transfer integrals t_{e} and t_{h}, respectively, determined using the Amsterdam Density Functional program[S25] with the PW91 functional[S26] and a TZP basis set.[S27] The resulting total coupling J_{total} was finally calculated as the sum of J_{Coul} and J_{CT}, and also determined from the UV-Vis absorption spectrum ($J_{\mathrm{total;UV\text{-}Vis}}$) in the perturbative limit[S28] using the same procedure as for **Bis-PBI2**.

The analysis of the electronically excited states can be performed by utilizing either natural transition orbitals (NTO)[S29] or by means of the reduced first-order spinless transition density matrix (TDM) between a ground and an excited electronic state. In case of TD-DFT, the TDM (1) in the atomic orbital basis can be formulated in terms of the excitation, $C^{I}_{i\rightarrow a}$, and de-excitation coefficients, $C^{I}_{i\leftarrow a}$:

$$P^{[\mathrm{AO}]}_{\mu\nu} = 2\sum_{i}\sum_{a} C^{I}_{i\rightarrow a} c_{\mu i} c_{\nu a} + 2\sum_{i}\sum_{a} C^{I}_{i\leftarrow a} c_{\mu i} c_{\nu a} \quad (1)$$

Here, $c_{\mu i}$, are the molecular orbital (MO) coefficients for an atomic orbital μ and a molecular orbital i. Furthermore, the matrix can be contracted to molecular fragments to quantify how the excitation is distributed within an aggregate.[S29,S30,S31] Different formulae exist for this contraction that are compared in a recent work by Titov,[S32] where it is shown that a Löwdin population analysis approach yields the most reliable results. Therefore, we used the following formula (2) to calculate the "fraction of transition density matrix "(FTDM) with a dimension of 3x3:

$$^{5}F_{XY} = \frac{\sum_{\mu\in X}\sum_{\nu\in Y}\left(\mathbf{S}^{1/2}\mathbf{P}^{[\mathrm{AO}]}\mathbf{S}^{1/2}\right)^{2}_{\mu\nu}}{\sum_{\mu\in \textbf{Tris-PBI}}\sum_{\nu\in \textbf{Tris-PBI}}\left(\mathbf{S}^{1/2}\mathbf{P}^{[\mathrm{AO}]}\mathbf{S}^{1/2}\right)^{2}_{\mu\nu}} \quad (2)$$

Here **S** denotes the overlap matrix in atomic orbital basis and *X* and *Y* the molecular fragments. The sum of all FTDM elements is normalized to one, to be able to express the values in percent.

RAS-3SF calculation.

We performed quantum chemical simulations to understand the physical origin of different singlet exciton fission efficiency for two pathways considered in this study. The ground state structure of **Tris-PBI** was taken from the X-ray crystallography, and reoptimized using CAM-B3LYP/6-31G(d,p). Restricted active space method with double spin-flip (RAS-2SF) is widely used to investigate the singlet exciton fission, but this method is only applicable to dimeric systems. Our target molecule consists of three PBI moieties, therefore, it is natural to extend double spin-flip to triple spin-flip (3SF). We performed RAS-3SF/6-31G(d) calculations on the optimized **Tris-PBI** S_0 geometry and obtained raw RAS-3SF energies based on the septet reference state with six unpaired electrons occupying six orbitals. Also, the wavefunction decomposition was conducted to characterize adiabatic wavefunction with four diabats, ground state (GS), local exciton (LE), multi exciton (ME), and charge resonance

(CR). It is well known that the lack of electron dynamic correlation in RAS-SF calculations results in overestimated excitation energies. Electron dynamic correlation can be partially recovered by the comparison of RAS-SF diabatic state energy with the DFT energies. For the **Tris-PBI** S0 state, the singlet excited states up to S9 show strong mixing of diabatic states, and it is hard to correlate such adiabatic state with one specific electronic transition character such as LE, ME, or CR. Due to this difficulty, it is hard to improve raw RAS energies with DFT calculations. Despite such limitation, we can still analyze the physical origin that accelerates the multiexciton generation pathway of LP using the wavefunction composition results and nonadiabatic coupling value (NAC). NAC can be estimated by the norm of one-particle transition density matrix (γ) and energy difference of raw RAS-SF energies (ΔE): $\gamma \Delta E2.$

Supplementary Synthesis and compound characterization

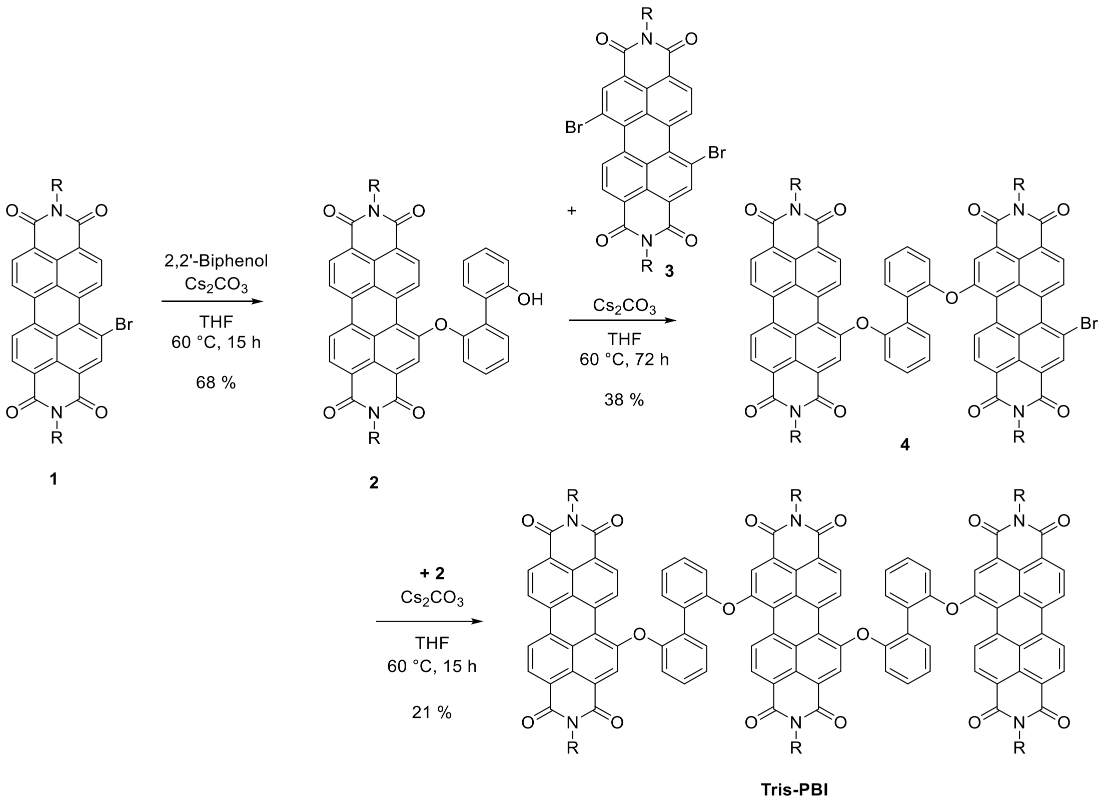

Supplementary Figure 1. Synthesis of **Tris-PBI**. R = *rac*-2-hexyldecyl

Synthesis of 2,2′-biphenol-functionalized PBI 2: Bay-monobrominated PBI **1**[S17] (256 mg, 279 µmol), Cs_2CO_3 (408 mg, 1.25 mmol) and 2,2′-biphenol (519 mg, 2.79 mmol) were dissolved in THF (8 mL). The reaction mixture was stirred at 60 °C for 15 h. The mixture was allowed to cool down to room temperature, water (10 ml) was added and the resulting mixture extracted with dichloromethane (5 × 10 mL). The combined organic phases were washed with water (3 × 10 mL) and dried over $MgSO_4$. The crude product was purified by column chromatography on silica gel (gradient dichloromethane:*n*-hexane 6:4 to dichloromethane) to give compound **2** as a red solid (193 mg, 68 %). Mp.: 209-211 °C. ^{1}H NMR (400 MHz, $CDCl_3$): δ= 9.19 (d, J = 8.2 Hz, 1H), 8.38 (m, 2H), 8.31 (d, J = 8.0 Hz, 1H), 8.10 (m, 3H), 7.53 (m, 1H), 7.35 (m, 3H), 7.22 (m, 1H), 7.03 (d, J = 8.1 Hz, 1H), 6.97 (t, J = 7.5 Hz, 1H), 6.87 (m, 1H), 6.05 (br, 1H, OH), 4.06 (m, 4H, CH_2), 1.93 (m, 2H, CH), 1.47-1.16 (m, 48H, CH_2), 0.89-0.80 (m, 12H, CH_3) ppm. ^{13}C NMR (101 MHz, $CDCl_3$): δ= 164.0, 163.8, 163.7, 162.9, 155.5, 153.9, 153.0, 133.9, 133.8, 133.3, 132.9, 132.0, 131.3, 130.6, 130.1, 130.0, 129.8, 129.6, 128.8, 128.6, 127.9, 126.3, 125.7, 125.3, 125.1, 124.7, 123.8, 123.4, 123.3, 122.7 (two signals), 122.2 (two signals), 120.8, 119.0, 116.0, 44.9, 44.8, 36.8 (two signals), 32.0 (two signals), 31.9, 31.8, 30.2, 30.0, 29.9 (three signals), 29.8, 29.5, 26.7, 26.6, 22.8, 14.3 ppm. HRMS (ESI, neg. mode, acetonitrile/chloroform): *m/z* = 1022.6178 $[M]^-$, calcd. for $C_{68}H_{82}N_2O_6$: 1022.6178.

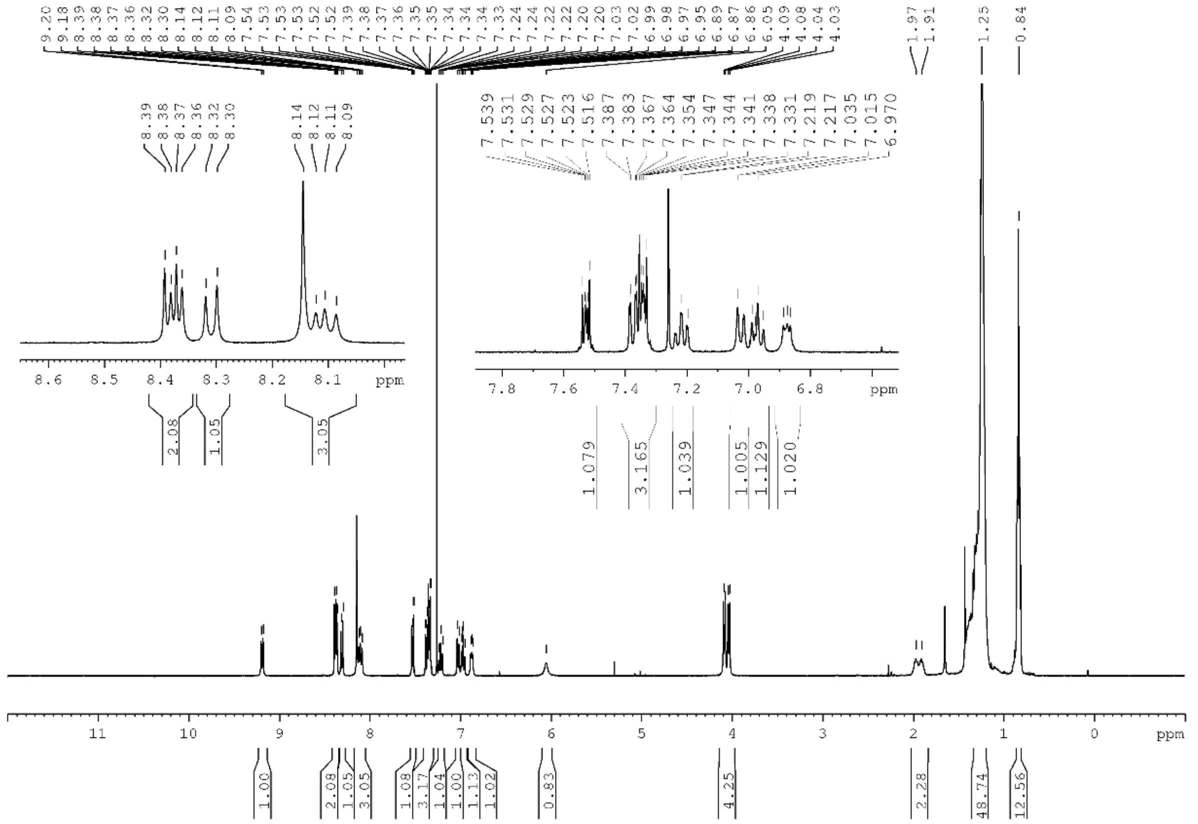

Supplementary Figure 2. ^{1}H-NMR spectrum (400 MHz) of PBI **2** in $CDCl_3$ at room temperature.

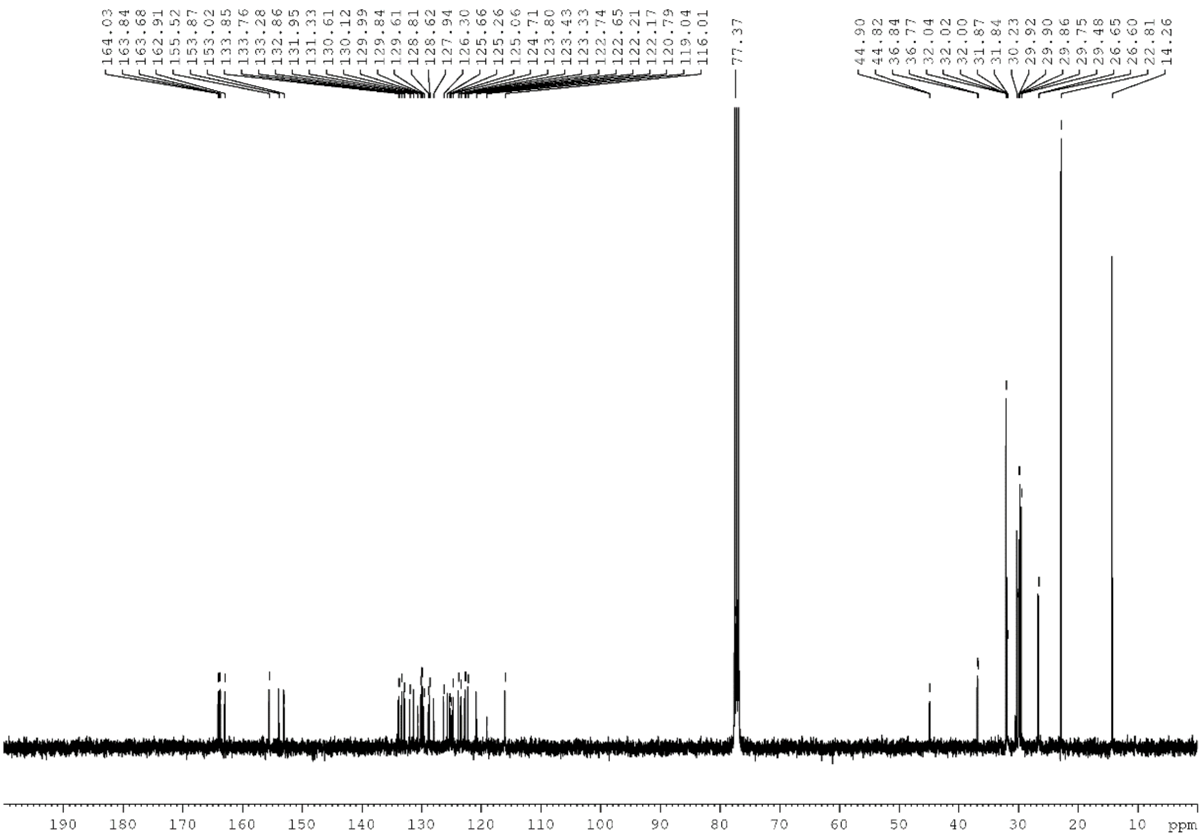

Supplementary Figure 3. 3C-NMR spectrum (101 MHz) of PBI **2** in $CDCl_3$ at room temperature.

Synthesis of 2,2'-biphenol-connected PBI dimer 4: Bay-dibrominated-PBI **3**[S33] (50 mg, 50.2 µmol), PBI **2** (102.7 mg, 100.3 µmol) and Cs_2CO_3 (163.4 mg, 500 µmol) were dissolved in THF (8 mL). The reaction mixture was stirred at 60 °C for 3d and then cooled down to room temperature. Water (10 mL) was added and the resulting mixture extracted with dichloromethane (5 × 10 mL). The combined organic phases were washed with water (3 × 10 mL) and dried over $MgSO_4$. The crude product was purified by column chromatography on silica gel (gradient dichloromethane: *n*-hexane 2:8 to 6:4 dichloromethane: *n*-hexane) to give compound **4** as a purple solid (37.0 mg, 38 %). Mp.: 185-187 °C. ^{1}H NMR (600 MHz, TCE-d_2, 380 K): δ= 9.63 (d, J = 8.1 Hz, 1H), 9.16 (d, J = 8.3 Hz, 1H), 9.03 (d, J = 8.3 Hz, 1H), 8.95 (s, 1H), 8.75 (d, J = 7.9 Hz, 1H), 8.63 (d, J = 8.2 Hz, 1H), 8.61 (d, J = 8.2 Hz, 1H), 8.54 (d, J = 8.0 Hz, 1H), 8.51 (d, J = 8.2 Hz, 1H), 8.18 (d, J = 8.2 Hz, 1H), 8.15 (d, J = 8.2 Hz, 1H), 8.09 (s, 1H), 8.06 (s, 1H), 7.83 (m, 2H), 7.50 (m, 4H), 7.11 (m, 2H), 4.15 (d, J = 7.2 Hz, 2H), 4.09 (d, J = 7.2 Hz, 2H), 3.69 (m, 4H), 2.05 (m, 2H), 1.80 (m, 2H), 1.41–1.23 (m, 96H, CH_2), 0.94–0.88 (m, 24H, CH_3) ppm. ^{13}C NMR (151 MHz, TCE-d_2, 380 K): δ= 163.4, 163.1, 162.7 (two signals), 162.6, 162.3, 162.0, 161.9, 154.9, 154.8, 152.3, 152.2, 137.9, 134.2, 134.1, 133.3, 133.2, 132.9 (two signals), 132.8, 132.4, 131.2, 130.6 (two signals), 130.2 (three signals), 130.1, 129.4, 129.1 (two signals), 128.7, 128.3, 128.2 (two signals), 127.8 (two signals), 127.0, 126.8, 125.6, 125.5, 125.1, 124.3, 123.6, 123.4, 123.3, 123.2 (two signals), 123.1, 122.9, 122.6, 122.5, 122.4 (three signals), 122.1, 121.9, 119.7, 118.9, 118.8, 44.8 (two signals), 44.7, 44.6, 36.7, 36.6, 36.4, 31.9 (three signals), 31.8, 31.6, 31.5 (three signals),

31.4 (two signals), 29.8, 29.7 (two signals), 29.4 (two signals), 29.3 (two signals), 29.2 (two signals), 29.1, 28.9 (three signals), 26.4 (three signals), 26.3 (two signals), 26.2 (three signals), 22.3, 22.2 (two signals), 13.6 (four signals) ppm. HRMS (MALDI, neg. mode, acetonitrile/chloroform): *m/z* = 1939.087 $[M]^{-}$, calcd. for $C_{124}H_{153}BrN_4O_{10}$: 1939.0776.

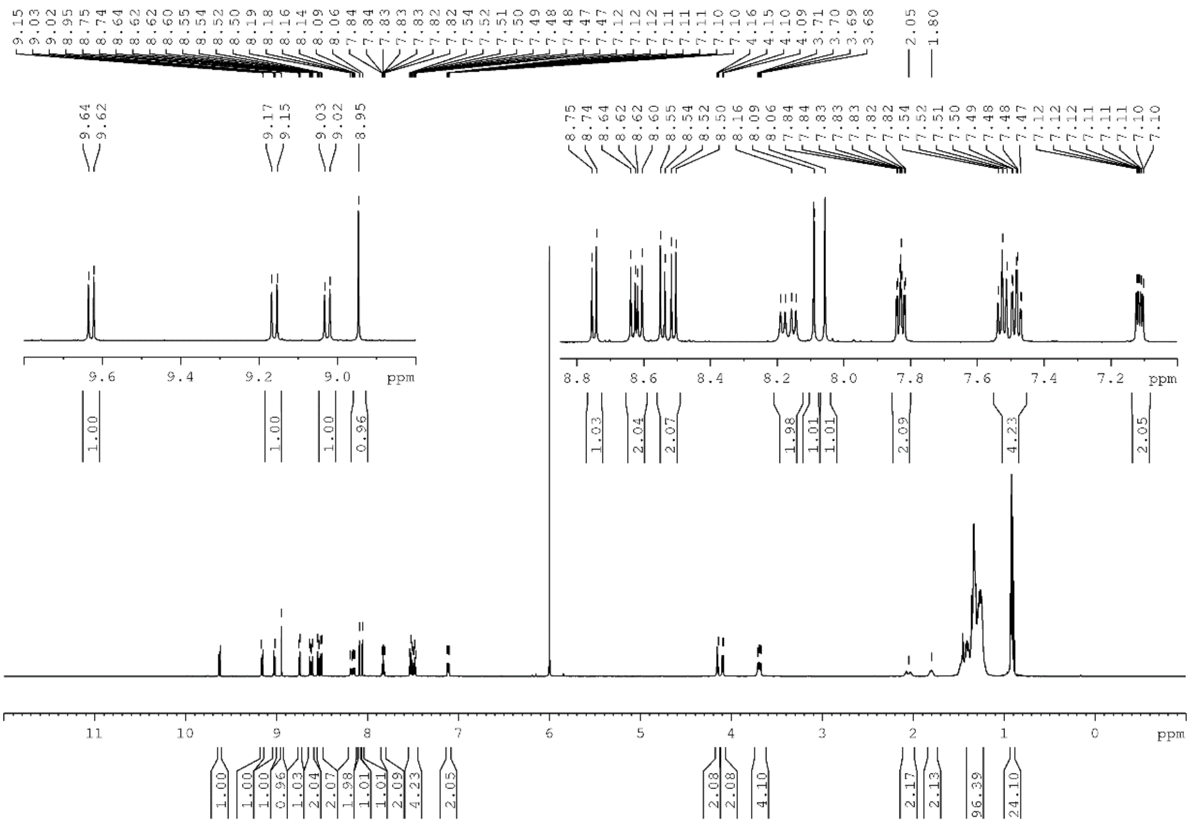

Supplementary Figure 4. ^{1}H-NMR spectrum (600 MHz) of Bis-PBI **4** in TCE-d_2 at 380 K.

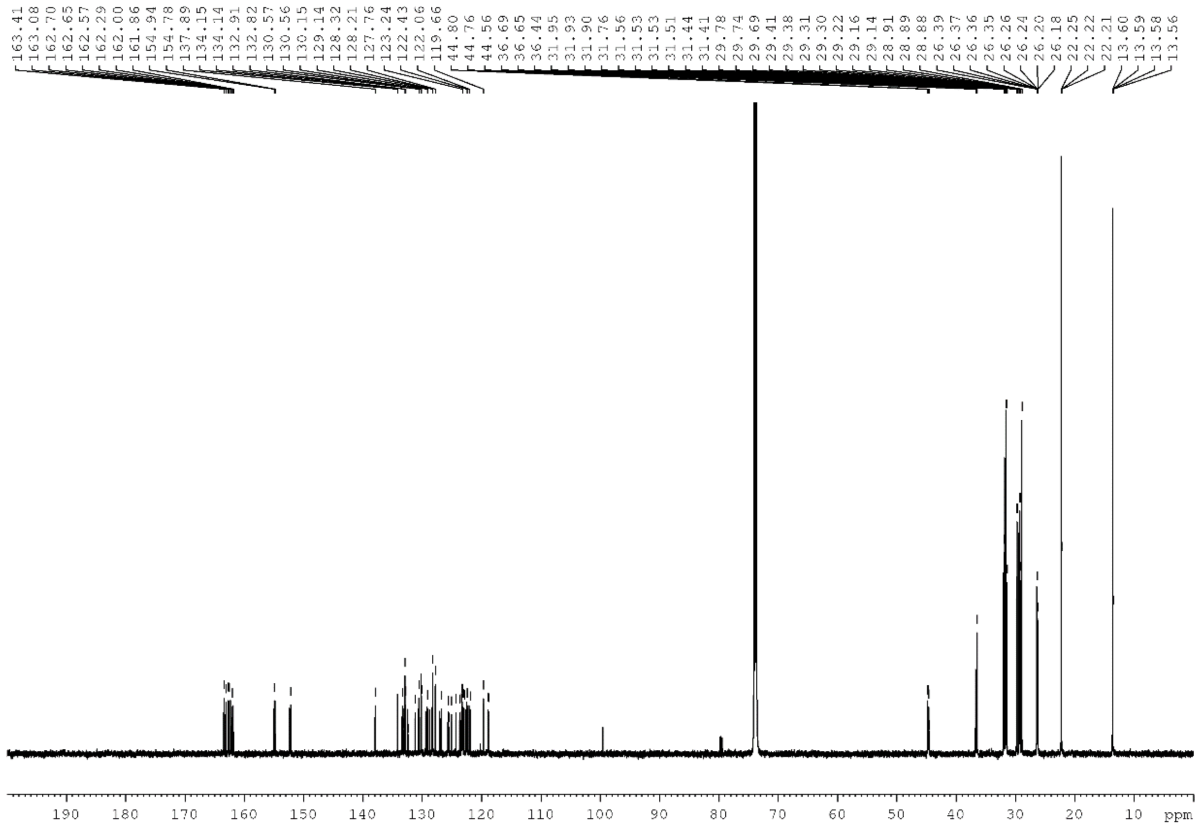

Supplementary Figure 5. ^{13}C-NMR spectrum (151 MHz) of Bis-PBI **4** in TCE-d_2 at 380 K.

Synthesis of Tris-PBI: PBI **2** (50.0 mg, 48.9 µmol), Cs_2CO_3 (47.1 mg, 244 µmol) and PBI **4** (47.4 mg, 24.4 µmol) were dissolved in THF (2 mL). The reaction mixture was stirred at 60 °C for 15 h. The mixture was allowed to cool down to room temperature, water (10 mL) was added and the resulting mixture extracted with dichloromethane (5 × 10 mL). The combined organic phases were washed with water (3 × 10 mL) and dried over $MgSO_4$. The crude product was purified by column chromatography on silica gel (DCM) to give a mixture of PBI **4** and **Tris-PBI**. The mixture was separated via GPC. Afterwards **Tris-PBI** was carefully washed with HPLC grade *n*-hexane solvent to yield **Tris-PBI** as a red solid (14.8 mg, 5.1 µmol, 21 %). Mp.: 234-236 °C. ^{1}H NMR (600 MHz, TCE-d_2, 380 K): δ= 9.14 (d, J = 8.3 Hz, 4H), 8.71 (d, J = 7.8 Hz, 2H), 8.57 (d, J = 8.3 Hz, 2H), 8.54 (d, J = 8.2 Hz, 2H), 8.45 (d, J = 7.9 Hz, 2H), 8.17 (d, J = 7.3 Hz, 2H), 8.09 (d, J = 8.3 Hz, 2H), 8.03 (s, 2H), 7.96 (s, 2H), 7.84 (m, 4H), 7.54 (m, 6H), 7.49 (m, 2H), 7.37 (m, 2H), 7.14 (d, J = 7.5 Hz, 2H), 4.10 (d, J = 7.3 Hz, 4H), 3.62 (d, J = 6.7 Hz, 4H), 3.48 (d, J = 6.7 Hz, 4H), 2.05 (m, 2H), 1.77 (m, 2H), 1.68 (m, 2H), 1.39–1.18 (m, 144H, CH_2), 0.94–0.84 (m, 36H, CH_3) ppm. ^{13}C NMR (151 MHz, TCE-d_2, 380 K): δ= 163.4, 163.0, 162.7, 162.4, 161.8 (two signals), 155.3, 154.6, 152.2, 151.9, 134.1, 134.0, 133.3, 133.0, 132.9 (two signals), 131.3, 130.7, 130.5, 130.2, 130.1 (two signals), 129.2 (two signals), 128.8, 128.7, 128.3, 127.8, 127.5, 126.7, 125.8, 125.5, 124.9, 124.2, 123.4, 123.1, 123.0, 122.8, 122.7, 122.6 (two signals), 122.5, 122.1, 122.0, 121.9, 121.5, 119.7, 119.6, 44.6, 44.4, 36.6, 36.5, 36.4, 31.9 (two signals), 31.7, 31.6 (two signals), 31.5 (four signals), 31.4, 29.8, 29.7, 29.6, 29.4 (two signals), 29.3, 29.2 (two signals), 29.1, 28.9 (two signals), 28.8, 26.4, 26.3 (two signals), 26.2, 26.1 (two signals), 22.3, 22.2 (four signals), 13.6 (five signals), 13.5 ppm. HRMS (MALDI, neg. mode, acetonitrile/chloroform): m/z = 2879.767 $[M]^-$, calcd. for C192H234N6O16: 2879.7687. UV/Vis (TCE, 298K, c = 1 × 10^{-5} M): λ_{max} (ε) = 556 nm (117600 M^{-1} cm^{-1}).

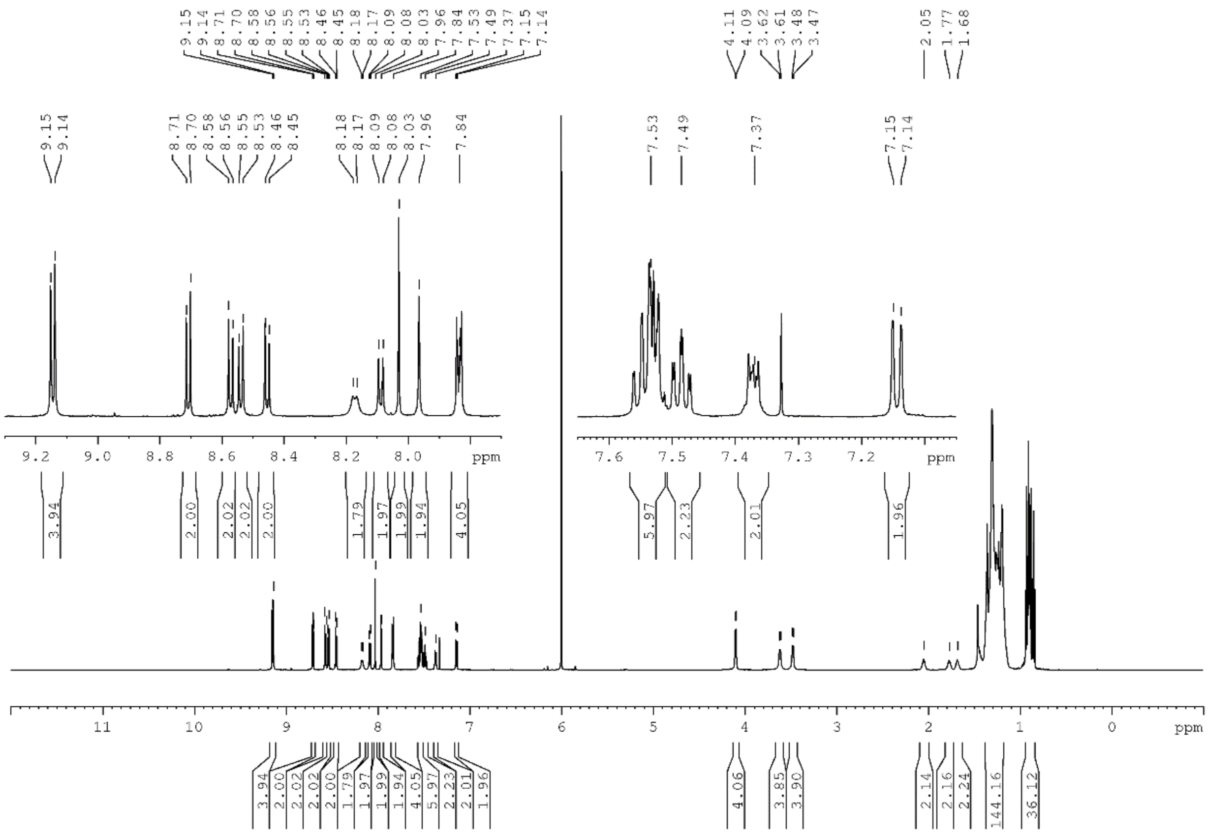

Supplementary Figure 6. ^{1}H-NMR spectrum (600 MHz) of **Tris-PBI** in TCE-d_2 at 380 K.

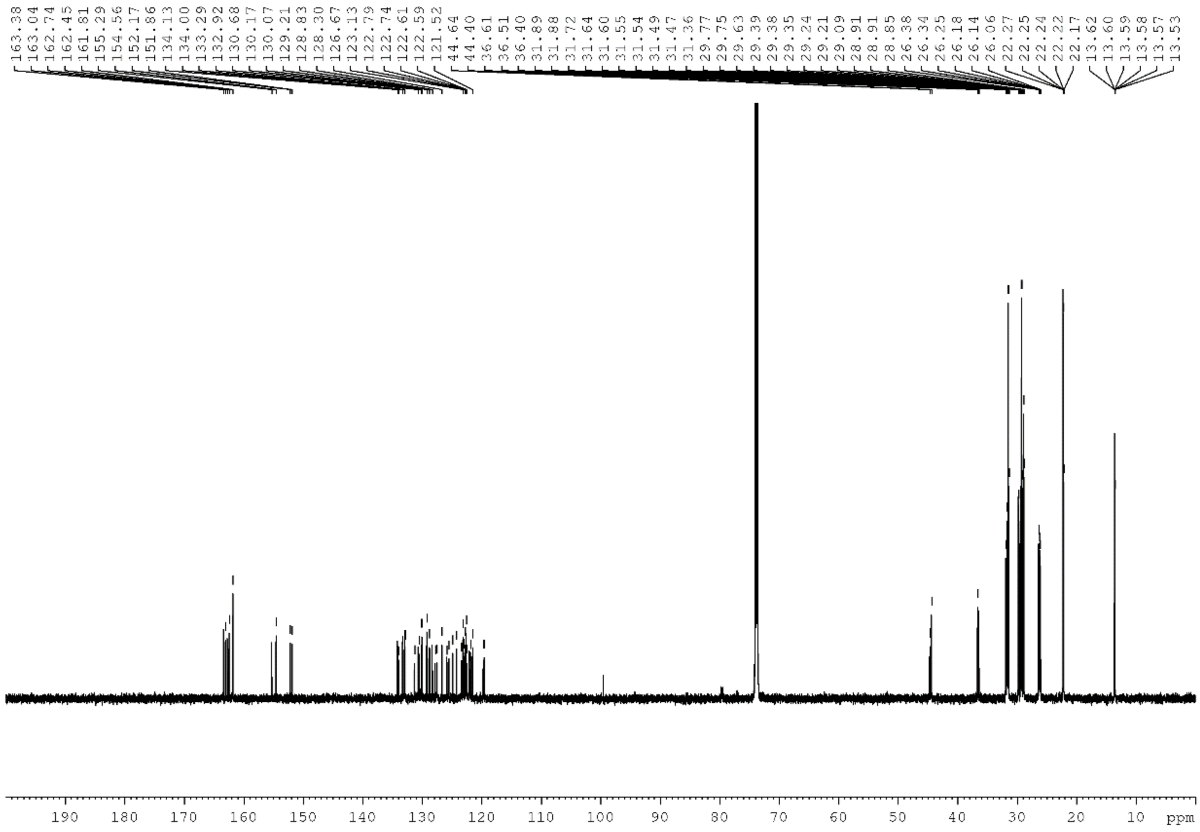

Supplementary Figure 7. ^{13}C-NMR spectrum (151 MHz) of **Tris-PBI** in TCE-d_2 at 380 K.

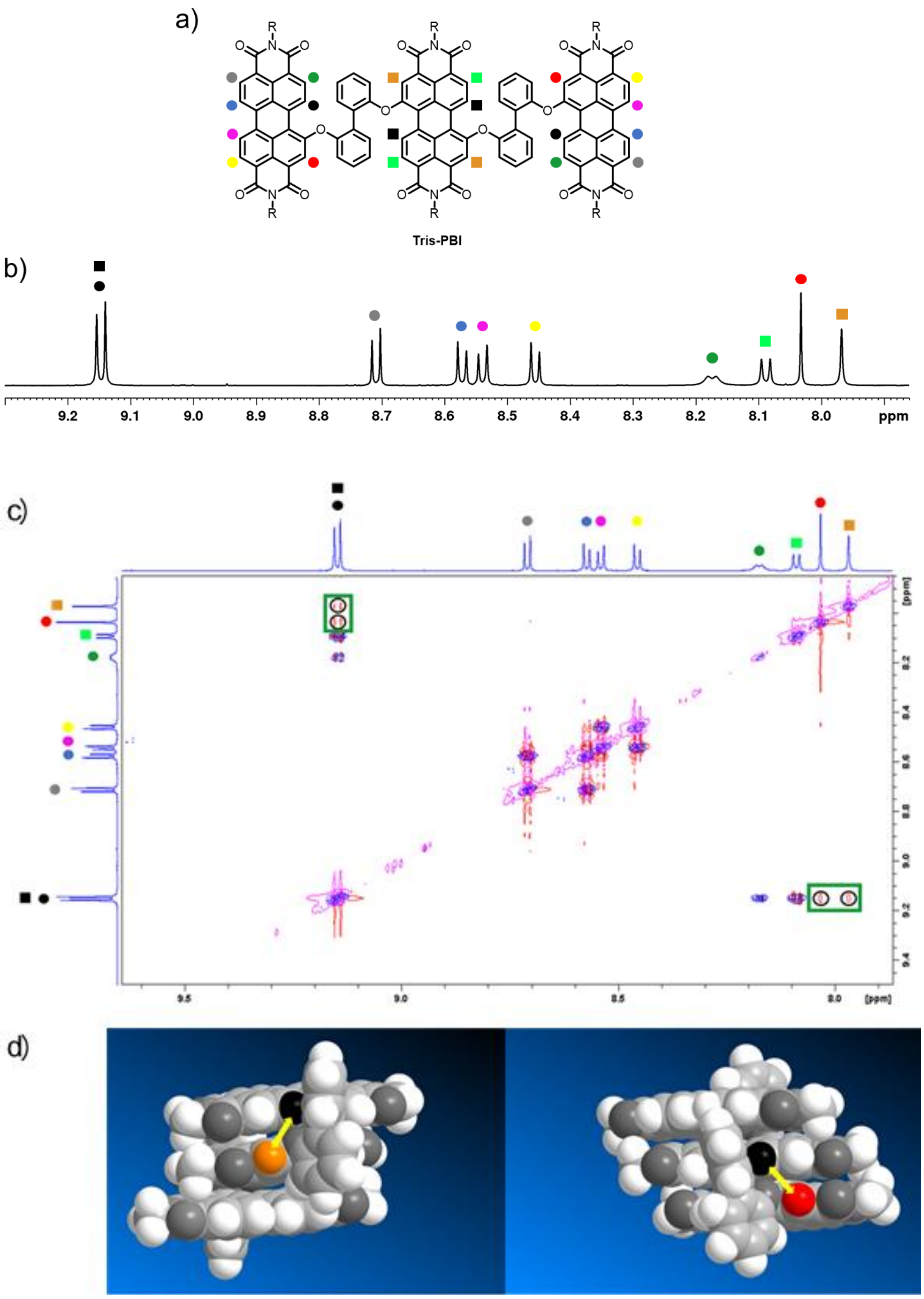

Supplementary Figure 8. a) Chemical structure of **Tris-PBI** with assigned proton signals colored. (R = 2-hexyldecyl) b) Excerpt of ^{1}H-NMR spectrum of **Tris-PBI** (600 MHz, TCE-d_2, 380 K). c) Superposition of COSY (blue) and ROESY (red: positive signal / pink: negative signal) spectra of **Tris-PBI** (600 MHz, TCE-d_2, 380 K). d) Geometry-optimized structure B97D3/def2SVP of **Tris-PBI** with marked protons interacting through space as indicated by the ROESY signals marked by green rectangles. 2-Hexyldecyl groups are replaced by methyl groups.

Supplementary Table 1. Summary of ^{1}H NMR proton chemical shifts *δ* (ppm) for PBI core protons of **Ref-PBI**, **Bis-PBI2** and **Tris-PBI** (600 MHz, TCE-d_2, 380 K).[a)]

	● / ■	●	●	●	●	●	■	●	■
**Ref-PBI**	9.60	8.77	8.70	8.72	8.75	8.69	-	8.35	-
**Bis-PBI2**	9.17	8.73	8.59	8.55	8.45	8.27	-	8.01	-
**Tris-PBI**	9.14	8.71	8.57	8.54	8.45	8.17	8.09	8.03	7.96

The similar chemical shifts observed for the protons of the outer PBI dyes of **Tris-PBI** and the related protons of **Bis-PBI2** suggest similar stacking arrangements. The fact that all signals for **Bis-PBI2** and **Tris-PBI** are upfield shifted compared to **Ref-PBI** is attributable to the diamagnetic ring current of the neighboring PBI unit.

a)

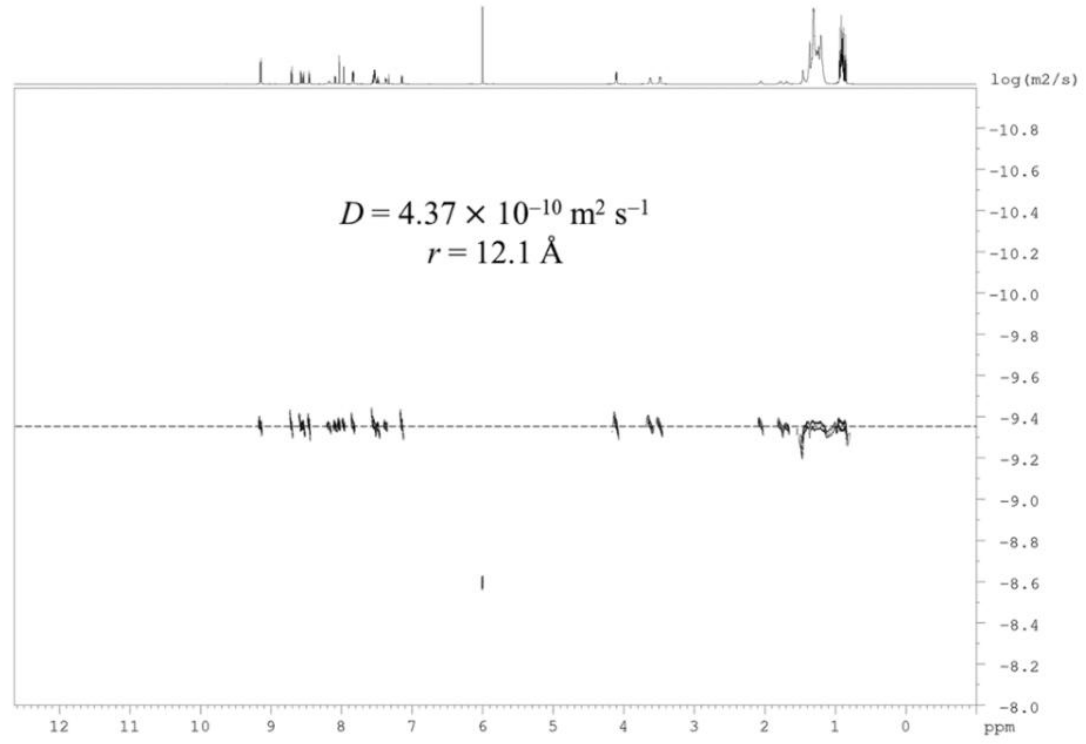

b)

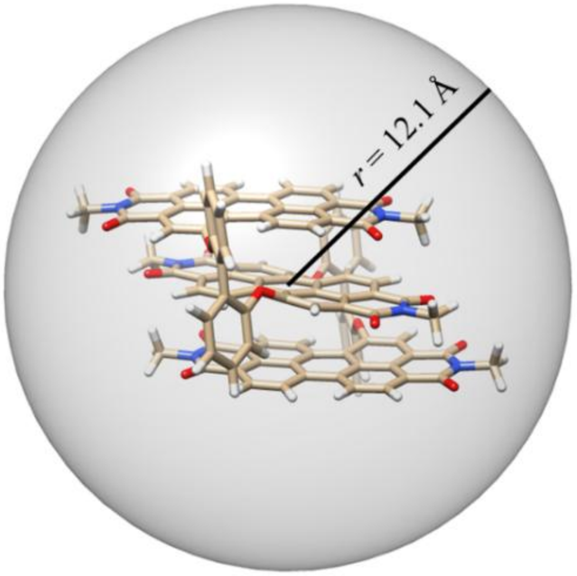

Supplementary Figure 9. a) 2D DOSY NMR spectrum (600 MHz, TCE-d_2) of **Tris-PBI** measured at 380 K. The Stokes-Einstein equation has been applied to obtain the hydrodynamic radius. b) Geometry-optimized structure (B97D3/def2SVP) of the folded state of **Tris-PBI** (2-hexyldecyl groups are replaced by methyl groups). In addition, the grey sphere indicates the hydrodynamic radius of r= 12.1 Å as determined from the DOSY NMR experiments.

Supplementary Table 2. Crystal data and structure refinement.

Identification code	CCDC 2124115	
Empirical formula	$C_{200.29}$ H_{234} N_6 $O_{24.29}$	
Formula weight	3114.10	
Temperature	100(2) K	
Wavelength	0.61992 Å	
Crystal system	Tetragonal	
Space group	$I4_1$	
Unit cell dimensions	*a* = 27.947(11) °	*α* = 90 °
	b = 27.947(11) °	*β* = 90 °
	c = 46.331(17) °	*γ* = 90 °
Volume	36186(31) $Å^3$	
Z	8	
Density (calculated)	1.143 g/cm^3	
Absorption coefficient	0.056 mm^{-1}	
F(000)	13376.6	
Crystal size	0.200 × 0.200 × 0.050 mm^3	
Theta range for data collection	0.742 to 28.005°	
Index ranges	$-33 \leq h \leq 31$, $-35 \leq k \leq 35$, $-59 \leq l \leq 59$	
Reflections collected	322641	
Independent reflections	50919 [*R*(int) = 0.0363]	
Completeness to theta = 21.836 °	99.3%	
Absorption correction	None	
Refinement method	Full-matrix least-squares on F^2	
Data / restraints / parameters	50919 / 2915 / 2954	
Goodness-of-fit on F^2	0.967	
Final *R* indices [$I > 2\sigma(I)$]	R_1 = 0.0870, wR_2 = 0.2123	
R indices (all data)	R_1 = 0.1426, wR_2 = 0.2844	
Absolute structure parameter	0.5	
Extinction coefficient	n/a	
Largest diff. peak and hole	0.250 and −0.286 e·$Å^{-3}$	

Single crystal X-ray structure analysis

a)

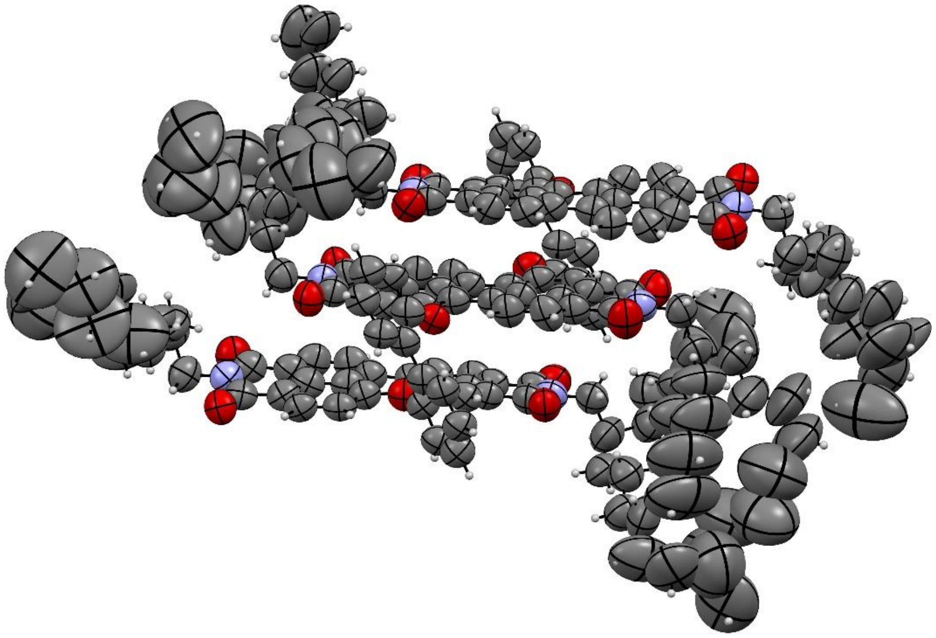

b)

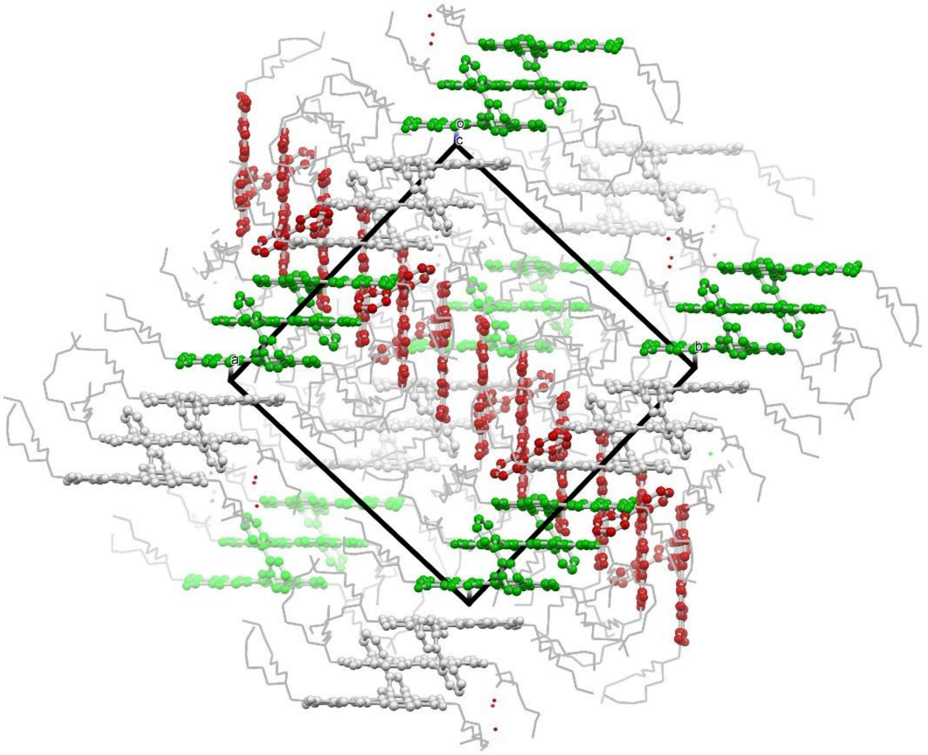

Supplementary Figure 10. Crystal structure of **Tris-PBI**. (a) A single molecule of **Tris-PBI** (ORTEP drawing in 50% probability for thermal ellipsoids) (b) Packing structure of **Tris-PBI**. Due to heavy disorder, only one representative set of imide side-chains are shown. Disordered methanol molecules are removed for clarity.

Supplementary Table 3. Comparison of selected parameters of the crystallographic analysis of **Tris-PBI** with three possible space groups. All dodecylhexyl chains were replaced with methyl group and electron density from the remaining space was removed by the SQUEEZE routine. The same measurement dataset was used for all refined structures

Space group	$I\bar{4}$	$I4_1$	$I4_1/a$
Independent reflections	50928	50919	25792
R(int)	0.0364	0.0363	0.0375
Goodness-of-fit on F^2	1.305	1.271	2.015
R index [$I > 2\sigma(I)$]	0.1446	0.1376	0.2118
$wR2$ index [$I > 2\sigma(I)$]	0.3309	0.3205	0.5194
R index (all data)	0.1950	0.1876	0.2443
$wR2$ index (all data)	0.4122	0.4031	0.5615
Largest diff. peak and hole	0.823 and -0.376 e·Å^{-3}	0.855 and -0.328 e·Å^{-3}	1.746 and -0.751 e·Å^{-3}

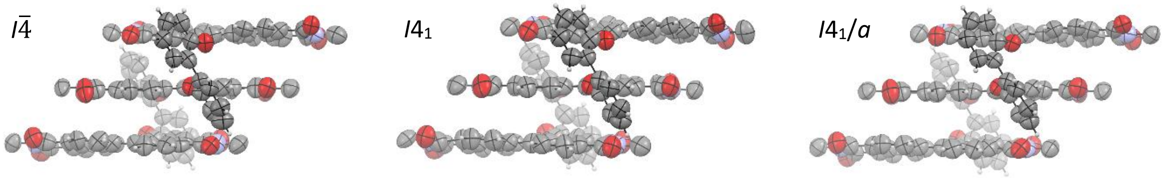

Steady-state absorption and fluorescence spectroscopy

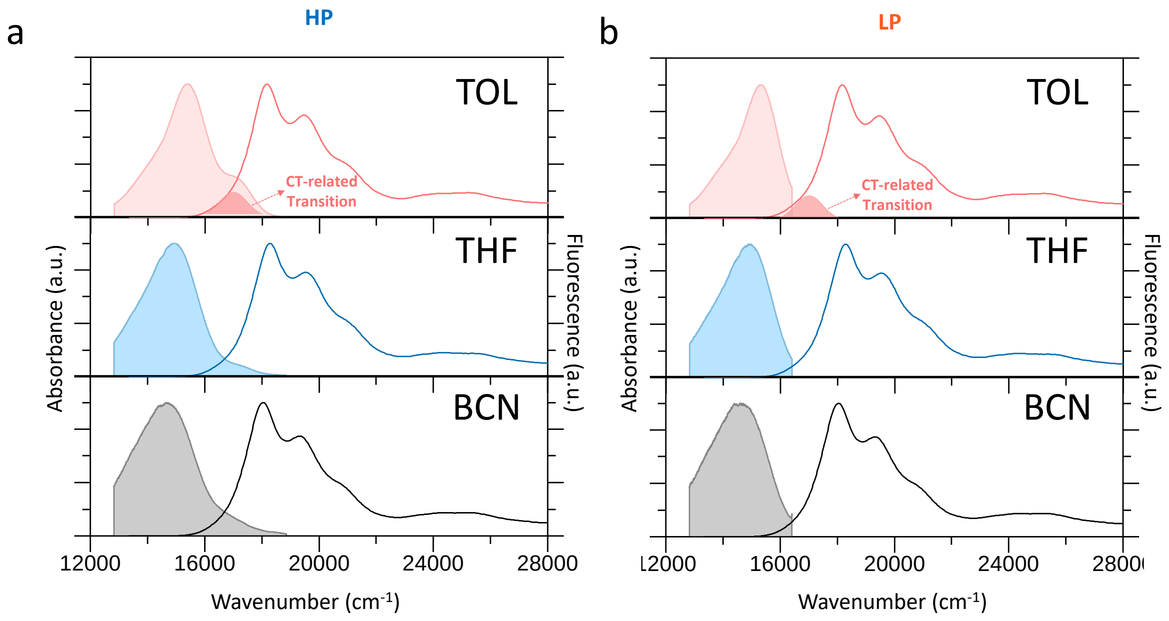

Supplementary Figure 11. The solvent-dependent absorption and fluorescence spectra by HP (**a**) and LP (**b**) in TOL (top), THF (middle), and BCN (bottom).

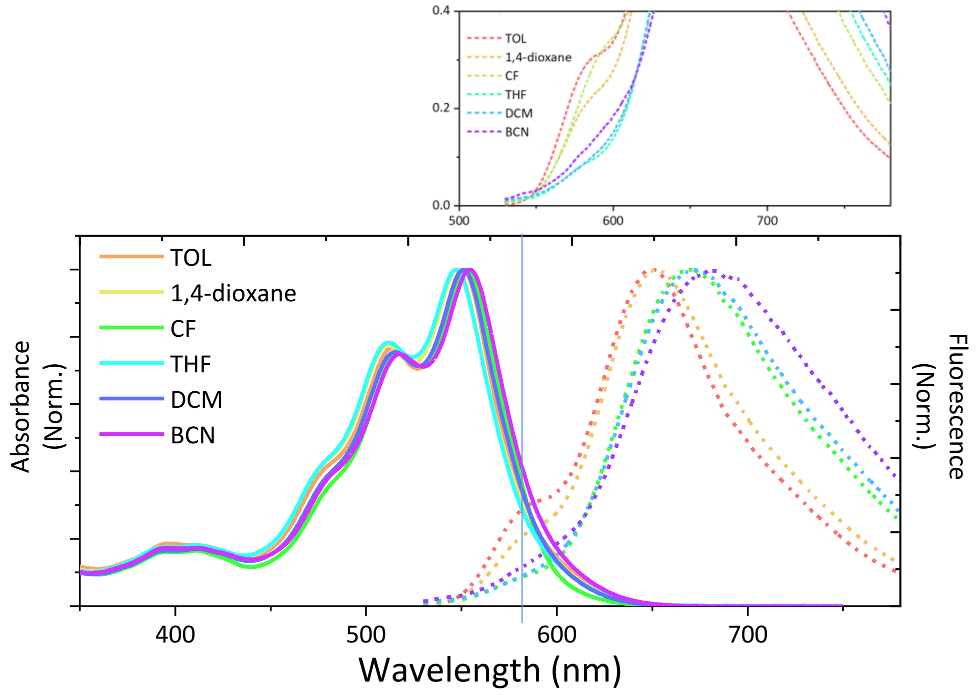

Supplementary Figure 12. The solvent-dependent absorption and fluorescence spectra by HP (TOL, 1,4-dioxane, chloroform, tetrahydrofuran, dichloromethane, and bezonitrile). The upper figure indicates the negligible peak shift of LE emission upon the solvent polarity.

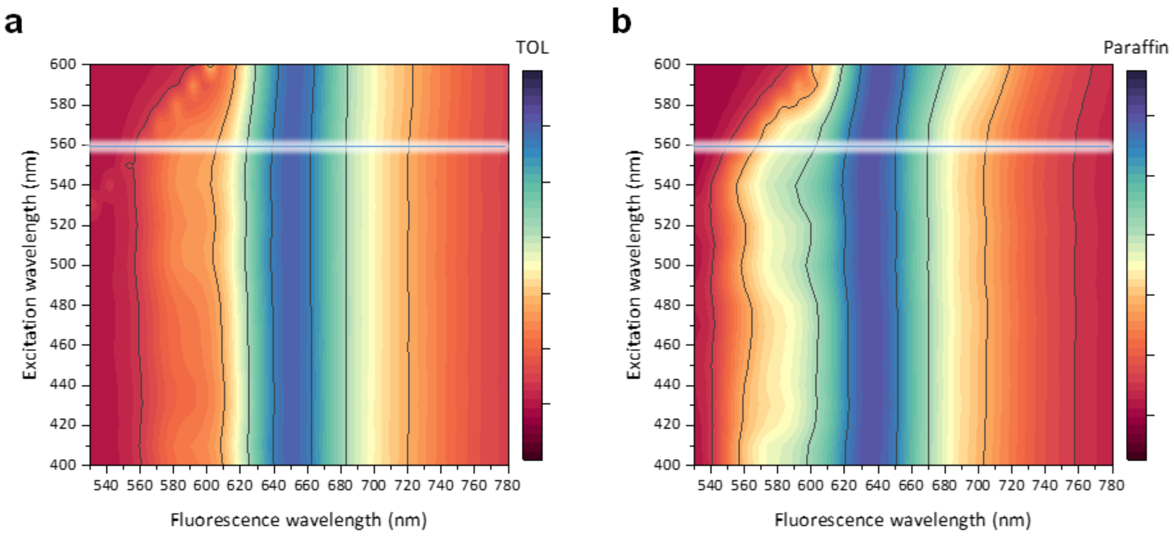

Supplementary Figure 13. The normalized excitation-fluorescence 2D contour map in TOL (**a**) and Paraffin (**b**). The blue line indicates the boundary.

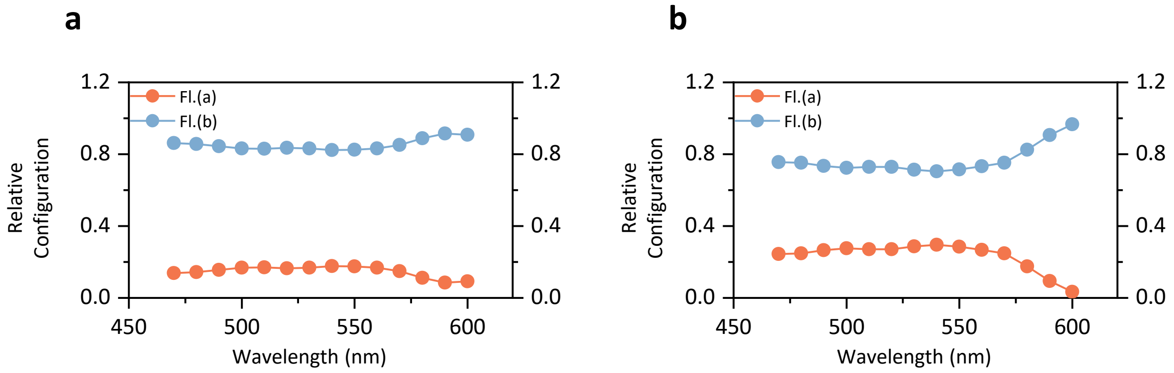

Supplementary Figure 14. The relative ratio of excitation-energy-dependent fluorescence in TOL (**a**) and Paraffin (**b**). Fl.(a) and Fl.(b) indicate the LE and ME fluorescence, respectively.

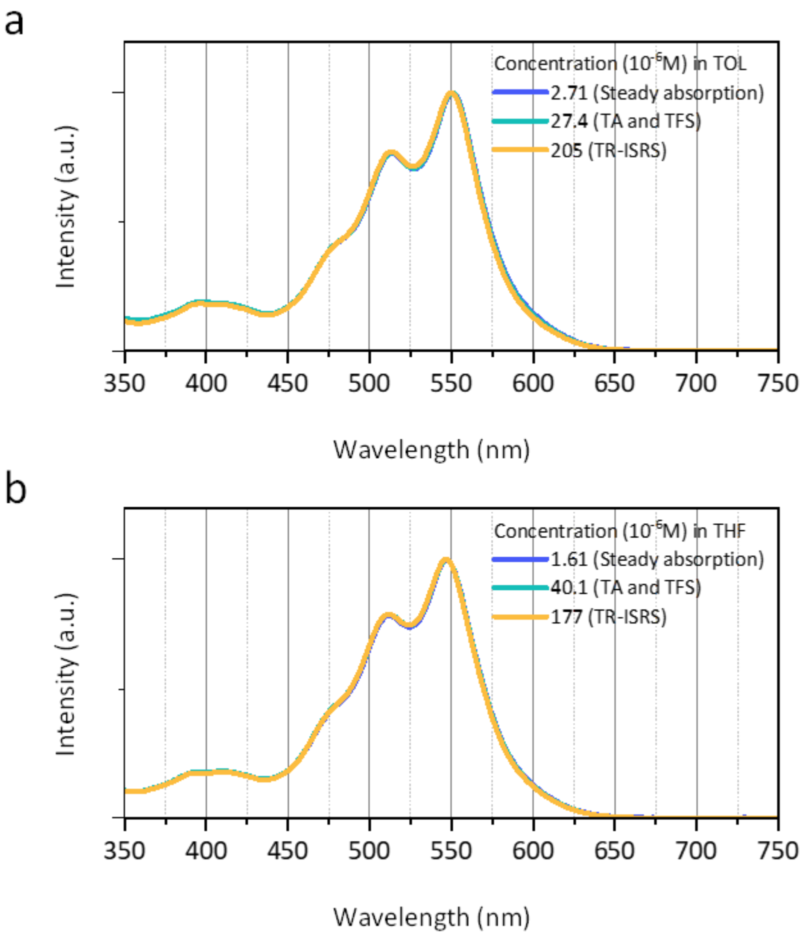

Supplementary Figure 15. The concentration-dependent absorption spectra of **Tris-PBI**. **a**, TOL. and **b**, THF. Since the negligible absorption change is observed in our experimental condition (~ 2x10^{-4}M), we rule out the further aggregation effect

Time-resolved electronic spectroscopy

a) Transient absorption

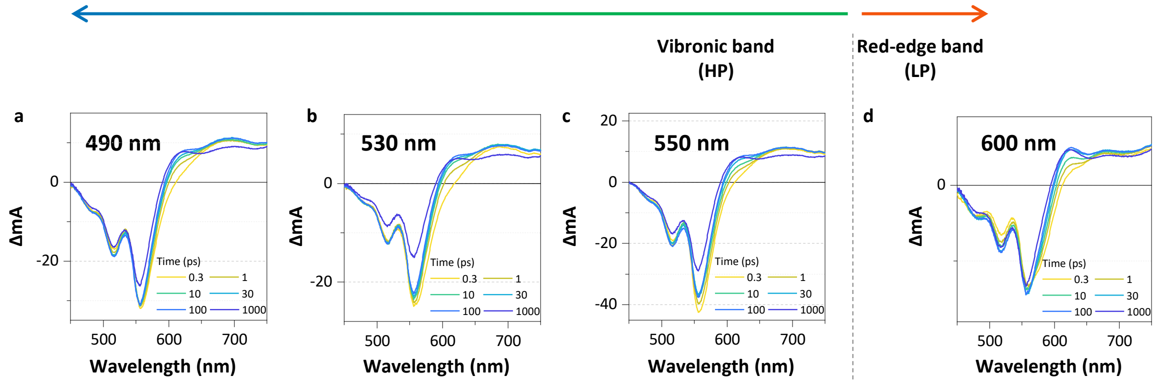

Supplementary Figure 16. The TA spectra upon photoexcitation at 490 (a),530 (b), 550 (c), and 600 (d) nm.

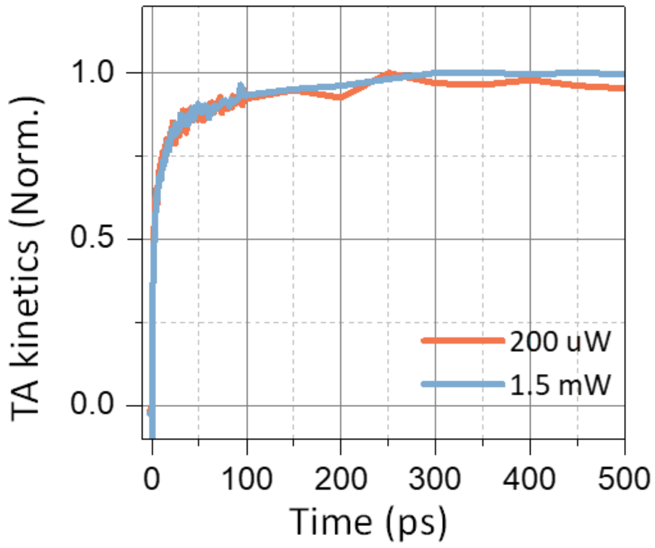

Supplementary Figure 17. The pump-fluence dependent TA kinetics of T**irs-PBI** in TOL upon HP at 620 nm.

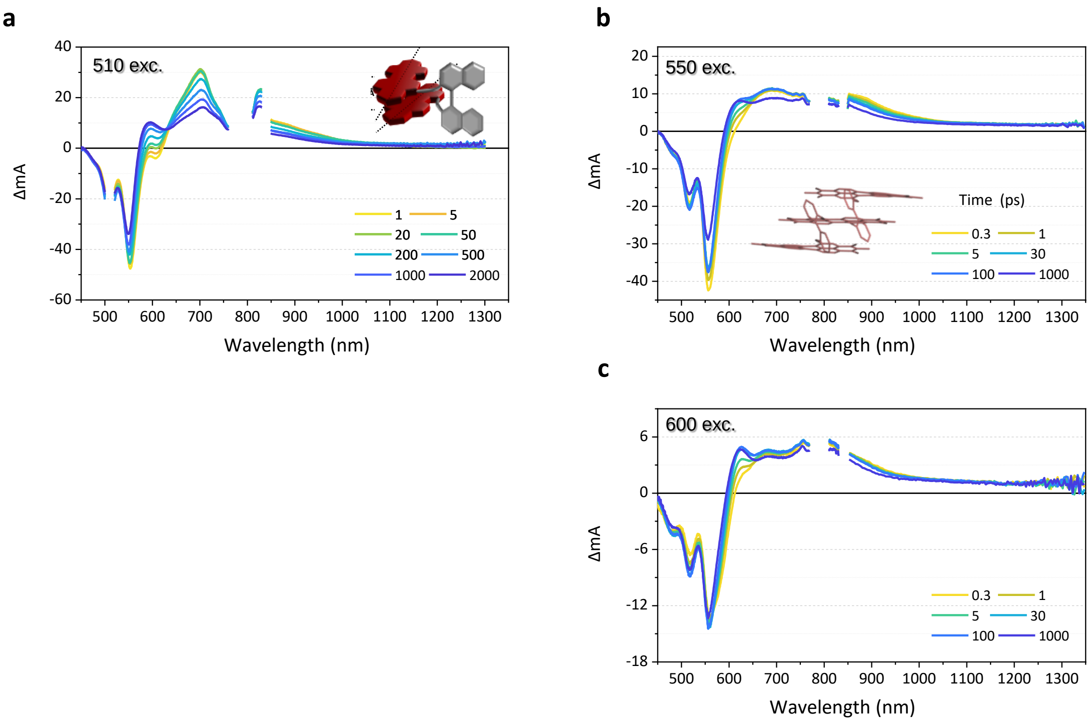

Supplementary Figure 18. The representative spectra of **Bis-PBI 2**(**a**) and **Tris-PBI** (**b, c**) in TOL. The broad ESA band of **Tris-PBI** indicates the complex excited-state potential surface compared to **Bis-PBI2**.

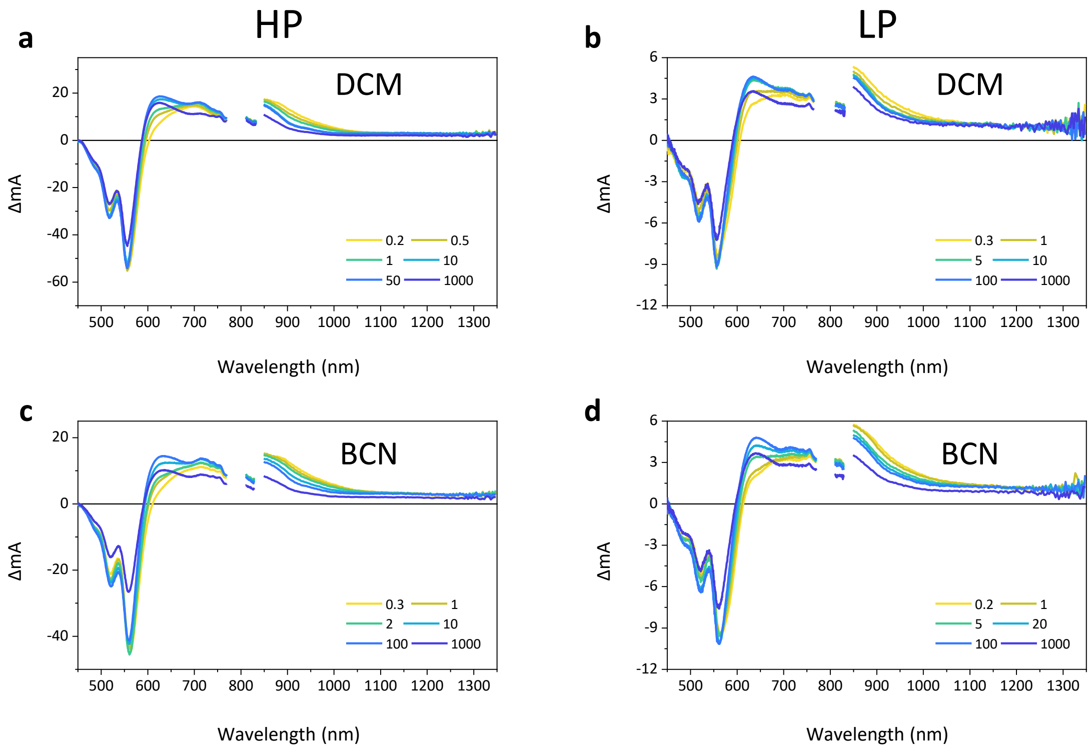

Supplementary Figure 19. The solvent-polarity controlled TA measurements of **Tirs-PBI**. **a**,**b**, The representative spectra in DCM by HP (**a**) and LP (**b**). **,-d**, The representative spectra in BCN by HP (**c**) and LP (**d**).

Supplementary Note 1. The importance of structural dynamics.

The TA kinetics of **Tris-PBI** become slow down in paraffin compared to those in TOL irrespective of the excitation wavelength. These results show that all sub-ns processes are associated with structural dynamics. Furthermore, the inefficient multiexciton generation process (k_{MEG}<(1ns)$^{-1}$) is observed in paraffin oil, indicating the excimer-mediated mechanism should be accompanied for structural fluctuations.

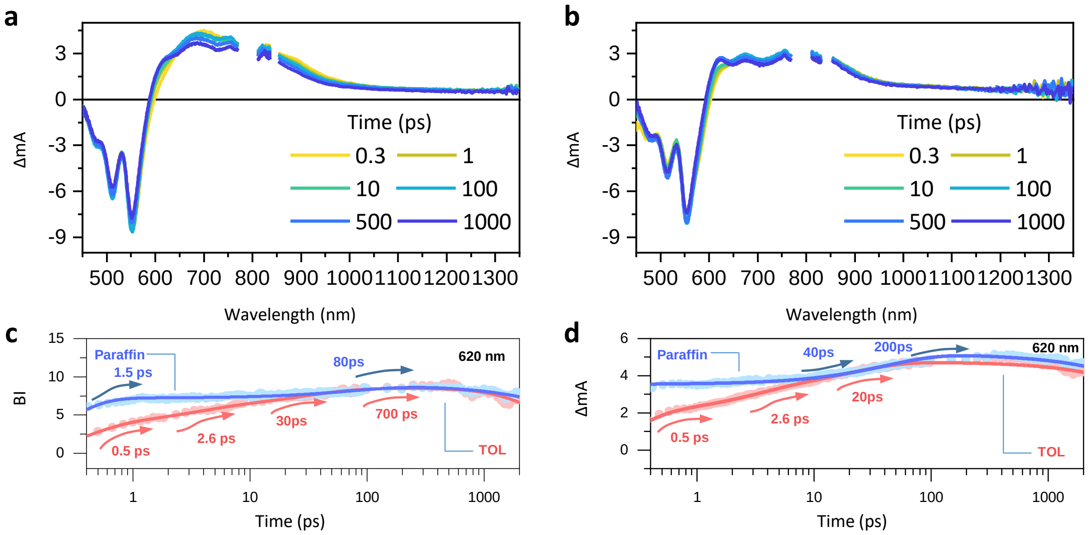

Supplementary Figure 20. The viscosity-controlled TA measurements of **Tirs-PBI** in Paraffin. **a**,**c**, the representative spectra (**a**) and TA kinetics (**c**) by the HP. **b**,**d**, The representative spectra (**b**) and TA kinetics (**d**) by the LP. The TA kinetics in TOL is overlayed for comparison.

Supplementary Note 2. The assignment of excited species.

Here, we note two points: (1) temporal resolution. the temporal resolution of fs-TA (~350 fs) is close to the initial dynamics (<500 fs) so that the initial EAS is slightly distorted in TA. (2) large spectral overlap between distinct species (FE, ME, excimer, charged species). Therefore, we can quantitively analyze the spectral evolution of the distinct species by combination of transient absorption (TA), transient fluorescence (TF), and time-resolved impulsive stimulated Raman spectroscopy (TR-ISRS) as well as referring to the literature.

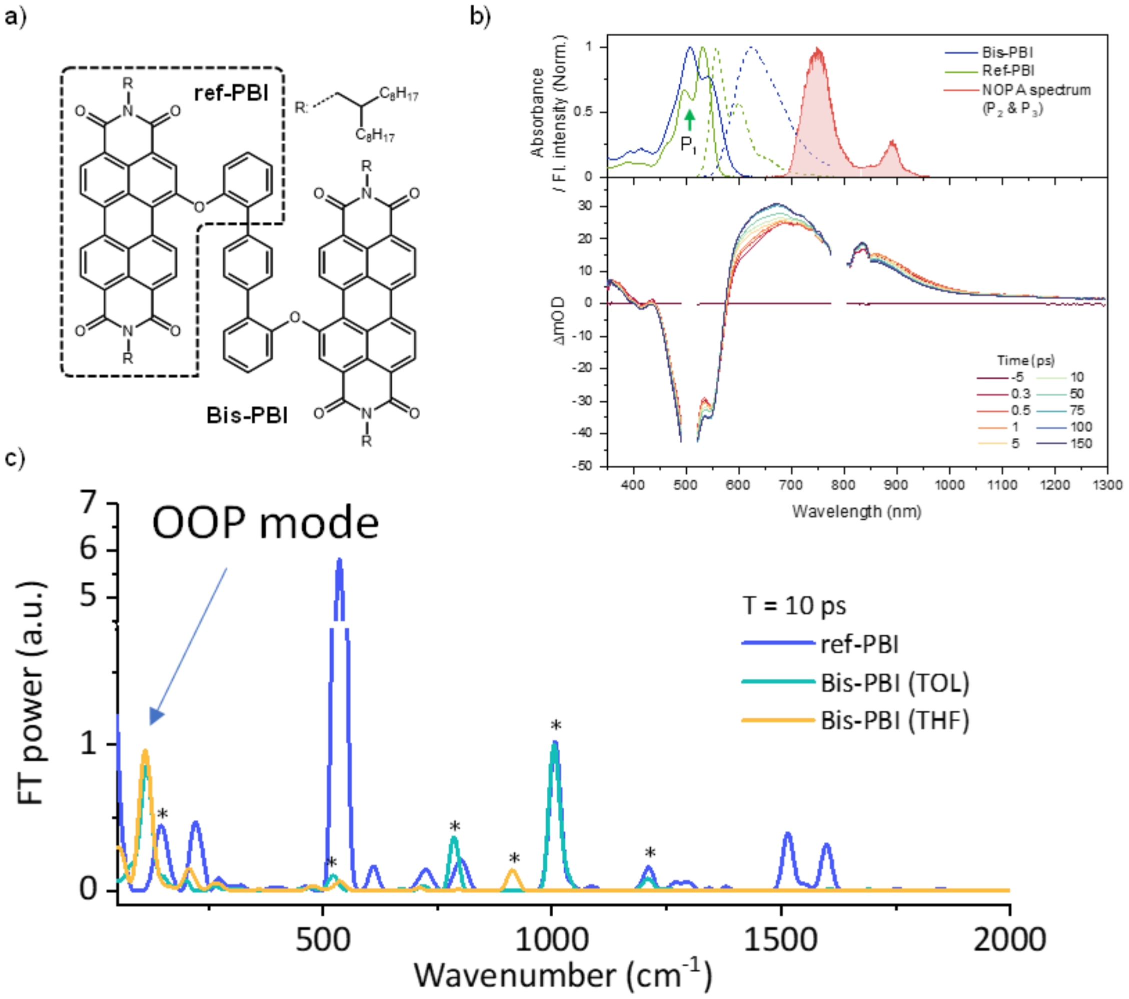

Supplementary Figure 21. (a) The chemical structure of **ref-PBI** and **Bis-PBI**. **Bis-PBI** is a representative for the excimer. (b) The steady state optical properties and TA spectra. (c) The excited state Raman spectra. Reproduced from Supplementary Reference 35 (an open access article).

By analyzing TA, TF, TR-ISRS (Figures 3,4, 5, and Supplementary Figure 21), we assigned the distinct species such as FE, FE_{CT}, $FE_{Ex.}$, and ME states.

(i) **FE state.** As shown in Figure 3a, the excited state absorption (ESA) band at ~900 nm is a most prominent feature (red line in Figure 3a) and distinct stimulated emission (SE) is observed. Moreover, distinct vibronic feature in TFS indicates the FE nature of the initial state (Figures 3a and 4c).

(ii) **FE-Ex. state.** As we described in the main text, the excimer is defined by an admixture of FE and CT excitons. In Figure 4a and 4b, TFS becomes broad and slightly red-shifted, indicating the mixing of the CT state with FE state. Considering that toluene is weakly polar solvent, the FE state is evolved into the

CT enhanced FE state via structural rearrangement rather than solvent fluctuation. Therefore, we assigned the intermediate as $FE_{EX.}$ state in that the structural rearrangement which induces the perturbation of the electronic state is the distinct feature of the excimer formation [S34-S37] Moreover, Figure S19 distinctly manifests that the structural rearrangement plays an important role in the increase of CT character in FE state. In viscous medium, initial process slows down and the spectral feature shows minor changes with time compared to that in TOL. Finally, the excited-state Raman spectra indicate the excimer-like intermediate in that the structural change gives rise to the electronic perturbation leading to an admixture of FE and CT states. In both theoretical and experimental aspects, the interchromophore out-of-plane mode is regarded as a key reaction coordinate for the excimer formation[S32-S35]. In this regard, the prominent rise of OOP mode upon the HP against the TA dynamics indicates that the intermediate state shows a characteristic of the excimer state.

(iii) **FE_{CT} state.** Unlike the excimer requiring the structural rearrangement, excitation of the CT band (LP) gives rise to the large CT character in FC geometry, which is shown in broad and featureless TA and TF spectra (Figures 3c and 4f).

(iv) **ME state.** The rise of T_1-T_n band at 630 nm affords the generation of the ME state (Supplementary Figure 22). Although the ME state in PBI systems is quite complex in that ME state is a superposition of the LE, CR, and TT diabats, which makes assignment of specific states difficult, the distinct rise of T_1-T_n band in weak polar medium enables us to differentiate between ME and the other states (TA spectra for the excimer in Supplementary Figure 21). For example, the ultrafast MEG process (~500 fs) upon the LP gives rise to the prominent rise of the TT band at 630 even though the TF intensity increases with the same time constant. In contrast, the TA spectra of excimer show a rise of broad ESA bands (600-900 nm).

b) *ns*-TA

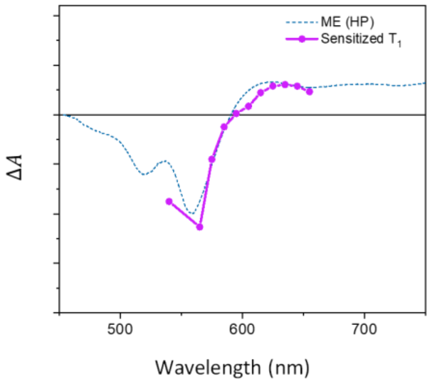

Supplementary FigureS22. T_1 spectrum of **Tris-PBI** in TOL.

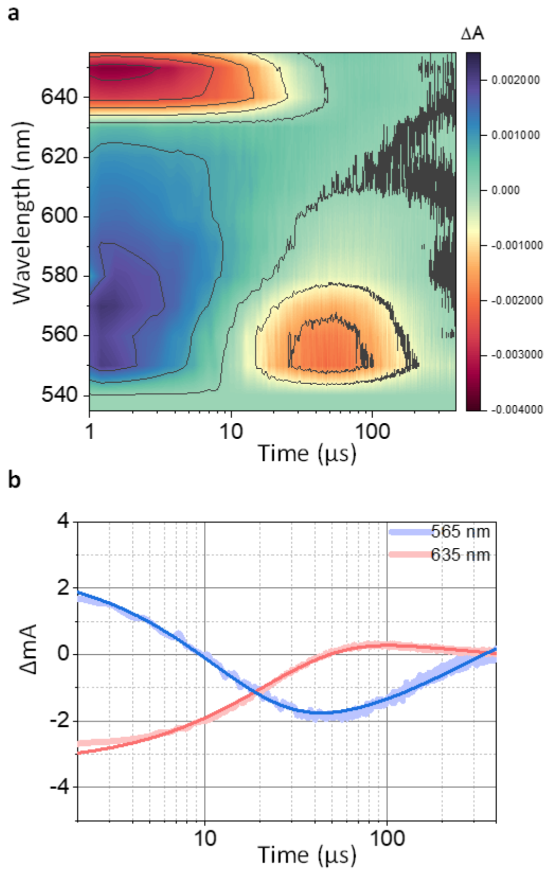

Supplementary Figure 23. The *ns*-TA measurements at photoexcitation at 355 nm in TOL. **a**, 2D contour map of **Tris-PBI** with PtOEt in TOL. **b**, The *ns*-TA kinetics at 565 and 635 nm.

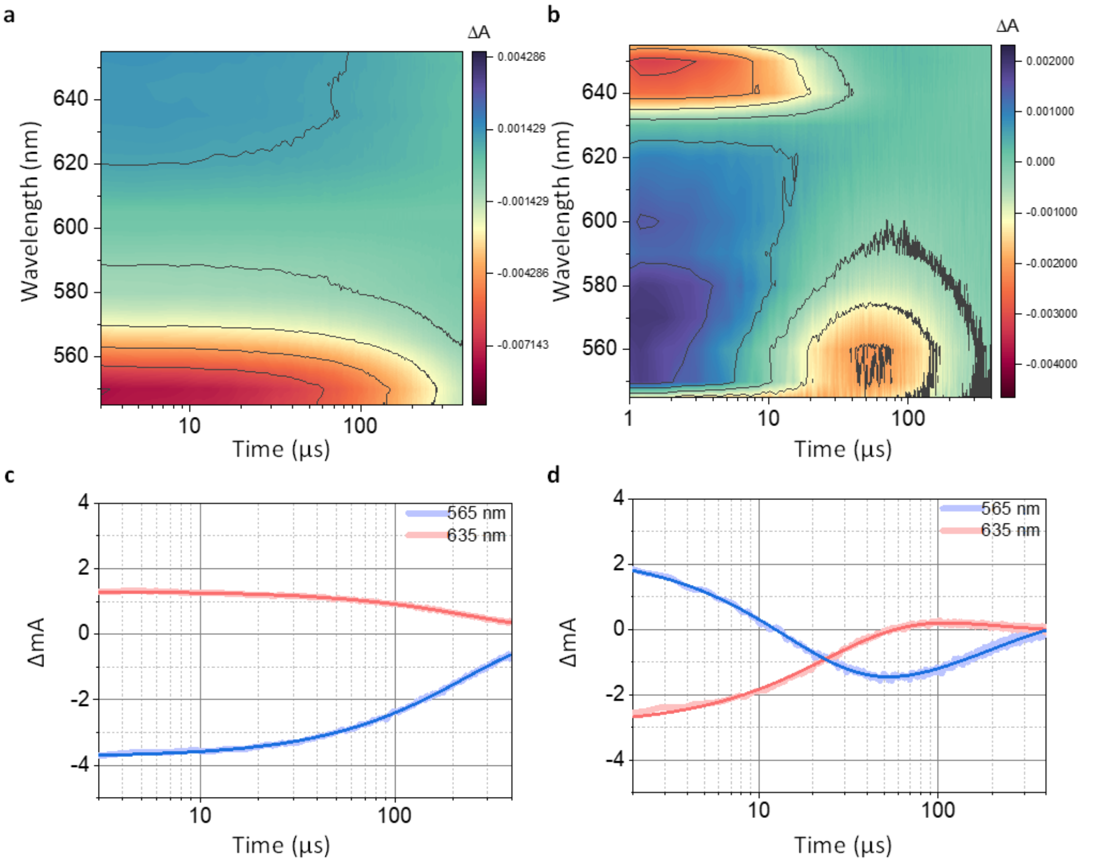

Supplementary Figure 24. The *ns*-TA measurements of **Tris-PBI** in THF. **a**,**b**, The 2D-contour map of *ns*-TA measurements at photoexcitation at 555 nm in THF (**a**) and TA kinetics (**b**). **c**,**d**, the 2D-contour map of *ns*-TA measurements with PtOEt (**c**) and the *ns*-TA kinetics at 565 and 635 nm (**d**).

Supplementary Note 3: calculation of the multiexciton generation yield.[S7]

For the triplet sensitizer, we used platinum octaethylporphyrin (PtOEP), which has an 100 % of QY_T. We obtained triplet extinction coefficient of **Tris-PBI** using triplet-triplet energy transfer. Using sensitization method, SF yields shows large error as much as 20 % depending upon the probe wavelength. The overestimated SF yield is most likely due to the spectral overlap of LE, TT, and CR bands. Furthermore, as the GSB at 560 nm contains SE signal, the singlet concentration is overestimated. Therefore, to minimize the contamination from the spectral overlap, we used the TA spectra at 1 ns (LP) and 2 ns (HP), where not only SE signal is minimized but also TT signature becomes prominent.

The concentration of triplet exciton for **Tris-PBI** is evaluated by the following procedures.

(1) Triplet energy transfer efficiency.

Photons/Pulse = $\frac{power}{(rep\ rate)(energy\ per\ photon)}$ = 1.97E+13

Spot volume; V = Area•d = 0.00201 L

The fraction of light intensity transmitted (I/I_0) of PtOEP at 335 nm

I/I_0 = $10^{-\varepsilon_{355nm}c_{PtOEP}L}$ = 0.02885 (extinction @ 355 nm: 11229.15718 $cm^{-1}M^{-1}$, c: $1.1 \cdot 10^{-4}$ M, and L:1 cm)

The concentration of triplet exciton for PtOEP:

$c_T^{PtOEP} = \frac{(\frac{photons}{pulse})(1-\frac{I}{I_0})}{N_A V}$ = 1.156E-08 mol/L (in case of **Tris-PBI** in toluene)

Triplet energy transfer efficiency (Φ_{TET}) was calculated by the ratio of triplet energy transfer rate (τ_{TET}) over the sum of the energy transfer and the triplet lifetime of PtOEP ($\tau_{T,PtOET}$).

Φ_{TET} = $\frac{\frac{1}{12.0}}{\frac{1}{12.0}+\frac{1}{40.0}}$ x 100= 76.9%

(2) Triplet concentration of **Tris-PBI** (c_T^{PBIs})

We approximate that triplet exciton of PtOEP transfers to **Tri-PBI** with 76.9 % of efficiency. Therefore, the triplet concentration of **Tris-PBI** n is calculated by following Beer's law.

c_T^{PBIs}= 1.156E-08 • 76.9 = 8.89E-08 mol / L

Using the triplet concentration and ns-TA spectra at 30 µs, we obtained triplet extinction coefficient depending upon the probe wavelength.

Ex.) $\varepsilon_T^{625\ nm}$ = $\frac{(A)_{625nm}}{c_T^{PBIs}{}_A L}$ = 31637.38158 mol/L and $\varepsilon_T^{625\ nm}$ = $\frac{(A)_{625nm}}{c_T^{PBIs}{}_A L}$ = 21355.92162 mol/L

(2) SF yield

Finally, the SF yield is calculated.

$$Q_{SF} = \frac{\varepsilon_S^{max}}{\varepsilon_T^{xx\ nm}} \frac{OD_{ME}}{OD_{S1}} \qquad (3)$$

Table S4. The SF yield.

		625 nm		635 nm		645 nm		Averaged value
HP (550 nm)	$\varepsilon_T^{xx\ nm}$	31637.38158		21355.92162		24120.34646		-
	OD_{ME}	0.00815		0.00786		0.00751		-
	OD_{S1}	-0.02893		-0.02893		-0.02893		-
	SF yield	104.7%		145.6%		126.6%		127.0% (±18%)
LP (600 nm)	$\varepsilon_T^{xx\ nm}$	31637.38158		21355.92162		24120.34646		-
	OD_{ME}	0.00463		0.0043		0.00378		-
	OD_{S1}	-0.01334		-0.01334		-0.01334		-
	SF yield	129.0%		177.5%		138.2%		148.0% (±21%)

c) Time-resolved fluorescence (transient fluorescence upconversion and TCSPC)

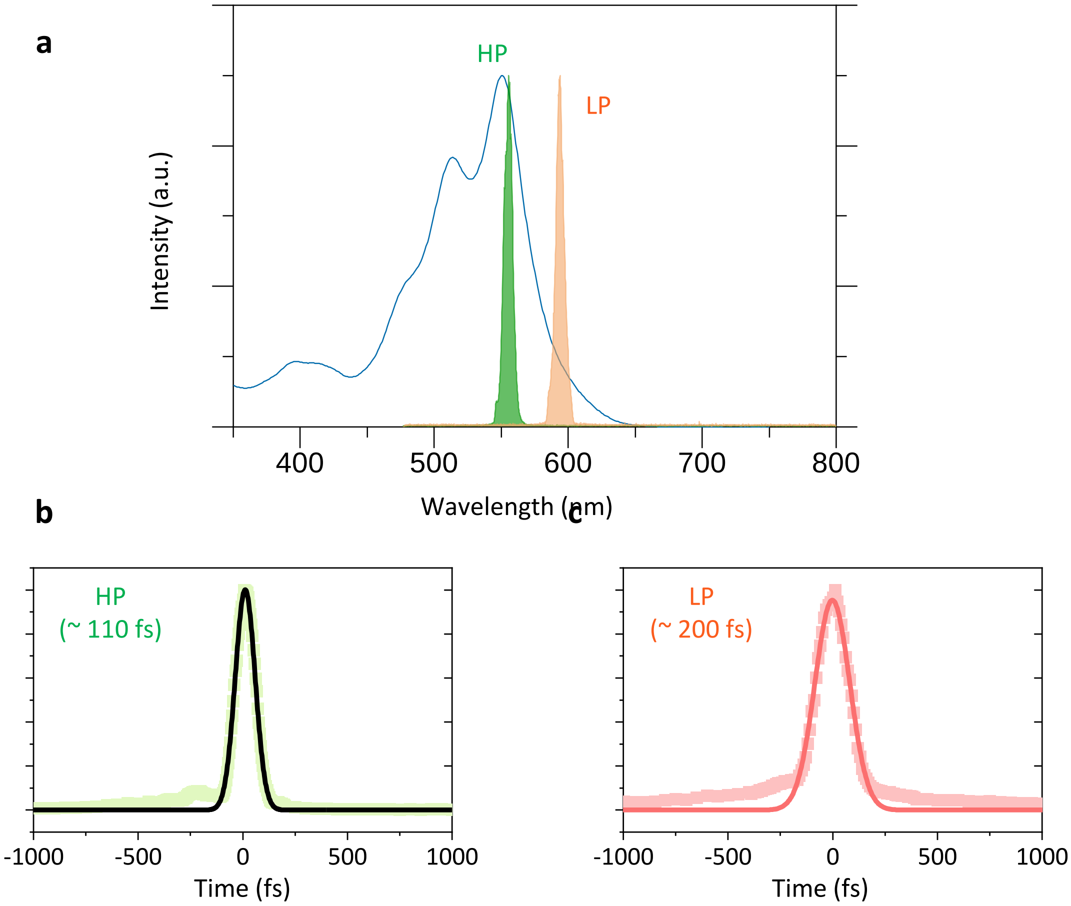

Supplementary Figure 25. The characterization of OPO pulses for fs-TF experiments. **a**, the steady-state absorption (black line) of **Tirs-PBI** in TOL and the OPO pulses for HP (green area) and LP (yellow area). **b**,**c**, the instrumental response function (IRF) for HP (**b**) and LP (**c**).

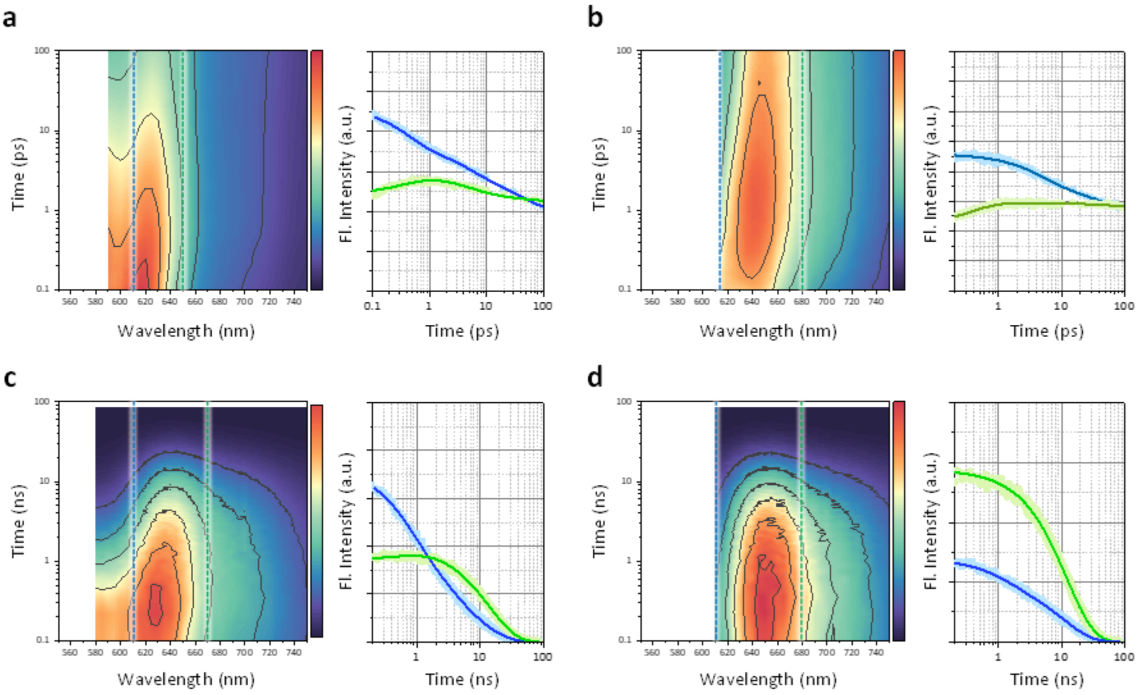

Supplementary Figure 26. The *fs*-to-*ns* TFS of **Tris-PBI** in TOL upon HP and LP. **a**,**b**, the 2D contour map (left) and decay profiles (right) of **Tris-PBI** upon HP (**a**) and LP (**b**) in the range of 0.1 – 100 ps. The scale bar indicates the relative fluorescence intensity.The dashed line indicates the respective wavelengths for fitting decay profiles. **c**,**d**, the 2D contour map (left) and decay profiles (right) of **Tris-PBI** in toluene by the HP (**c**) and LP (**d**) in the range of 0.1 – 90 ns. The dashed lines indicate the respective wavelengths for fitting decay profiles.

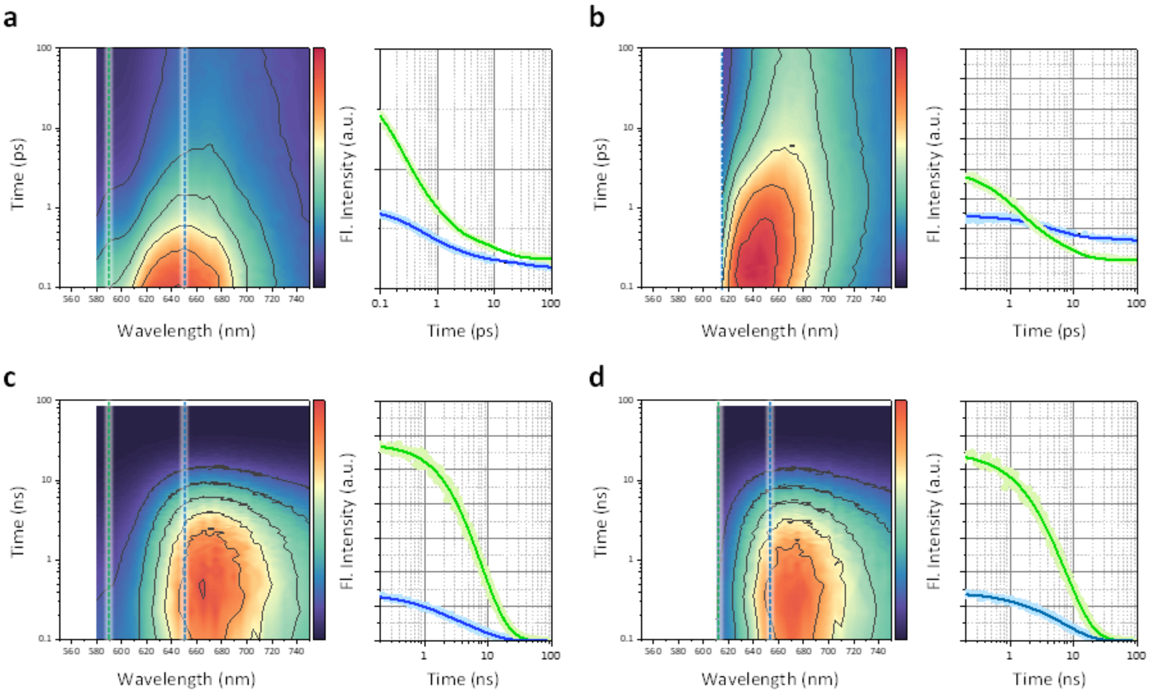

Supplementary Figure 27. The *fs*-to-*ns* TFS of **Tris-PBI** in THF upon HP and LP. **a**,**b**, the 2D contour map (left) and decay profiles (right) of **Tris-PBI** upon HP (**a**) and LP (**b**) in the range of 0.1 – 100 ps. The dashed line indicates the respective wavelengths for fitting decay profiles. **c**,**d**, the 2D contour map (left) and decay profiles (right) of **Tris-PBI** in THF by HP (**c**) and LP (**d**) in the range of 0.1 – 90 ns. The dashed lines indicate the respective wavelengths for fitting decay profiles.

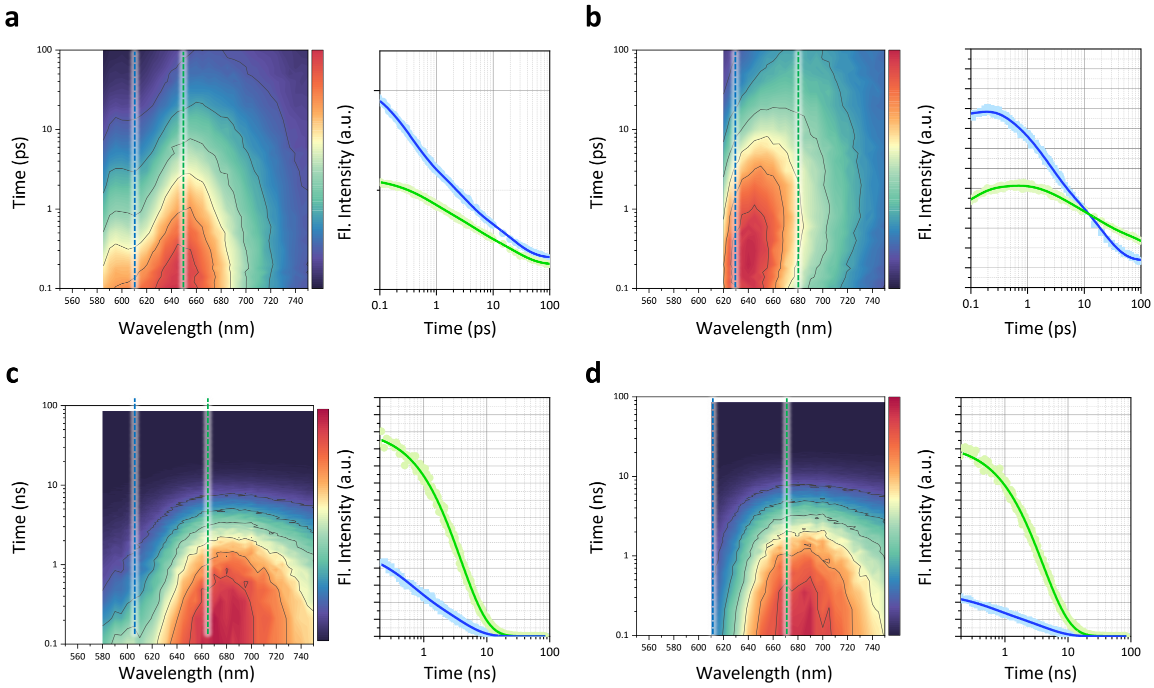

Supplementary Figure 28. The *fs*-to-*ns* TFS of **Tris-PBI** in BCN upon HP and LP. **a**,**b**, The 2D contour map (left) and decay profiles (right) of **Tris-PBI** upon HP (**a**) and LP (**b**) in the range of 0.1 – 100 ps. The dashed line indicates the respective wavelengths for fitting decay profiles. **c**,**d**, The 2D contour map (left) and decay profiles (right) of **Tris-PBI** in BCN by HP (**c**) and LP (**d**) in the range of 0.1 – 90 ns. The dashed lines indicate the respective wavelengths for fitting decay profiles.

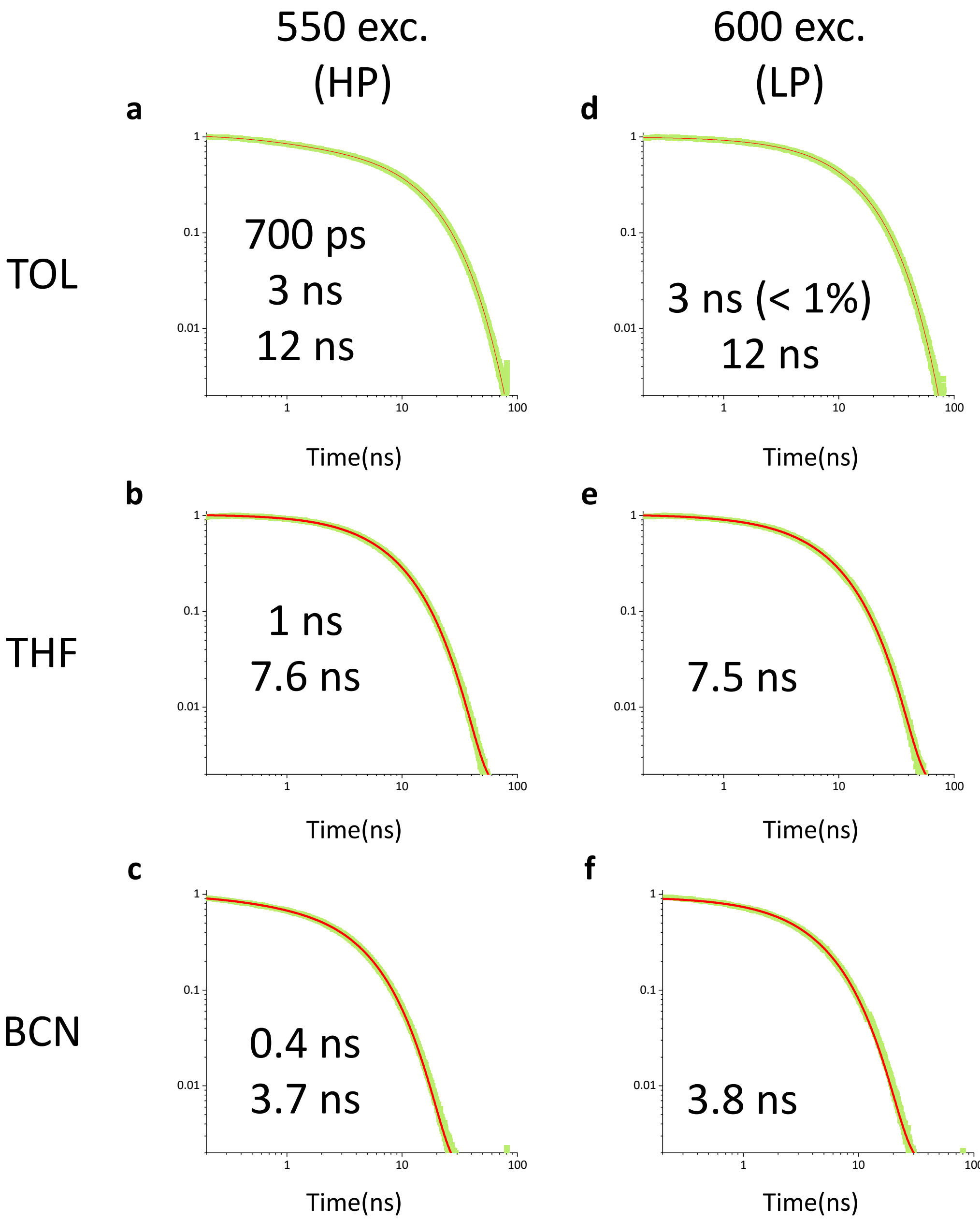

Supplementary Figure 29. The averaged decay profiles of *ns*-TFS of **Tris-PBI** by HP (**a**-**c**) and LP (**d**-**f**).

Time-resolved vibrational spectroscopy

Herein, we used three pulse schemes (Supplementary Figure 30). The first pulse is an actinic pump (P1; 550 and 595 nm for HP and LP, ~ 180 fs), which generates the excited state population. The second pulse is near-infrared Raman pump (P2, sub-10-fs), which generates the excited-state vibrational wavepackets after an arbitrary time delay of ΔT between P1 and P2 pulses. The final pulse is near-infrared Raman probe (P3; a replica of P2, sub-10-fs), which records P2-induced differential absorption signals with a time delay τ. To investigate the role of CT state, the sub-10-fs P2 pulse is tuned to be resonant with the ESA bands of ME state in range of 700-900 nm. The excited-state Raman spectra are obtained by Fourier transformation (FT) of oscillatory residuals extracted by subtraction of population kinetics with multiexponential fitting. Note that post-processing procedures, such as zero padding and apodization (hanning window), were used to improve the spectral quality.

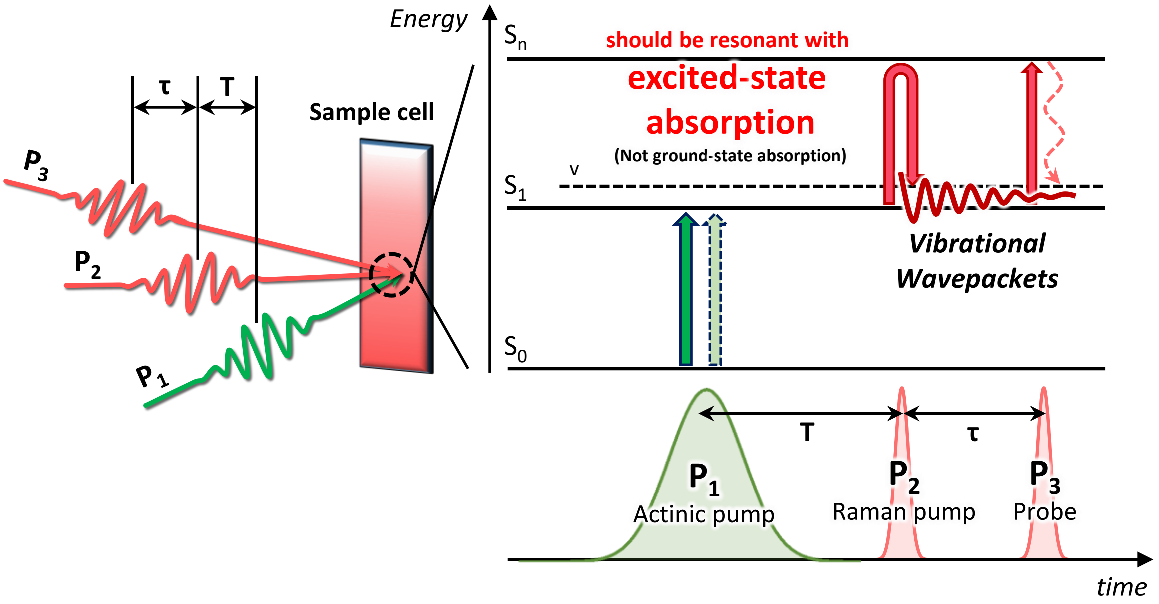

Supplementary Figure 30. The experimental schemes for TR-ISRS.

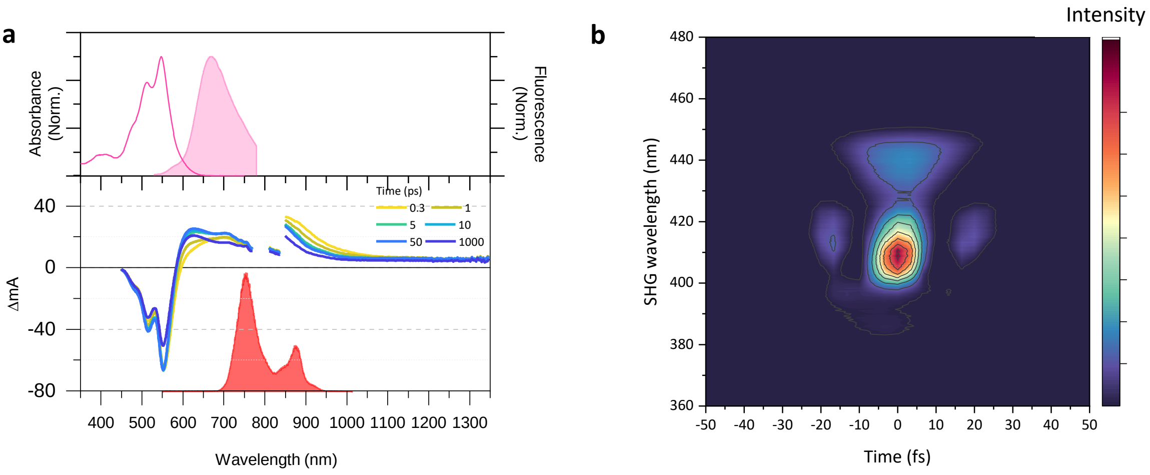

Supplementary Figure 31. The pulse characterization. **a**, the steady-state absorption and fluorescence spectra (top), the TA spectra (bottom), and our home-built noncollinear optical parametric amplifier (NIR-NOPA) spectrum (bottom, red area). The NIR NOPA covers the ESA band corresponding to the ME state, consisting of LE, radical anion (CT), TT configurations. **b**, the 2D contour map for second harmonic generation frequency-resolved optical gating (SHG-FROG) measurement.

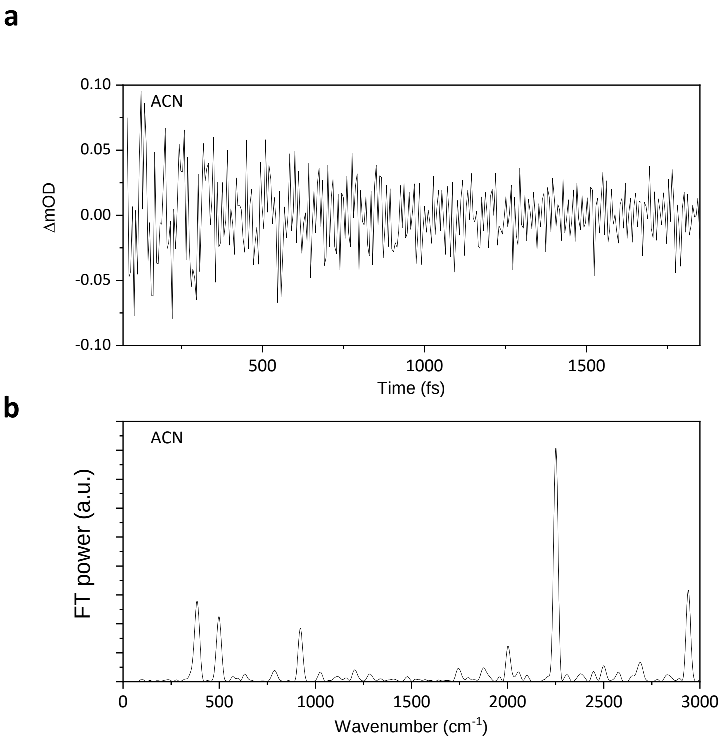

Supplementary Figure 32. The non-resonant ISRS measurement of an acetonitrile using a band pass filter above 850 nm. **a**, the oscillatory component generated through Raman pump. **b**, the FT power spectrum of an acetonitrile. The vibrational mode at 2944 cm^{-1} indicates that the temporal resolution of our NOPA is sub-10 fs.

a

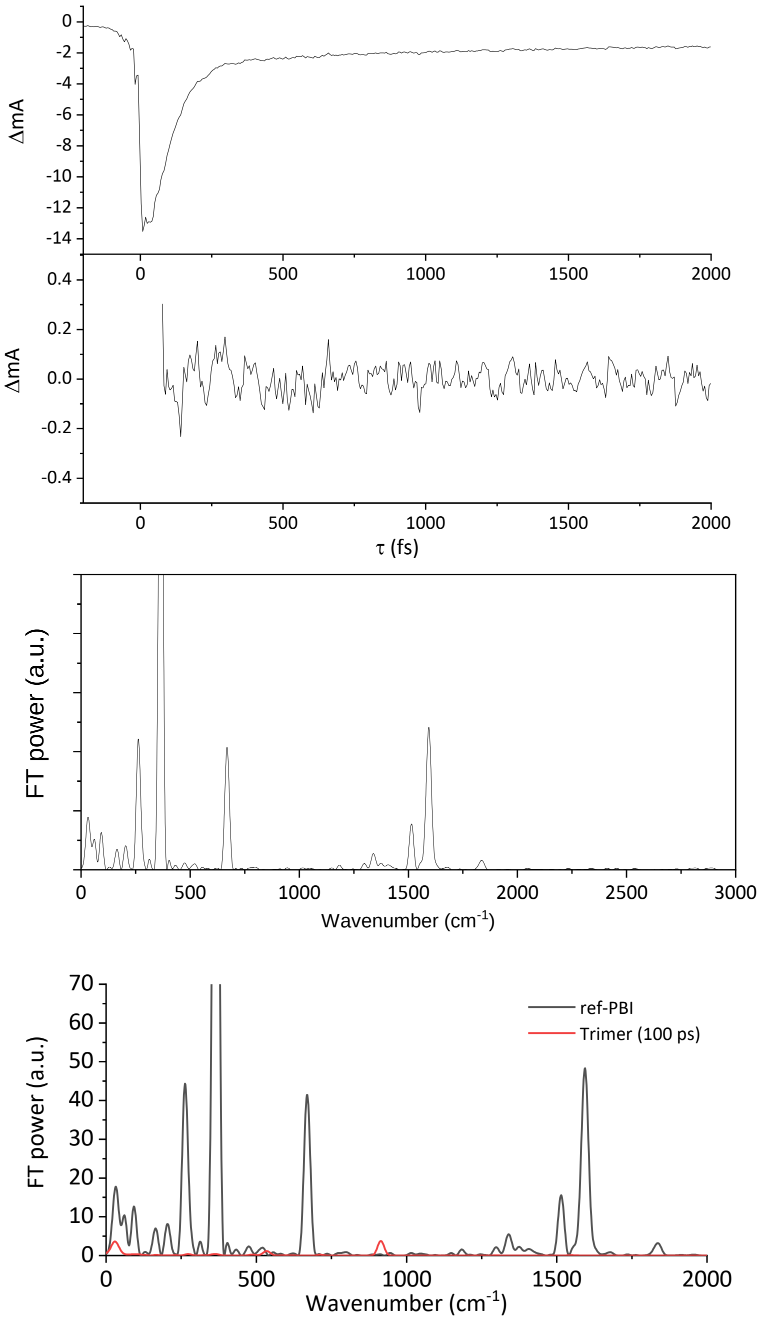

Supplementary Figure 33. The resonant ISRS measurement of **Ref-PBI** in chloroform. **a**, the raw TR-ISRS signal (top) and the residual component after the subtraction of the population dynamics (bottom). **b**, The FT power spectrum of **Ref-PBI** (ΔT=10 ps). **c**, A direct comparison between **ref-PBI** (black) and **Tris-PBI** (red) in the same experimental condition. This shows that the excited state Raman intensity of **Tris-PBI** is highly suppressed compared to that of **Ref-PBI**. The low signal intensity hinders a qualitative analysis of TR-ISRS results.

Supplementary Note 4

Here we revisit the previously reported monomer and dimer (**Ref-PBI** and **Bis-PBI2**) data which represent the LE and multiexciton states.[S5] Supplementary Figure 34c shows that the initial excited species (FE) of **Bis-PBI2** proceeds to the ME (LE+CR+TT) state with the time constant of 1 and 17 ps. The high-frequency region in FT power spectra manifest the striking change along with the evolution of the FE into ME state. As shown in Figure 5a, the FE state indicates the initial C=C stretch mode at 1595 cm^{-1}. Subsequently, the C=C stretch mode disappeared and the newly generated C=C stretch mode at 1545 cm^{-1} becomes prominent, which corresponds to the CR configuration (PBI anionic band). In addition, when the ME state is evolved, the distinct rise of the modes at 480 and 550 cm^{-1} (ring breathing and ring deformation modes, respectively) is observed. Considering that these modes are associated with perturbation of the PBI aromatic rings, this result suggests that RB and RD modes lead to fluctuation of the orbital interaction which contribute to the efficient coupling between FE and ME states. In conclusion, Figure 5a in main text shows the TR-ISRS spectra in the distinctive fingerprint region for the respective states of previously reported dimers (the multiexciton, **Bis-PBI2**): 1) low-frequency modes for the excimer state (interchromophoric out-of-plane mode, xOOP mode). 2) 480 cm-1 for CR-enhanced state (ring breathing mode, RB) 3) 580 cm^{-1} for the CT-enhanced state (Ring deformation mode, RD). 4) 1545 cm^{-1} for the CT-related state (C=C stretch mode, C=C_{CR}). 5) 1595 cm^{-1} for the LE state (C=C stretch mode, C=C_{LE}).

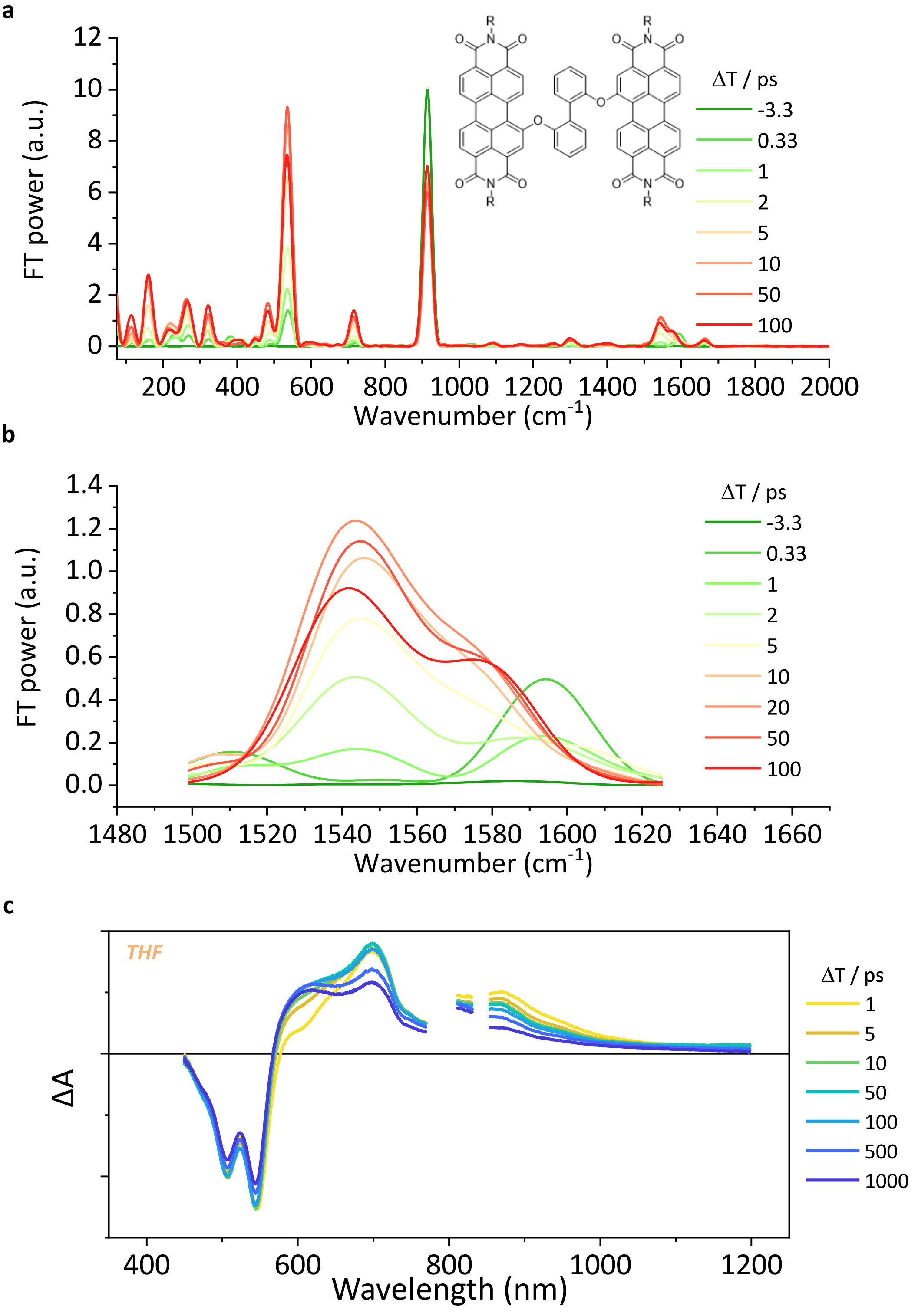

Supplementary Figure 34. The excited-state Raman spectra of **Bis-PBI 2** in THF.[S5] **a**, the representative excited-state Raman spectra.The inset indicates the chemical structure of **Bis-PBI 2**. **b**, The enlargement of high-frequency region. (c) the representative TA spectra of **Bis-PBI 2** in THF. Supplentary Rererence S7 indicates that the MEG process of **Bis-PBI 2** in THF occurs with 1 and 17 ps. Therefore, we assign the iniital state is LE state (C=C stretch mode at 1595 cm^{-1}). Subsequently, at ΔT= 50 ps (ME state in the main text), the modes at 490, 535, and 1545 cm^{-1} become prominent, suggesting that these modes are the fingerprint region for the CR configurations.

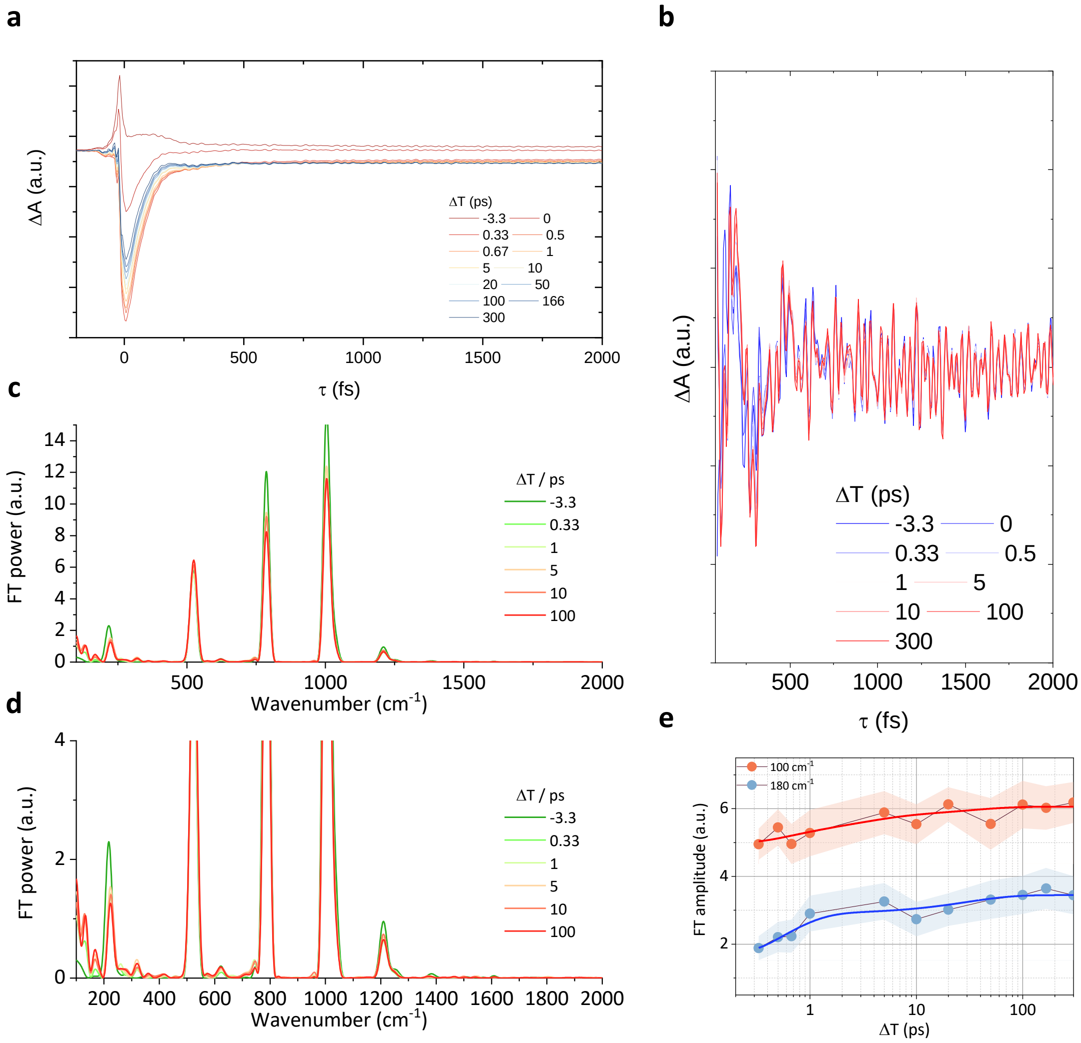

Supplementary Figure 35. TR-ISRS results of Tris-PBI in TOL by HP. a,**b**, raw TR-ISRS kinetic profiles (**a**) and oscillatory residuals (**b**) extracted from multiexponential fits to the kinetic traces at each T for **Tris-PBI** in TOL. **c**,**d**, the representative excited-state Raman spectra. **e**, the temporal trace for the FT amplitude at around 100 and 180 cm^{-1}.

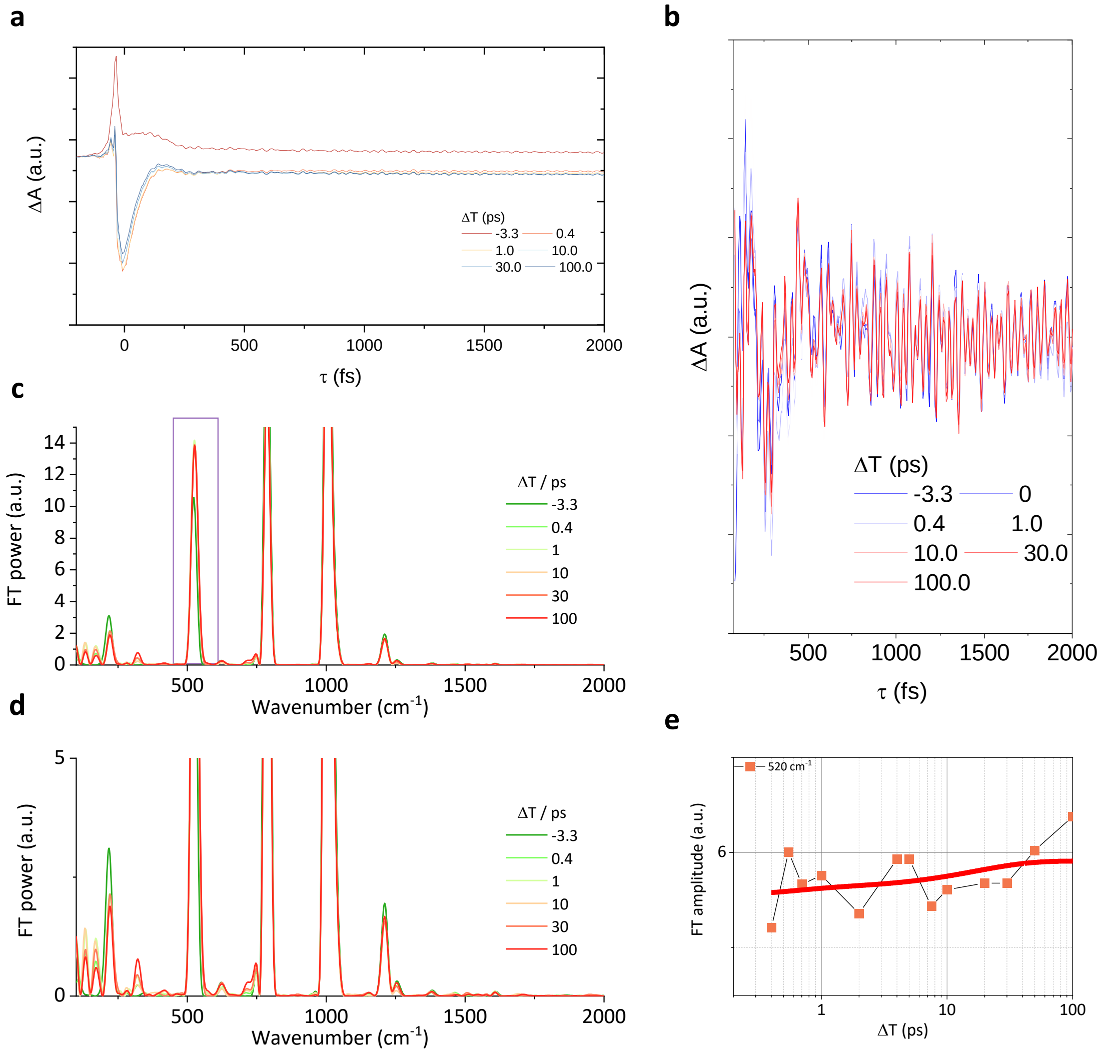

Supplementary Figure 36. TR-ISRS results of Tris-PBI in TOL by LP. a,**b**, raw TR-ISRS kinetic profiles (**a**) and oscillatory residuals (**b**) extracted from multiexponential fits to the kinetic traces at each T for **Tris-PBI** in TOL. **c**,**d**, the representative excited-state Raman spectra. **e**, the temporal trace for the FT amplitude at around 530 cm^{-1}. Due to the interference with solvent signal, the teporal traces show large amount of noise.

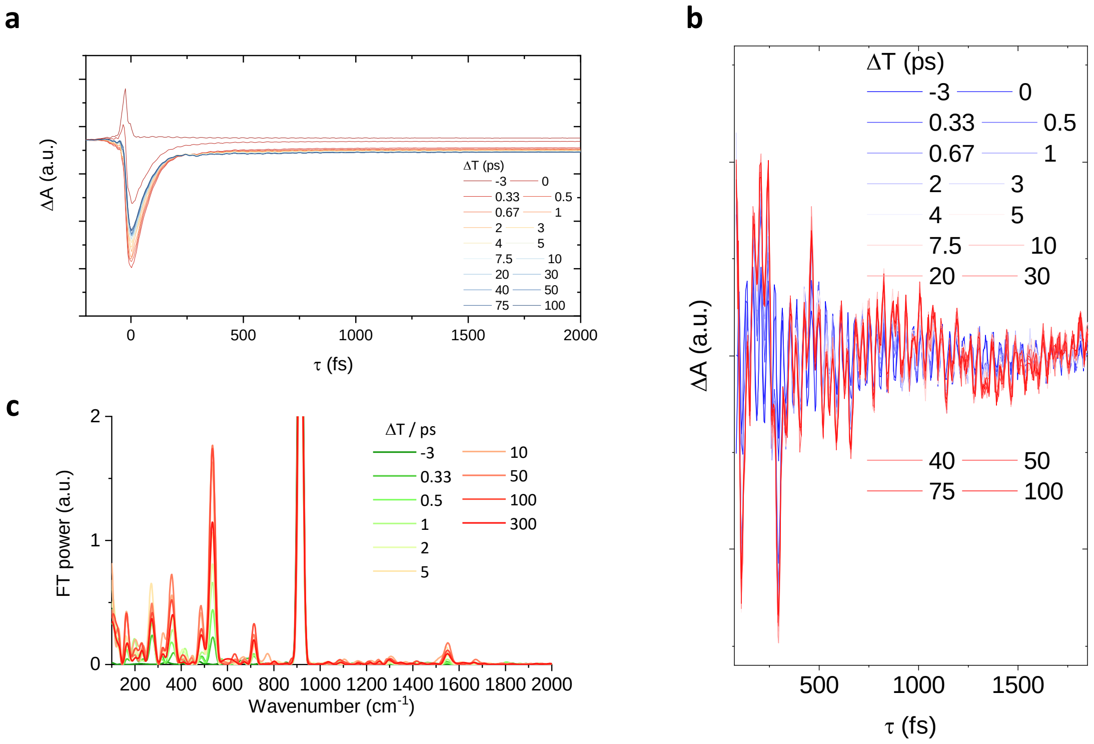

Supplementary Figure 37. TR-ISRS results of Tris-PBI in THF upon HP. a,**b**, raw TR-ISRS kinetic profiles (**a**) and oscillatory residuals (**b**) extracted from multiexponential fits to the kinetic traces at each T for **Tris-PBI** in THF. **c**, the representative excited-state Raman spectra.

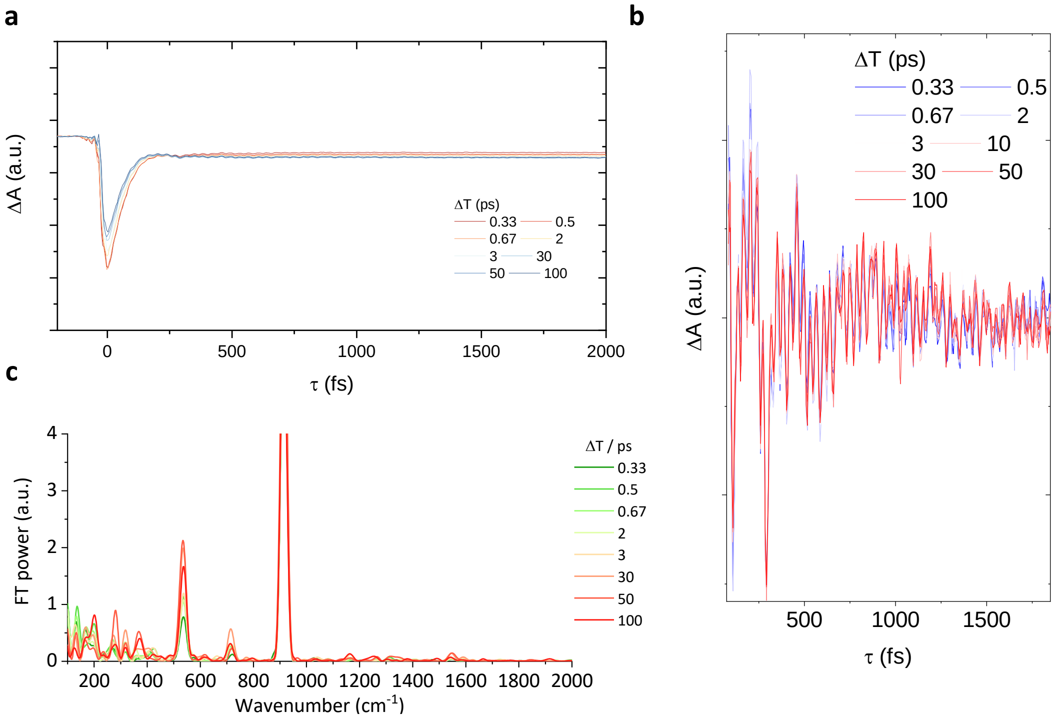

Supplementary Figure 38. TR-ISRS results of Tris-PBI in THF by LP. **a**,**b**, raw TR-ISRS kinetic profiles (**a**) and oscillatory residuals (**b**) extracted from multiexponential fits to the kinetic traces at each T for **Tris-PBI** in THF. **c**, the representative excited-state Raman spectra.

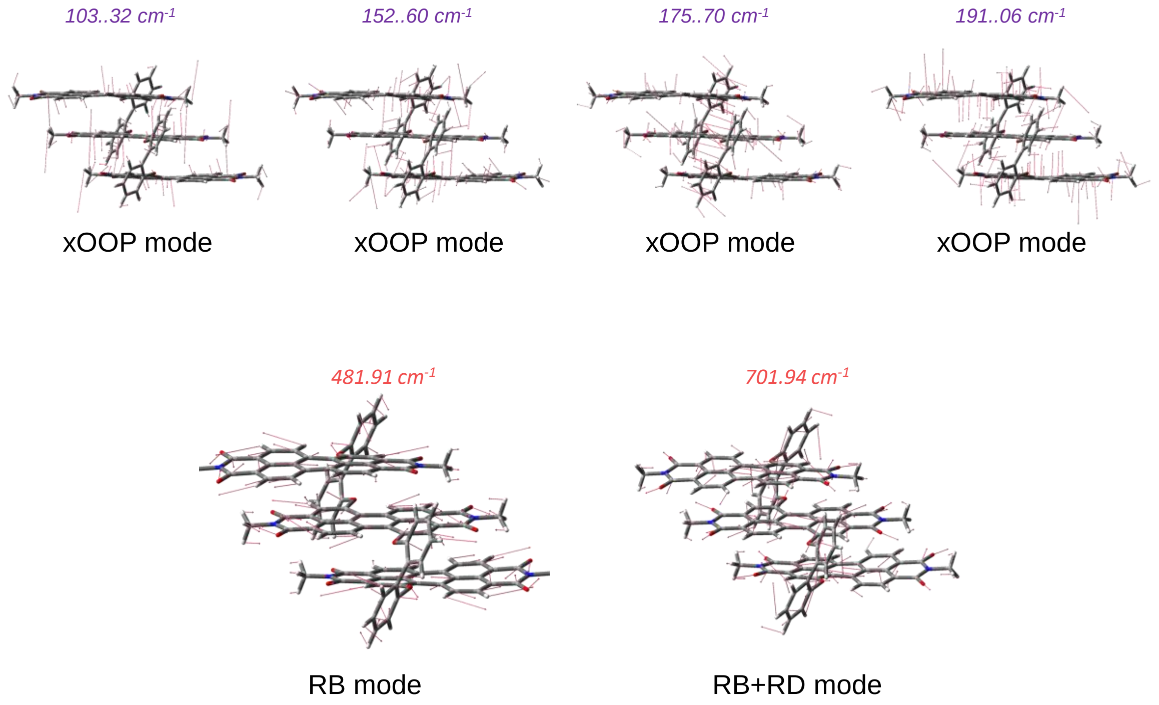

Supplementary Figure 39. The displacement vectors for the representative vibrational modes of **Tris-PBI** (neutral).

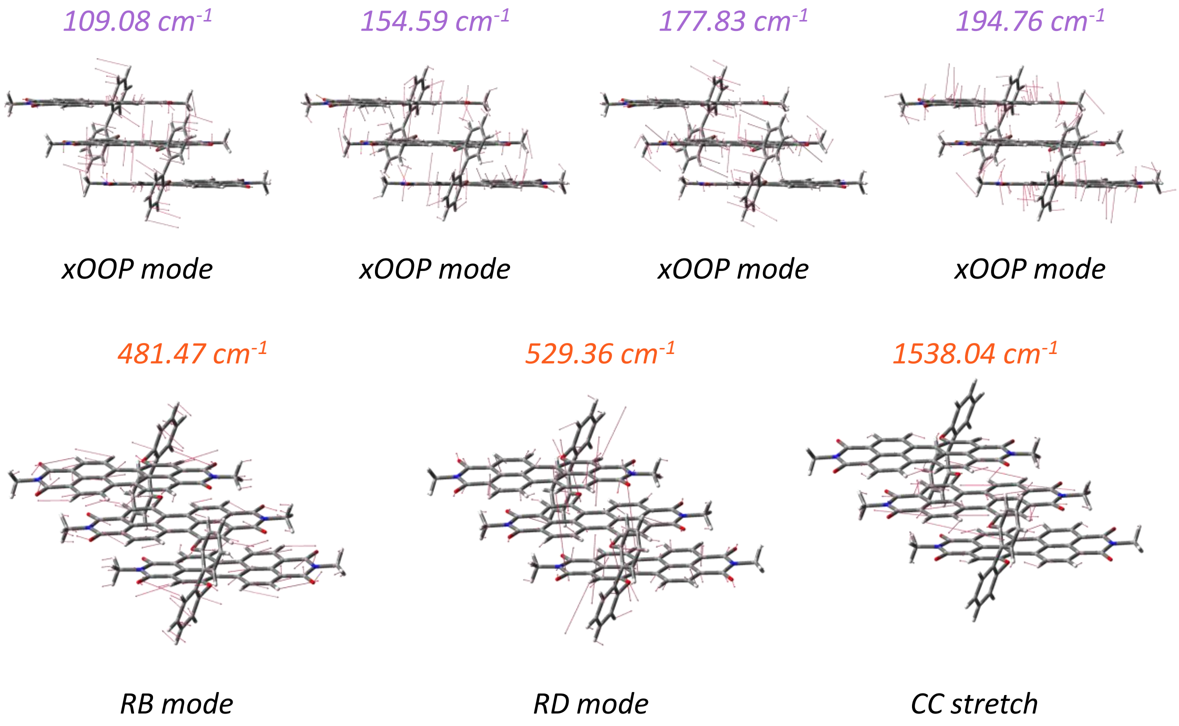

Supplementary Figure 40. The displacement vectors for the representative vibrational modes of **Tris-PBI** (anion). The negligible chane in peak poistion of excited-state Raman spectra in THF indicates that not the initial species of **Tris-PBI** is sufficiently mixed with CT state but the initial speceis proceeds to an admixture of LE, CR, TT states (ME state) rather than pure CT or CS state. Here, we neglect the radical cation since the PBI cation is observed in the ESA band at around 580 nm.[S5]

Quantum chemical simulations

Exciton coupling calculation

Supplementary Table 5. Geometrical parameters of the close and far dimers of **Tris-PBI** in its single crystal as well as the calculated exciton coupling contributions J_{Coul} and J_{CT} between these dimers compared to those of **Bis-PBI2**.

**Parameter**	**Centroid distance [Å]**	**π-π-distance [Å]**	**Rotation [°]**	**Slip angle [°]**
Far dimer	9.33	6.86	0	47
Close dimer	4.67	3.41	9	47

**Coupling [cm^{-1} (meV)]**	J_{Coul}	t_{e}	t_{h}	J_{CT}	J_{total}	$J_{\mathrm{total;UV\text{-}Vis}}$
**Tris-PBI** – Far	−78 (−10)	6 (1)	2 (0)	−0 (−0)	−78 (−10)	89 (11)
**Tris-PBI** – Close	470 (58)	504 (62)	792 (98)	−499 (−61)	−29 (−3)	
**Bis-PBI2**[S17]	554 (69)	697 (86)	547 (68)	−477 (−59)	77 (10)	152 (19)

Supplementary Table 6. Vertical excitation energies of the **Tris-PBI** crystal structure at the TDDFT (ωB 97XD/def2SVP) level.

**State**	**Benzonitrile (BCN)**		**Tetrachloroethane (TCE)**		**Toluene (TOL)**	
	**Energy [eV]**	**Osc. strength**	**Energy [eV]**	**Osc. strength**	**Energy [eV]**	**Osc. strength**
S_1	2.2361	0.4575	2.2525	0.3554	2.2525	0.4304
S_2	2.3874	0.0000	2.4049	0.0000	2.3885	0.0000
S_3	2.3996	1.3261	2.4200	1.2102	2.4017	1.2146
S_4	2.7283	0.0000	2.7269	0.0000	2.7143	0.0000
S_5	2.7606	0.1946	2.7604	0.2386	2.7500	0.3466

RAS-SF simulation

We performed restricted active space with spin-flip (RAS-SF) simulations for the structure obtained from the DFT calculations (CAM-B3LYP/6-31G(d), the Cartesian coordinate is provided below). It is necessary to include all the possible combinations of TT states that can form on three PBIs for the balanced SF process description, which lead us to use RAS with triple spin-flip (RAS-3SF). The reference state was generated from a restricted open-shell Hartree-Fock calculation with six electrons in six orbitals; septet state. The double-zeta quality basis sets, 6-31(G), were used. Adiabatic wavefunction decomposition was carried out for the RAS-3SF states, and **Tris-PBI** is divided into three PBI monomers.[S36] The RAS-3SF calculations and the related analysis were conducted using Q-Chem 5.1.[S37]

Supplementary Table 7. Excitation energies (in eV) and adiabatic wavefunction composition (%) of MEG-relevant states in the ground state (S_0) geometry. (GS: ground state, LE: local exciton, ME: multiexciton state, CR: charge resonance, and Sn: the nth singlet excited state).

State	Energy (eV)	GS (%)	LE (%)	ME (SS+TT) (%)	SS (%)	TT (%)	CR (%)
S_1[a]	3.3247	1%	8%	25%	24%	1%	66%
S_2[a]	3.3559	1%	16%	16%	13%	3%	67%
S_3[b]	3.4175	6%	20%	47%	45%	2%	27%
S_4	3.6299	0%	23%	25%	18%	7%	52%
S_5[b]	3.6854	6%	6%	35%	25%	10%	53%
S_6	4.0127	0%	26%	18%	16%	2%	56%
S_7[c]	4.1536	0%	0%	80%	14%	66%	20%
S_8	4.3006	0%	2%	73%	37%	36%	25%
S_9	4.3059	8%	8%	54%			30%

a. The lowest transition consists of mainly CT character. (S1 and S2 are nearly resonant)

b. The second and third allowed transition are S3 and S5, respectively. Considering the energy difference of pump laser (550 nm and 600 nm) which is about ~0.2 eV, both of them could correspond to LE excitation in the manuscript, and they contain less CT character than S1 and S2.

c. The ME state is S7. This has more than 66 % contribution of TT diabat.

Cartesian coordinates of optimized Tris-PBI geometry using B97D3/def2SVP (in Å)

N	4.19576500	-8.36904100	-0.29638800
C	3.86330600	-7.95468300	0.99811400
C	2.77139600	-6.95383600	1.11170700
C	2.07295600	-6.49175200	-0.02821300
C	2.43296600	-6.98541400	-1.30358900
C	3.54999000	-7.95188100	-1.45866900
O	3.90930300	-8.36347600	-2.54056300
O	4.44575400	-8.38755400	1.96700000
C	2.43912600	-6.46198700	2.35093700
C	1.43784900	-5.49057700	2.49180100
C	0.73149900	-4.99107600	1.40093300
C	1.03503300	-5.52636200	0.10402700
C	0.34033900	-5.11012100	-1.07122400
C	0.72567900	-5.62070200	-2.30421400
C	1.76890800	-6.54471000	-2.42691600
C	-0.75969400	-4.14134600	-0.94206300
C	-1.04089500	-3.56694200	0.33689900
C	-0.29956500	-3.93590000	1.51132700
C	-0.58351900	-3.23322200	2.69125100
C	-1.59893100	-2.25931000	2.74859000
C	-2.34586900	-1.95723800	1.64301700
C	-2.07188900	-2.59056500	0.41309200
C	-2.81501700	-2.21675300	-0.72906800
C	-2.54185900	-2.80534900	-1.94394600
C	-1.51799500	-3.75385500	-2.04011000
C	-3.88978300	-1.19842300	-0.63764500
N	-4.10527500	-0.60303100	0.60060300
C	-3.41283700	-0.93065000	1.76830700
O	-3.67640000	-0.39767800	2.82277400
O	-4.56576400	-0.88046800	-1.59649700
C	-5.15762500	0.40059500	0.68946700
C	5.28830200	-9.31677300	-0.46953500
H	4.92160000	-10.23150400	-0.95413400
H	6.06526300	-8.87963100	-1.11088600
H	5.69421200	-9.54742200	0.51936700
H	-5.18113600	0.97725500	-0.24148100
H	-6.13869500	-0.07645700	0.83049600
H	-4.94989300	1.04340300	1.55083600

H	2.07862400	-6.91614700	-3.40432100
H	0.23449800	-5.28856400	-3.21692900
H	-1.32117500	-4.18902200	-3.01817900
H	-3.12177800	-2.51571700	-2.82135000
H	2.98282800	-6.82539900	3.22394900
H	1.23270900	-5.11744600	3.48646300
H	-1.79428600	-1.71212200	3.66986100
O	0.18435100	-3.43616700	3.79471800
C	-0.28472000	-3.08440800	5.03756700
C	0.50090700	-2.26696900	5.86004900
C	-0.02812200	-1.94224800	7.12171600
C	-1.25947000	-2.41930500	7.55380800
C	-1.99700700	-3.26870700	6.73061400
C	-1.50378800	-3.60430500	5.47685000
H	-2.06604900	-4.26469900	4.81379700
H	-2.95681500	-3.66940200	7.06143200
H	-1.64004200	-2.13286200	8.53564700
H	0.55212100	-1.28437300	7.77202900
C	1.88403400	-1.80703900	5.54342800
C	2.87312100	-2.01218800	6.52151400
C	4.17256700	-1.54533300	6.36852700
C	4.51413100	-0.83103700	5.22189800
C	3.56178900	-0.61182500	4.23460600
C	2.27012800	-1.11844000	4.38470100
H	2.60419400	-2.57016700	7.42087100
H	4.91500200	-1.73722000	7.14511100
H	5.52474600	-0.44164300	5.08640000
H	3.82679900	-0.05844900	3.33067100
O	1.32947700	-0.86995500	3.41071900
N	4.00278700	-3.93554600	-1.06196600
C	3.81456900	-3.53937000	0.25811500
C	2.76211200	-2.51992300	0.51302500
C	1.99112100	-1.97684900	-0.53531600
C	2.21560900	-2.44750800	-1.84748500
C	3.26742200	-3.45032200	-2.14699900
O	3.48655900	-3.84125800	-3.27205600
O	4.49235900	-4.00163400	1.15058000
C	2.54345900	-2.12614400	1.80380200
C	1.57426800	-1.15336700	2.10276900
C	0.80397400	-0.54328300	1.09673200

C	1.00145400	-0.99126300	-0.25431400
C	0.21724200	-0.49100200	-1.35021700
C	0.45574900	-1.01193100	-2.62124500
C	1.44412700	-1.96644300	-2.87497100
C	-0.80397400	0.54328300	-1.09673200
C	-1.00145400	0.99126300	0.25431400
C	-0.21724200	0.49100200	1.35021700
C	-0.45574900	1.01193100	2.62124500
C	-1.44412700	1.96644300	2.87497100
C	-2.21560900	2.44750800	1.84748500
C	-1.99112100	1.97684900	0.53531600
C	-2.76211200	2.51992300	-0.51302500
C	-2.54345900	2.12614400	-1.80380200
C	-1.57426800	1.15336700	-2.10276900
C	-3.81456900	3.53937000	-0.25811500
N	-4.00278700	3.93554600	1.06196600
C	-3.26742200	3.45032200	2.14699900
O	-3.48655900	3.84125800	3.27205600
O	-4.49235900	4.00163400	-1.15058000
C	-5.05450700	4.90764100	1.32413200
H	-5.09245800	5.62321800	0.49548300
H	-6.03324600	4.41012900	1.39589700
H	-4.83627100	5.41219300	2.27048700
C	5.05450700	-4.90764100	-1.32413200
H	4.83627100	-5.41219300	-2.27048700
H	6.03324600	-4.41012900	-1.39589700
H	5.09245800	-5.62321800	-0.49548300
H	1.62018100	-2.33110700	-3.88740600
H	-0.12833000	-0.66036500	-3.45947300
H	-3.12133000	2.59955500	-2.59625200
H	3.12133000	-2.59955500	2.59625200
H	0.12833000	0.66036500	3.45947300
H	-1.62018100	2.33110700	3.88740600
O	-1.32947700	0.86995500	-3.41071900
C	-2.27012800	1.11844000	-4.38470100
C	-1.88403400	1.80703900	-5.54342800
C	-2.87312100	2.01218800	-6.52151400
C	-4.17256700	1.54533300	-6.36852700
C	-4.51413100	0.83103700	-5.22189800
C	-3.56178900	0.61182500	-4.23460600

H	-3.82679900	0.05844900	-3.33067100
H	-5.52474600	0.44164300	-5.08640000
H	-4.91500200	1.73722000	-7.14511100
H	-2.60419400	2.57016700	-7.42087100
C	-0.50090700	2.26696900	-5.86004900
C	0.02812200	1.94224800	-7.12171600
C	1.25947000	2.41930500	-7.55380800
C	1.99700700	3.26870700	-6.73061400
C	1.50378800	3.60430500	-5.47685000
C	0.28472000	3.08440800	-5.03756700
H	-0.55212100	1.28437300	-7.77202900
H	1.64004200	2.13286200	-8.53564700
H	2.95681500	3.66940200	-7.06143200
H	2.06604900	4.26469900	-4.81379700
N	4.10527500	0.60303100	-0.60060300
C	3.88978300	1.19842300	0.63764500
C	2.81501700	2.21675300	0.72906800
C	2.07188900	2.59056500	-0.41309200
C	2.34586900	1.95723800	-1.64301700
C	3.41283700	0.93065000	-1.76830700
O	3.67640000	0.39767800	-2.82277400
O	4.56576400	0.88046800	1.59649700
C	2.54185900	2.80534900	1.94394600
C	1.51799500	3.75385500	2.04011000
C	0.75969400	4.14134600	0.94206300
C	1.04089500	3.56694200	-0.33689900
C	0.29956500	3.93590000	-1.51132700
C	0.58351900	3.23322200	-2.69125100
C	1.59893100	2.25931000	-2.74859000
C	-0.73149900	4.99107600	-1.40093300
C	-1.03503300	5.52636200	-0.10402700
C	-0.34033900	5.11012100	1.07122400
C	-0.72567900	5.62070200	2.30421400
C	-1.76890800	6.54471000	2.42691600
C	-2.43296600	6.98541400	1.30358900
C	-2.07295600	6.49175200	0.02821300
C	-2.77139600	6.95383600	-1.11170700
C	-2.43912600	6.46198700	-2.35093700
C	-1.43784900	5.49057700	-2.49180100
C	-3.86330600	7.95468300	-0.99811400

N	-4.19576500	8.36904100	0.29638800
C	-3.54999000	7.95188100	1.45866900
O	-3.90930300	8.36347600	2.54056300
O	-4.44575400	8.38755400	-1.96700000
C	-5.28830200	9.31677300	0.46953500
H	-5.69421200	9.54742200	-0.51936700
H	-6.06526300	8.87963100	1.11088600
H	-4.92160000	10.23150400	0.95413400
C	5.15762500	-0.40059500	-0.68946700
H	4.94989300	-1.04340300	-1.55083600
H	6.13869500	0.07645700	-0.83049600
H	5.18113600	-0.97725500	0.24148100
H	1.79428600	1.71212200	-3.66986100
O	-0.18435100	3.43616700	-3.79471800
H	-1.23270900	5.11744600	-3.48646300
H	-2.98282800	6.82539900	-3.22394900
H	3.12177800	2.51571700	2.82135000
H	1.32117500	4.18902200	3.01817900
H	-0.23449800	5.28856400	3.21692900
H	-2.07862400	6.91614700	3.40432100

Cartesian coordinates of the optimized Tris-PBI geometry using CAM-B3LYP-D/6-31G(d)(in Å)

O	4.05200	-8.27000	-2.54900
O	4.42300	-8.41700	1.97700
O	-4.58700	-1.00000	-1.70300
O	-3.67400	-0.41900	2.70600
O	0.16000	-3.48000	3.72900
N	4.25100	-8.33700	-0.29500
N	-4.12500	-0.68900	0.49100
C	3.63800	-7.89800	-1.46500
C	2.49800	-6.96700	-1.32500
C	1.86700	-6.50900	-2.45600
H	2.22100	-6.84100	-3.42400
C	0.80000	-5.61600	-2.34600
H	0.34000	-5.26500	-3.25900
C	0.36000	-5.15200	-1.11600
C	-0.76900	-4.22200	-0.99700
C	-1.53300	-3.86300	-2.09700
H	-1.33500	-4.31000	-3.06100
C	-2.56500	-2.92800	-2.01100
H	-3.14600	-2.65200	-2.88300
C	-2.84100	-2.32600	-0.80700
C	-3.91000	-1.30800	-0.73400
C	-3.42700	-0.99100	1.66200
C	-2.37700	-2.03200	1.56000
C	-1.63200	-2.31700	2.66800
H	-1.81700	-1.75800	3.57500
C	-0.62400	-3.29300	2.62200
C	-0.33700	-4.00900	1.45700
C	0.68900	-5.06600	1.36200
C	1.35600	-5.58700	2.46400
H	1.11100	-5.24300	3.45300
C	2.37000	-6.54200	2.33300
H	2.88400	-6.92200	3.20800
C	2.75400	-6.99100	1.09700
C	3.86400	-7.96500	0.99600
C	2.08800	-6.51500	-0.05300
C	1.03200	-5.57400	0.06800
C	-1.06800	-3.64900	0.27600
C	-2.10300	-2.68000	0.34000

C	-0.32900	-3.15100	4.98000
C	-1.55000	-3.67600	5.39100
H	-2.10800	-4.30200	4.70400
C	-2.04500	-3.38500	6.65100
H	-3.00300	-3.78800	6.96100
C	-1.30500	-2.57800	7.50700
H	-1.68400	-2.32900	8.49200
C	-0.07400	-2.09200	7.09800
H	0.50300	-1.46300	7.76700
C	0.45700	-2.37000	5.83000
C	5.37000	-9.26100	-0.46000
H	5.03300	-10.16100	-0.97600
H	5.74900	-9.50500	0.52800
C	-5.17500	0.32500	0.54900
H	-5.12900	0.93400	-0.35100
H	-5.01600	0.92500	1.44000
H	6.14500	-8.79100	-1.06700
H	-6.15800	-0.14900	0.59900
O	3.50500	-3.79000	-3.29900
O	4.47600	-4.01500	1.13400
O	1.26400	-0.94400	3.40800
N	4.02200	-3.89500	-1.08700
C	3.28600	-3.40200	-2.16700
C	2.23600	-2.40600	-1.86300
C	1.46500	-1.92200	-2.88500
H	1.64600	-2.27700	-3.89300
C	0.47100	-0.97900	-2.62800
H	-0.11200	-0.63000	-3.45800
C	0.22900	-0.47000	-1.35500
C	1.00600	-0.98100	-0.26200
C	0.79100	-0.55900	1.09300
C	1.54300	-1.19400	2.09100
C	2.51400	-2.15800	1.79200
H	3.07100	-2.64600	2.58000
C	2.75700	-2.51800	0.49900
C	3.81100	-3.52900	0.23700
C	2.00100	-1.95700	-0.54800
C	1.83400	-1.88900	5.53100
C	2.81800	-2.08000	6.51100
H	2.55700	-2.64700	7.39700

C	4.10400	-1.58400	6.37000
H	4.84100	-1.76400	7.14500
C	4.43800	-0.85600	5.23400
H	5.43400	-0.44500	5.11000
C	3.49000	-0.65000	4.24400
H	3.74000	-0.08300	3.35400
C	2.21500	-1.18700	4.38500
C	5.08400	-4.86000	-1.35900
H	4.85700	-5.37200	-2.29000
H	5.13700	-5.56100	-0.52900
O	-3.50500	3.79000	3.29900
O	-4.47600	4.01500	-1.13400
O	-1.26400	0.94400	-3.40800
N	-4.02200	3.89500	1.08700
C	-3.28600	3.40200	2.16700
C	-2.23600	2.40600	1.86300
C	-1.46500	1.92200	2.88500
H	-1.64600	2.27700	3.89300
C	-0.47100	0.97900	2.62800
H	0.11200	0.63000	3.45800
C	-0.22900	0.47000	1.35500
C	-1.00600	0.98100	0.26200
C	-0.79100	0.55900	-1.09300
C	-1.54300	1.19400	-2.09100
C	-2.51400	2.15800	-1.79200
H	-3.07100	2.64600	-2.58000
C	-2.75700	2.51800	-0.49900
C	-3.81100	3.52900	-0.23700
C	-2.00100	1.95700	0.54800
C	-1.83400	1.88900	-5.53100
C	-2.81800	2.08000	-6.51100
H	-2.55700	2.64700	-7.39700
C	-4.10400	1.58400	-6.37000
H	-4.84100	1.76400	-7.14500
C	-4.43800	0.85600	-5.23400
H	-5.43400	0.44500	-5.11000
C	-3.49000	0.65000	-4.24400
H	-3.74000	0.08300	-3.35400
C	-2.21500	1.18700	-4.38500
C	-5.08400	4.86000	1.35900

H	-4.85700	5.37200	2.29000
H	-5.13700	5.56100	0.52900
H	6.04600	-4.35000	-1.44800
H	-6.04600	4.35000	1.44800
O	-4.05200	8.27000	2.54900
O	-4.42300	8.41700	-1.97700
O	4.58700	1.00000	1.70300
O	3.67400	0.41900	-2.70600
O	-0.16000	3.48000	-3.72900
N	-4.25100	8.33700	0.29500
N	4.12500	0.68900	-0.49100
C	-3.63800	7.89800	1.46500
C	-2.49800	6.96700	1.32500
C	-1.86700	6.50900	2.45600
H	-2.22100	6.84100	3.42400
C	-0.80000	5.61600	2.34600
H	-0.34000	5.26500	3.25900
C	-0.36000	5.15200	1.11600
C	0.76900	4.22200	0.99700
C	1.53300	3.86300	2.09700
H	1.33500	4.31000	3.06100
C	2.56500	2.92800	2.01100
H	3.14600	2.65200	2.88300
C	2.84100	2.32600	0.80700
C	3.91000	1.30800	0.73400
C	3.42700	0.99100	-1.66200
C	2.37700	2.03200	-1.56000
C	1.63200	2.31700	-2.66800
H	1.81700	1.75800	-3.57500
C	0.62400	3.29300	-2.62200
C	0.33700	4.00900	-1.45700
C	-0.68900	5.06600	-1.36200
C	-1.35600	5.58700	-2.46400
H	-1.11100	5.24300	-3.45300
C	-2.37000	6.54200	-2.33300
H	-2.88400	6.92200	-3.20800
C	-2.75400	6.99100	-1.09700
C	-3.86400	7.96500	-0.99600
C	-2.08800	6.51500	0.05300
C	-1.03200	5.57400	-0.06800

C	1.06800	3.64900	-0.27600
C	2.10300	2.68000	-0.34000
C	0.32900	3.15100	-4.98000
C	1.55000	3.67600	-5.39100
H	2.10800	4.30200	-4.70400
C	2.04500	3.38500	-6.65100
H	3.00300	3.78800	-6.96100
C	1.30500	2.57800	-7.50700
H	1.68400	2.32900	-8.49200
C	0.07400	2.09200	-7.09800
H	-0.50300	1.46300	-7.76700
C	-0.45700	2.37000	-5.83000
C	-5.37000	9.26100	0.46000
H	-5.03300	10.16100	0.97600
H	-5.74900	9.50500	-0.52800
C	5.17500	-0.32500	-0.54900
H	5.12900	-0.93400	0.35100
H	5.01600	-0.92500	-1.44000
H	6.15800	0.14900	-0.59900
H	-6.14500	8.79100	1.06700

Supplementary References

[S1] W. Kabsch, *Acta Crystallogr. D* **66**, 125–132 (2010)
[S2] G. M. Sheldrick, *Acta Crystallogr. A* 71, 3–8 (2015)
[S3] G. M. Sheldrick, *Acta Crystallogr. C* 71, 3–8 (2015)
[S4] Evans, P. R. & Murshudov, G. N. *Acta Crystallogr.* D 69, 1204–1214 (2013)
[S5] Mahl, M., Niyas, M. A., Shoyama, K. & Würthner, F. *Nat. Chem.* 14, 457–462 (2022).
[S6] Seybold , G. & Wagenblast,G. *Dyes Pigm.* **11**, 303-317 (1989).
[S7] Hong, Y. *et al. J. Am. Chem. Soc.* **142**, 7845–7857 (2020).
[S8] Kim, T.; Kim, W.; Vakuliuk, O.; Gryko, D. T. & Kim, D. *J. Am. Chem. Soc.* **142**, 1564–1573 (2020).
[S9] Min, C. K. & Joo, T. *Opt. Lett.* **30**, 1855–1857 (2005).
[S10] Eom, I. & Joo, T. *J. Chem. Phys.* **131**, 244507 (2009).
[S11] Rhee, H. & Joo, T. *Opt. Lett.* **30**, 96–98 (2005).
[S12] Kuramochi, H., Takeuchi, S.& Tahara, T. *Rev. Sci. Instrum.* **87**, 043103 (2016).
[S13] Liebel, M. Schnedermann, C.& Kukura, P. *Opt. Lett.* **39**, 4112 (2014).
[S14] Grupp, A. *et al. Opt. (United Kingdom)* **20**, 104005 (2018).
[S15] Teo, S. M., Ofori-Okai, B. K., Werley, C. A. & Nelson, K. A. *Rev. Sci. Instrum.* **86**, 051301 (2015).
[S16] Kim, W. *et al. Angew.. Chem. Int. Ed.* **132**, 8649–8656 (2020).
[S17] a) Yanai, T., Tew, D. & Handy, N. *Chem. Phys. Lett.*, **393** 51-57 (2004). b) Hariharan P. C. & Pople, J. A. *Theor. Chem. Acc.*, **28** 213-222 (1973). c) Petersson G. A. *et al. J. Chem. Phys.* **89**, 2193-2218 (1988).
[S18] Kaufmann C., Bialas,D., Stolte,M. & Würthner,F. *J. Am. Chem. Soc.* **140**, 9986 (2018).
[S19] Gaussian 09, Revision D.01, M. J. Frisch, G. W. Trucks, H. B. Schlegel, G. E. Scuseria, M. A. Robb, J. R. Cheeseman, G. Scalmani, V. Barone, B. Mennucci, G. A. Petersson, H. Nakatsuji, M. Caricato, X. Li, H. P. Hratchian, A. F. Izmaylov, J. Bloino, G. Zheng, J. L. Sonnenberg, M. Hada, M. Ehara, K. Toyota, R. Fukuda, J. Hasegawa, M. Ishida, T. Nakajima, Y. Honda, O. Kitao, H. Nakai, T. Vreven, J. A. Montgomery, Jr., J. E. Peralta, F. Ogliaro, M. Bearpark, J. J. Heyd, E. Brothers, K. N. Kudin, V. N. Staroverov, T. Keith, R. Kobayashi, J. Normand, K. Raghavachari, A. Rendell, J. C. Burant, S. S. Iyengar, J. Tomasi, M. Cossi, N. Rega, J. M. Millam, M. Klene, J. E. Knox, J. B. Cross, V. Bakken, C. Adamo, J. Jaramillo, R. Gomperts, R. E. Stratmann, O. Yazyev, A. J. Austin, R. Cammi, C. Pomelli, J. W. Ochterski, R. L. Martin, K. Morokuma, V. G. Zakrzewski, G. A. Voth, P. Salvador, J. J. Dannenberg, S. Dapprich, A. D. Daniels, O. Farkas, J. B. Foresman, J. V. Ortiz, J. Cioslowski, and D. J. Fox, Gaussian, Inc., Wallingford CT, (2013).
[S20] Chai, J.-D. & Head-Gordon, M. *Phys. Chem. Chem. Phys.* **10**, 6615 (2008).
[S21] Weigend, F. & Weigend, R. Ahlrichs, *Phys. Chem. Chem. Phys. 7*, 3297 (2005).
[S22] Lu, T. & Chen,F. *J. Comput. Chem.***33**, 580 (2012).
[S23] Chang, J. C. *J. Chem. Phys.* **67**, 3901 (1977).
[S24] a) Scholes,G. D. & Ghiggino, K. P. *J. Phys. Chem.* 98, 4580 (1994). b) N. J. Hestand, F. C. Spano, *Acc. Chem. Res.* **50**, 341 (2017).
[S25] a) te Velde, G. *et al. J. Comput. Chem.* **22**, 931 (2001). b) Fonseca Guerra, C., Snijders, J. G., te Velde, G. & Baerends, E. J. *Theor. Chem. Acc.* **99**, 391 (1998). c) ADF2013, SCM, Theoretical Chemistry, Vrije, Universiteit, Amsterdam, The Netherlands, http://www.scm.com.
[S26] Perdew, J. P., Burke, K. & Wang, Y. *Phys. Rev. B* **54**, 16533 (1996).
[S27] Barbieri, P. L., Fantin, P. A. & Jorge, F. E. *Mol. Phys.* **104**, 2945 (2006).
[S28] a) Hestand,N. J. & Spano,F. C. *J. Chem. Phys* **143**, 244707 (2015). b) Kistler, K. A., Pochas, C. M., Yamagata, H., Matsika, S. & Spano, F. C. *J. Phys. Chem. B* **116**, 77 (2012).
[S29] Luzanov, A., Sukhorukov, A. & Umanskii, V. *Theor. Exp. Chem.* **10**, 354–361 (1976).
[S30] Tretiak, S. & Mukamel, S. *Chem. Rev.* **102**, 3171–3212 (2002).
[S31] Plasser,F. & Lischka, H. *J. Chem. Theory Comput.* **8**, 2777–2789 (2012).
[S32] Titov E. *Molecules* **26**, 4245 (2021).
[S33] a) F. Würthner *et al. J. Org. Chem.* **69**, 7933-7939 (2004). b) Zhang, S. *et al. J. Polym. Sci. Part A: Polym. Chem.* **51**, 1550-1558 (2013).
[S32] Casanova, *Int. J. Quantum. Chem.* **115**, 442 (2015)
[S33] Hoche et al., *Phys. Chem. Chem. Phys.* **19**, 25002 (2017)
[S34] Kang et al. *J. Am. Chem. Soc.* **143**, 9825 *(*2021)
[S35] Hong et al. *Angew. Chem. Int. Ed.* **61**, e202114474) (2022).
[S36] Luzanov, A. V.; Casanova, D.; Feng, X.; Krylov, A. I. *J. Chem. Phys*. **142**, 224104 (2015).
[S37] Shao, Y., Gan, Z., Epifanovsky, E., Gilbert, A. T. B., Wormit, M., Kussmann, J., Lange, A. W., Behn, A., Deng, J., Feng, X., Ghosh, D., Goldey, M., Horn, P. R., Jacobson, L. D., Kaliman, I., Khaliullin, R. Z., Kus, T., Landau, A., Liu, J., Proynov, E. I., Rhee, Y. M., Richard, R. M., Rohrdanz, M. A., Steele, R. P., Sundstrom, E. J., Woodcock, H. L., Zimmerman, P. M., Zuev, D., Albrecht, B., Alguire, E., Austin, B., Beran, G. J. O., Bernard, Y. A., Berquist, E., Brandhorst, K., Bravaya, K. B., Brown, S. T., Casanova, D., Chang, C., Chen, Y., Chien, S. H., Closser, K. D., Crittenden, D. L., Diedenhofen, M., DiStasio, R. A., Do, H., Dutoi, A. D., Edgar, R. G., Fatehi, S., Fusti-Molnar, L., Ghysels, A., Golubeva-Zadorozhnaya, A., Gomes, J., HansonHeine, M. W. D., Harbach, P. H. P., Hauser, A. W., Hohenstein, E. G., Holden, Z. C., Jagau, T., Ji, H., Kaduk, B., Khistyaev, K., Kim, J., Kim, J., King, R. A., Klunzinger, P., Kosenkov, D., Kowalczyk, T., Krauter, C. M., Lao, K. U., Laurent, A. D., Lawler, K. V., Levchenko, S. V., Lin, C. Y., Liu, F., Livshits, E., Lochan, R. C., Luenser, A., Manohar, P., Manzer, S. F., Mao, S., Mardirossian, N., Marenich, A. V., Maurer, S. A., Mayhall, N. J., Neuscamman, E., Oana, C. M., Olivares-Amaya, R., O'Neill, D. P., Parkhill, J. A., Perrine, T. M., Peverati, R., Prociuk, A., Rehn, D. R., Rosta, E., Russ, N. J., Sharada, S. M., Sharma, S., Small, D. W., Sodt, A., Stein, T., Stueck, D., Su, Y., Thom, A. J. W., Tsuchimochi, T., Vanovschi, V., Vogt, L., Vydrov, O., Wang, T., Watson, M. A., Wenzel, J., White, A., Williams, C. F., Yang, J., Yeganeh, S., Yost, S. R., You, Z., Zhang, I. Y., Zhang, X., Zhao, Y., Brooks, B. R., Chan, G. K. L., Chipman, D. M., Cramer, C. J., Goddard, W. A., Gordon, M. S., Hehre, W. J.,

Klamt, A., Schaefer, H. F., Schmidt, M. W., Sherrill, C. D., Truhlar, D. G., Warshel, A., Xu, X., Aspuru-Guzik, A., Baer, R., Bell, A. T., Besley, N. A., Chai, J., Dreuw, A., Dunietz, B. D., Furlani, T. R., Gwaltney, S. R., Hsu, C., Jung, Y., Kong, J., Lambrecht, D. S., Liang, W., Ochsenfeld, C., Rassolov, V. A., Slipchenko, L. V., Subotnik, J. E., Van Voorhis, T., Herbert, J. M., Krylov, A. I., Gill, P. M. W., Head-Gordon, M. *Mol. Phys*. **113**, 184 (2015)
